# NeoDesign: a computational tool for optimal selection of polyvalent neoantigen combinations

**DOI:** 10.1093/bioinformatics/btae585

**Published:** 2024-09-27

**Authors:** Wenqian Yu, Hongwu Yu, Jingjing Zhao, Hena Zhang, Kalam Ke, Zhixiang Hu, Shenglin Huang

**Affiliations:** Department of Integrative Oncology, Fudan University Shanghai Cancer Center, and Shanghai Key Laboratory of Medical Epigenetics, Institutes of Biomedical Sciences, Fudan University, Shanghai 200032, China; Department of Oncology, Shanghai Medical College, Fudan University, Shanghai 200032, China; Department of Integrative Oncology, Fudan University Shanghai Cancer Center, and Shanghai Key Laboratory of Medical Epigenetics, Institutes of Biomedical Sciences, Fudan University, Shanghai 200032, China; Department of Oncology, Shanghai Medical College, Fudan University, Shanghai 200032, China; Department of Integrative Oncology, Fudan University Shanghai Cancer Center, and Shanghai Key Laboratory of Medical Epigenetics, Institutes of Biomedical Sciences, Fudan University, Shanghai 200032, China; Department of Oncology, Shanghai Medical College, Fudan University, Shanghai 200032, China; Department of Integrative Oncology, Fudan University Shanghai Cancer Center, and Shanghai Key Laboratory of Medical Epigenetics, Institutes of Biomedical Sciences, Fudan University, Shanghai 200032, China; Department of Oncology, Shanghai Medical College, Fudan University, Shanghai 200032, China; Department of Integrative Oncology, Fudan University Shanghai Cancer Center, and Shanghai Key Laboratory of Medical Epigenetics, Institutes of Biomedical Sciences, Fudan University, Shanghai 200032, China; Department of Oncology, Shanghai Medical College, Fudan University, Shanghai 200032, China; Department of Integrative Oncology, Fudan University Shanghai Cancer Center, and Shanghai Key Laboratory of Medical Epigenetics, Institutes of Biomedical Sciences, Fudan University, Shanghai 200032, China; Department of Oncology, Shanghai Medical College, Fudan University, Shanghai 200032, China; Department of Integrative Oncology, Fudan University Shanghai Cancer Center, and Shanghai Key Laboratory of Medical Epigenetics, Institutes of Biomedical Sciences, Fudan University, Shanghai 200032, China; Department of Oncology, Shanghai Medical College, Fudan University, Shanghai 200032, China

## Abstract

**Motivation:**

Tumor polyvalent neoantigen mRNA vaccines are gaining prominence in immunotherapy. The design of sequences in vaccine development is crucial for enhancing both the immunogenicity and safety of vaccines. However, a major challenge lies in selecting the optimal sequences from the large pools generated by multiple peptide combinations and synonymous codons.

**Results:**

We introduce NeoDesign, a computational tool designed to tackle the challenge of sequence design. NeoDesign comprises four modules: Library Construction, Optimal Path Filtering, Linker Addition, and λ-Evaluation. It aims to identify the optimal protein sequence for tumor polyvalent neoantigen vaccines by minimizing linker usage, avoiding unexpected neoantigens and functional domains, and simplifying the structure. It also provides a preference scheme to balance mRNA stability and protein expression when designing mRNA sequences for the optimal protein sequence. This tool can potentially improve the sequence design of tumor polyvalent neoantigen mRNA vaccines, thereby significantly advancing immunotherapy strategies.

**Availability and implementation:**

NeoDesign is freely available on https://github.com/HuangLab-Fudan/neoDesign and https://figshare.com/projects/NeoDesign/221704.

## 1 Introduction

Neoantigens, specifically expressed in tumor cells, are increasingly recognized as effective and safe targets for immunotherapy, eliciting robust, and specific anti-tumor immune responses. Unlike traditional tumor-associated antigens, which have limited therapeutic impact and carry risks of autoimmune reactions, tumor-specific neoantigens offer significant advantages in designing cancer vaccines (Hu *et al.* 2018, Smith *et al.* 2019, Xie *et al.* 2023). Various vaccine forms, such as peptide, nucleic acid (DNA/mRNA), and dendritic cell (DC) vaccines, utilize these neoantigens (Peng *et al.* 2019, Lin *et al.* 2022, Niemi *et al.* 2022). mRNA vaccines are particularly notable for their rapid development, scalability, cost-effectiveness, and high efficacy, making them prime candidates for cancer vaccine research (Pardi *et al.* 2018, Esprit *et al.* 2020, Liu *et al.* 2023). Recent advancements have introduced a novel form of neoantigen vaccines termed polyvalent neoantigen vaccines. These vaccines have demonstrated therapeutic effectiveness and represent a potent strategy to enhance the immunogenicity of subunit vaccine neoantigens (Wei *et al.* 2017, Johanns *et al.* 2019), providing enhanced immunogenicity and broad-spectrum immune protection, thereby making them superior in tumor immunotherapy. To maximize the therapeutic effectiveness of polyvalent neoantigen mRNA vaccines, researchers have focused on optimizing various aspects, including adjuvants, schedules, delivery modes, and the precise prediction and selection of neoantigens, along with sequence design (Hung *et al.* 2007, Hacohen *et al.* 2013, Hundal *et al.* 2016, Hu *et al.* 2018). However, the challenge of sequence design remains in selecting the optimal sequence from the large pools generated by random peptide combinations and synonymous codons.

Currently, the only available tool, pVACvector, selects optimal sequences based on peptide combinations, primarily focusing on the reduction of junctional epitopes (Hundal *et al.* 2020). However, the presence of linkers may negatively impact vaccines. Primarily, linkers increase the length of mRNA vaccines, correlating with faster degradation due to factors such as stem-loop structures that can cause ribosomal stalling (Doma and Parker 2006, Tollervey 2006, Feng and Niu 2007). Moreover, the repetitive sequences introduced by linkers may form hairpin structures that hinder RNA transcription and translation (Leppek *et al.* 2018, Qian *et al.* 2021, Casas-Delucchi *et al.* 2022). Recent research has demonstrated that completely omitting linkers can substantially improve the immunogenicity of vaccines (Gurung *et al.* 2024). Complex protein structures may require larger proteasomes for efficient degradation, as tightly folded proteins can impede this process unless they possess unstructured regions (Prakash *et al.* 2004, Piwko and Jentsch 2006, Baugh *et al.* 2009, Kardon *et al.* 2015). Functional domains that interact with other proteins such as NAD(P)H quinone oxidoreductase 1 (NQO1), can inhibit proteasome cleavage, complicate neoantigen generation, and trigger unpredictable immune responses, thereby complicating vaccine design (Asher and Shaul 2005, Moscovitz *et al.* 2012, Lata *et al.* 2018, Wang *et al.* 2020). These factors are not incorporated in pVACvector, highlighting the need for developing a new tool that can optimize tumor polyvalent neoantigen vaccines while addressing these considerations. In addition, the LinearDesign tool offers a state-of-the-art method to select optimal mRNA from large pools generated by synonymous codons. This method integrates mRNA stability with protein expression, targeting previously unexplored areas of optimization (Zhang *et al.* 2023). Nevertheless, the optimization outcomes of LinearDesign heavily depend on the parameter λ, which balances mRNA stability with protein expression. Furthermore, research has shown that achieving both high stability and high protein expression in mRNA design are inherently contradictory goals (Leppek *et al.* 2022). Therefore, identifying an optimal λ value that reconciles these aspects is crucial for designing effective mRNA sequences. In summary, existing techniques that address the challenges posed by peptide combinations and synonymous codon usage are limited, and no unified pipeline effectively tackles both issues.

We have developed a computational tool, NeoDesign, to select optimal sequences from pools generated by multiple peptide combinations and to recommend a λ value that balances mRNA stability with protein expression. This tool comprises four modules: Library Construction, Optimal Path Filtering, Linker Addition, and λ-Evaluation. It efficiently selects an optimal protein sequence by integrating key factors such as minimizing linker usage, simplifying protein structures, and reducing the incidence of unexpected neoantigens and functional domains. In addition, NeoDesign provides guidelines to balance mRNA stability and protein expression in further mRNA sequence design, based on the optimal protein sequence. This system enhances both protein and mRNA sequence optimization, significantly advancing tumor polyvalent neoantigen vaccine development.

## 2 Materials and methods

### 2.1 Methods and materials used to implement NeoDesign

**Figure 1.**
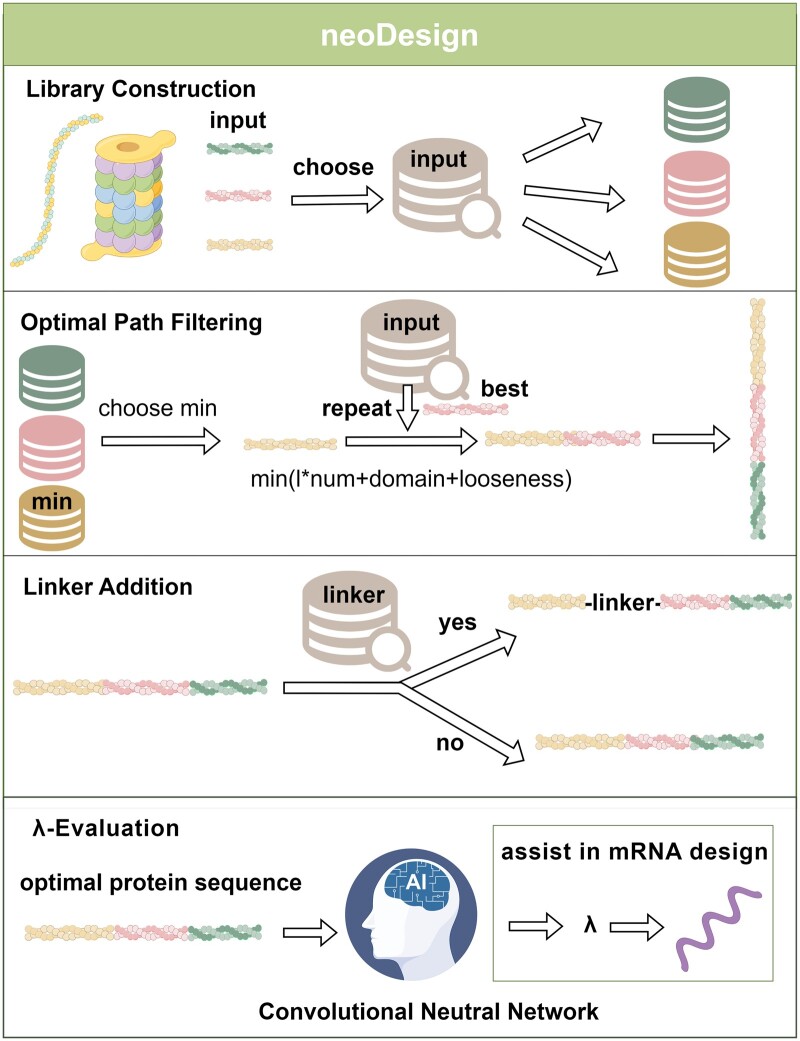
NeoDesign structure. The NeoDesign pipeline comprises four main modules. The input library consists of neoantigen peptides, with color coding that matches peptides to corresponding optional libraries during library construction. Decision-making on peptide additions is guided by the decision function (l*num+domain+looseness), selecting peptides that minimize this function’s value. This process repeats until all peptides from the input library have been incorporated. The linker library includes common linkers, and the Linker Addition module is tasked with integrating linkers at crucial sites. Finally, the λ-Evaluation module calculates a suitable λ value for the optimal protein sequence which assists in further mRNA design (By Figdraw).

NeoDesign utilizes high-quality neoantigen peptides as its input data. Typically, neoantigen vaccines contain 20–30 unique peptides (Ott *et al.* 2020, Awad *et al.* 2022), derived from mutations identified either by whole-exome sequencing (WES) and predictive algorithms (Garcia-Garijo *et al.* 2019) or through mass spectrometry-based methods (Yadav *et al.* 2014, Abelin *et al.* 2017). These peptides form an input library, depicted as a brown-colored input library in Fig. 1, which serves as the basis for subsequent analyses.

A neoantigen prediction pipeline integrating various neoantigen prediction methods is being developed to predict potential neoantigens at peptide junctions. The pipeline consists of Pepsickle (Weeder *et al.* 2021), NetChop (version 3.1) (Nielsen *et al.* 2005), NetMHCpan (version 4.1) (Reynisson *et al.* 2020), and MHCflurry (version 2.0) (O’Donnell *et al.* 2020), taking into account factors such as proteasome cleavage and MHC peptide binding affinities. The neoantigen prediction pipeline plays an important role in each module of NeoDesign. In the Optimal Path Filtering module, the greedy algorithm uses a decision function to guide the sequential addition of neoantigen peptides, selecting the most favorable peptide at each step to ultimately determine the optimal protein sequence. The decision function incorporates several essential criteria for an optimal sequence, including minimal linker usage, reduced unexpected neoantigens and functional domains, and a flexible protein structural configuration. The default linker library in the Linker Addition module is built to add linkers, with customization options available to users to suit specific requirements. In the λ-Evaluation module of NeoDesign, a convolutional neural network-based prediction model is integrated with the advanced protein representation model ProtTransT5-XL-UniRef50. This integration facilitates the extraction of high-dimensional embeddings from protein sequences, essential for determining the optimal λ ranges for new protein sequences. To ensure the robustness and validity of this approach, extensive comparisons are conducted against baseline models, including Random Forests, Bayesian models, Support Vector Machines (SVMs), and Multilayer Perceptrons (MLPs). In addition, ProtTransT5-XL-UniRef50 is compared with leading protein representation models from the ESM series, ProtBERT, and ProtTransT5-XL-BFD. The methodologies, including comparative analyses, are elaborated in the Supplementary Material (Supplementary Figs S4–S10; Supplementary Tables S1–S7).

### 2.2 Methods and materials used to evaluate NeoDesign

To evaluate the implementation of NeoDesign, 100 target proteins, and 100 reference proteins are utilized. 100 patient samples from individuals with pancreatic and lung cancer are retrieved from The Cancer Genome Atlas (TCGA) public database, specifically selecting for neoantigen peptides derived from single nucleotide variants (SNVs). For practicality, the number of peptides per patient is limited to between 10 and 30. The 100 samples are processed by NeoDesign, resulting in 100 target proteins. As a control, 100 known functional proteins, including ubiquitin protein ligase E3A and DNA damage binding protein, are randomly selected as 100 reference proteins from the Protein Data Bank (PDB).

The properties of the optimal protein sequences generated by NeoDesign, including linker numbers, peptide numbers, domain numbers, and looseness values, are evaluated on 100 target proteins. In addition, domain numbers and looseness values are calculated for 100 reference proteins to facilitate comparison with the target proteins. Several metrics including MFE Relative Difference Ratio, CAI Relative Difference Ratio, and Gain, are defined to evaluate the effectiveness of our recommended λ parameter compared to the default λ in LinearDesign. For performance evaluation, NeoDesign is run on two servers with different configurations. Ultimately, NeoDesign is compared with the existing tool pVACvector, in terms of results and running time. Neoantigens from 100 patient samples retrieved from The Cancer Genome Atlas (TCGA) public database are processed by pVACvector, resulting in the generation of 100 comparison sequences. A comparison of linker numbers is conducted between the 100 comparison sequences generated by pVACvector and the 100 target sequences generated by NeoDesign. The differences in the number of linkers, domains, and looseness values produced by each tool are evaluated. Details of the methods used to evaluate NeoDesign’s results are described in the Supplementary Materials.

## 3 Results

### 3.1 NeoDesign structure

We have developed NeoDesign, a computational tool designed to identify optimal protein sequences from pools generated by various neoantigen peptide combinations. This tool also provides strategies for balancing mRNA stability with protein expression in mRNA sequence design. Figure 1 illustrates the structure of NeoDesign, which accepts high-quality neoantigen peptides as input. The tool consists of four primary modules: Library Construction, Optimal Path Filtering, Linker Addition, and λ-Evaluation.

#### 3.1.1 Library construction

The input data consists of high-quality neoantigen peptides. In this module, we have uniquely defined the new concept of optional libraries (Supplementary Fig. S1). The purpose of the module is to compute front-end and back-end optional libraries for each peptide sequence in the input data. The front-end optional library for each peptide includes other peptide sequences from the input data that can be concatenated pairwise at the front with the specific peptide, adhering to specific conditions. Similarly, the back-end optional library contains peptides that can be concatenated pairwise at the back with the specific peptide, satisfying the same criteria. The key condition for including peptides in these libraries is that their pairwise concatenation with a specific peptide does not generate unexpected neoantigens, as determined by the neoantigen prediction pipeline. Peptides that meet this criterion are included in the optional library of the specific peptide, indicating their suitability for concatenation with the specific peptide. The outputs are the front-end and back-end optional libraries for each peptide, which are then utilized in the Optimal Path Filtering module. The Library Construction module ensures that any peptide selects other peptides from its corresponding optional library for concatenation will not generate unexpected neoantigens and will minimize linkers, thus facilitating subsequent steps in the NeoDesign pipeline, including Optimal Path Filtering and Linker Addition.

#### 3.1.2 Optimal path filtering

In the Optimal Path Filtering module (Supplementary Fig. S2), the inputs are the front-end optional libraries and back-end optional libraries of all peptides from the initial input data. These libraries undergo statistical analysis to evaluate and rank them based on the number of optional peptides each contains.

The initial step in optimal path selection involves identifying the sequence’s starting point, determined through statistical analysis of the optional libraries. The peptide whose optional library contains the smallest number of optional peptides (at least one) is selected as the starting point. This step ensures flexibility in subsequent path selection and minimizes the occurrence of the linker.

Once a sequence starting point is established, the initial short sequence is generated in different lengths based on the parity of the number of neoantigen peptides in the input data. This approach facilitates the sequential addition of peptides to both ends of the sequence simultaneously, thereby reducing the time complexity. Specifically, if the number of peptides is odd, the sequence begins with three concatenated peptides; if even, it starts with two. A greedy algorithm is used to select peptides at each step, based on a defined decision function (l*num+domain+looseness) detailed in the Supplementary Methods. Peptides are iteratively added to both ends of the sequence, selecting the peptide that yields the lowest value of the decision function at each step. Each addition of a peptide decreases the total number of peptides in the input data by one. This process is repeated until no peptides remain in the input data, culminating in the determination of the optimal path and the final optimal sequence.

#### 3.1.3 Linker addition

The third module in the NeoDesign pipeline is the Linker Addition module, as depicted in Fig. 1. While the optimal path selection process strives to minimize the occurrence of unexpected neoantigens and maintain a loose structure without linkers, there remain specific regions in the sequence where avoiding unexpected neoantigens is challenging without incorporating a linker.

The module analyzes the sequence to identify regions requiring linkers and screens the linker library to select appropriate ones. These linkers are chosen to guarantee that the sequence does not produce unexpected neoantigens after their addition. Ultimately, this process yields an optimal protein sequence that minimizes linker usage. Incorporating this step allows NeoDesign to more effectively address any remaining challenges in avoiding unexpected neoantigens. The finalized optimal protein sequence is then utilized in the λ-Evaluation module.

#### 3.1.4 λ-Evaluation

The λ-Evaluation module utilizes a trained convolutional neural network to predict a range of λ values for the optimal protein sequence. This range provides a recommended λ parameter for LinearDesign, assisting in the optimization of the balance between mRNA stability and protein expression within mRNA sequence design. This functionality is crucial for designing tumor neoantigen mRNA vaccines as it aids in achieving an optimal balance between mRNA stability and protein expression. Detailed explanations of the module’s theoretical foundations, data preparation, and model building are available in the Supplementary Material.

### 3.2 Results evaluation for NeoDesign implementation

The evaluation of NeoDesign results encompasses four aspects: assessment of the properties of the generated optimal protein sequences; analysis of the impact of the recommended λ parameters on RNA stability and protein expression; evaluation of the generalization capabilities of NeoDesign across different computing platforms; and comparison with existing tools.

The evaluation of the properties of the optimal protein sequences generated by NeoDesign takes into account several parameters across 100 target proteins, including the number of linkers and peptides, distribution of functional domains, and looseness parameters (Supplementary Fig. S3A–C). The findings indicate that the majority of these protein sequences contain no linkers (62 sequences), while a minority have one to three linkers (Supplementary Fig. S3A). The correlation between the number of peptides and the number of linkers is weak (*P* = 0.245). The analysis of functional domains reveals a stark contrast: The target protein sequences typically lack functional domains, whereas the reference sequences exhibit multiple domains. The difference is statistically significant, as evidenced by a Wilcoxon signed-rank test with a highly significant *P*-value of 3.28e−38 (Supplementary Fig. S3B). Furthermore, the looseness function values are generally lower in target protein sequences compared to those in reference sequences, a disparity confirmed by another significant Wilcoxon test *P*-value of 2.23e−08 (Supplementary Fig. S3C).

In addition, NeoDesign’s recommended λ parameters are compared with the default parameters of LinearDesign. The comparison reveals that 93% of sequences exhibit a gain value > zero, indicating superior performance with NeoDesign’s parameters (Supplementary Fig. S3D and E). When using 20 neoantigen peptides as input, Server 1 utilizes only 0.1% of both CPU and memory resources, with a processing time of 90 min. Server 2 consumes 0.2% of CPU and negligible memory resources, achieving a shorter processing time of 60 min. Both servers process data efficiently, suggesting the universality of NeoDesign for different performance scenarios.

Comparative analysis with pVACvector underscores the superiority of NeoDesign in minimizing linker generation. The majority of sequences generated by NeoDesign contain zero linkers, in stark contrast to pVACvector, which typically produces between 10 to 20 linkers per sequence as documented in Supplementary Fig. 3F.

In the pVACvector method, designed sequences typically incorporate numerous linkers. The inserted linkers themselves do not contain structural domains, thereby helping to reduce the overall number of structural domains and resulting in a looser structure than that of NeoDesign, as documented in Supplementary Tables online (GitHub repository of Huanglab-Fudan). Moreover, NeoDesign achieves task execution three times faster than pVACvector.

Overall, these results, supported by specific statistical data in the Supplementary Materials, confirm NeoDesign’s effectiveness in optimizing protein sequences and further enhancing mRNA design with minimal resource consumption and high processing speed.

## 4 NeoDesign implementation and availability

NeoDesign is capable of generating optimal protein sequences for multiple peptides using the provided code (1). Users can generate the optimal protein sequence for a specific sample by inputting a text file, as demonstrated with the example TCGA-US-A77E-01A.txt. The parameters provided are recommendations but can be freely adjusted to meet user-specific requirements. The 100 target protein sequences discussed in this paper are derived from peptides in TCGA database samples using NeoDesign, with the execution code listed as (1). The specific original peptide file is stored at https://github.com/HuangLab-Fudan/NeoDesign/tree/main/supplementary_data/TCGA_original_data.

NeoDesign is designed to output corresponding λ values for protein sequences. To facilitate this, users must organize the protein sequences into a file named target_protein_sequence.txt, which should be located in the same directory as the *lambda_evaluation.py* script. By running the code specified in (2), NeoDesign calculates the optimal λ values for each protein sequence. The λ values for 100 target proteins, generated through this process, are detailed in the results, which are stored on GitHub at HuangLab-Fudan’s NeoDesign repository. These results are also included in the Supplementary Materials.


(1) $$\begin{array}{c} \text{python}3\;\text{main}.\text{py}-p\;\text{example}/\text{TCGA}-\text{US}\\ -A77E-01A.\text{txt}-l\;1-s\;\text{human}-v\\ 0-e\;1e-5-e1\;0.05-e2\;0.05-e3\;0.05\\ -e4\;0.5-e5\;0.5-c\;2-d\\ /\text{home}/\text{NeoDesign}/\text{Pfam}-A.\text{hmm}\\ -d2/\text{home}/\text{NeoDesign}/\text{netMHCpan}\\ -4.1/\text{netMHCpan}-d3/\text{home}/\text{NeoDesign}/\text{netchop}\\ -3.1/\text{netchop} \end{array}$$

(2) python lambda_evaluation.py

The NeoDesign tool is freely available for academic and research use under an open-source license. The tool, along with documentation and a user guide, can be downloaded from our GitHub repository at https://github.com/HuangLab-Fudan/NeoDesign. It can also be downloaded from Figshare at https://figshare.com/projects/NeoDesign/221704. This ensures that researchers and developers can easily access and use the tool to advance their vaccine development projects.

## 5 Conclusion

Our study introduces NeoDesign, a computational tool specifically developed to optimize the selection of polyvalent neoantigen combinations. Neoantigens hold significant promise as cancer-specific immunotherapy targets, especially in the design of cancer vaccines. Our work presents unique algorithms and concepts, such as the construction of a peptide’s optional library and a defined decision function. All built-in tools in NeoDesign have been carefully selected based on both literature evidence and empirical comparisons (Supplementary Fig. S13 and Supplementary Discussion). These advancements address significant shortcomings in the current tool pVACvector by minimizing linker usage, streamlining protein structures, and avoiding functional domains. Furthermore, the tool provides a recommended λ parameter for the optimal protein sequence. The recommended λ parameter can be applied in LinearDesign to achieve a balance between mRNA stability and protein expression in the mRNA sequence design of the optimal protein sequence.

However, we primarily focus on MHC class I neoantigens, excluding MHC class II neoantigens from consideration. In previous studies, the high polymorphism of MHC-II genes represents an important hurdle toward accurate prediction and identification of T cell epitopes. Furthermore, many computational methods suffer from a lack of sufficient training data. These challenges in developing MHC II neoantigen prediction algorithms lead to a poor performance of current MHC II neoantigen prediction algorithms (Racle *et al.* 2023, Wang *et al.* 2023). Thus, the prediction method for MHC class II is not incorporated into our tool at this time. Moreover, the λ-Evaluation module uses a convolutional neural network. The performance of this model is contingent upon the quality of the training data. The scarcity of publicly available datasets containing mRNA sequences with experimentally determined stability and protein expression means we can only train on a limited number of datasets, which has hindered our model’s ability to achieve better performance. Therefore, the accuracy of predicting the recommended λ needs to be improved. The recommended λ serves primarily as a reference for practical application in LinearDesign. The actual efficacy of an mRNA vaccine will be influenced by a broader range of factors, including untranslated region components, and mRNA modification, and is not confined to the single parameter λ (Asrani *et al.* 2018, Kim *et al.* 2022, Gong *et al.* 2023, Mei and Wang 2023). Besides, experiments are needed to enhance the reliability of NeoDesign, evaluating the immunogenicity and efficacy of the designed tumor polyvalent neoantigen vaccine sequences in preclinical models or clinical trials.

In conclusion, this study marks the first systematic attempt to tackle the challenges in vaccine sequence design arising from the combination of multiple peptides and the use of synonymous codons. It provides a foundational sequence framework for the design and optimization of diversified neoantigen vaccines, including mRNA, DNA, dendritic cell (DC), and peptide vaccines. This work offers valuable insights and recommendations for further development in mRNA vaccine design, thereby contributing significantly to cancer immunotherapy.

## Supplementary Material

btae585_Supplementary_Data
