# Supplementary material for: NeoDesign: a computational tool for optimal selection of polyvalent neoantigen combinations
Source: Bioinformatics. 2024 Sep 27;40(10):btae585. doi: 10.1093/bioinformatics/btae585 (PMC11471261; doi:10.1093/bioinformatics/btae585)
Supplement: btae585_Supplementary_Data [file btae585_supplementary_data.zip › Supplementary Figure 9.pdf]

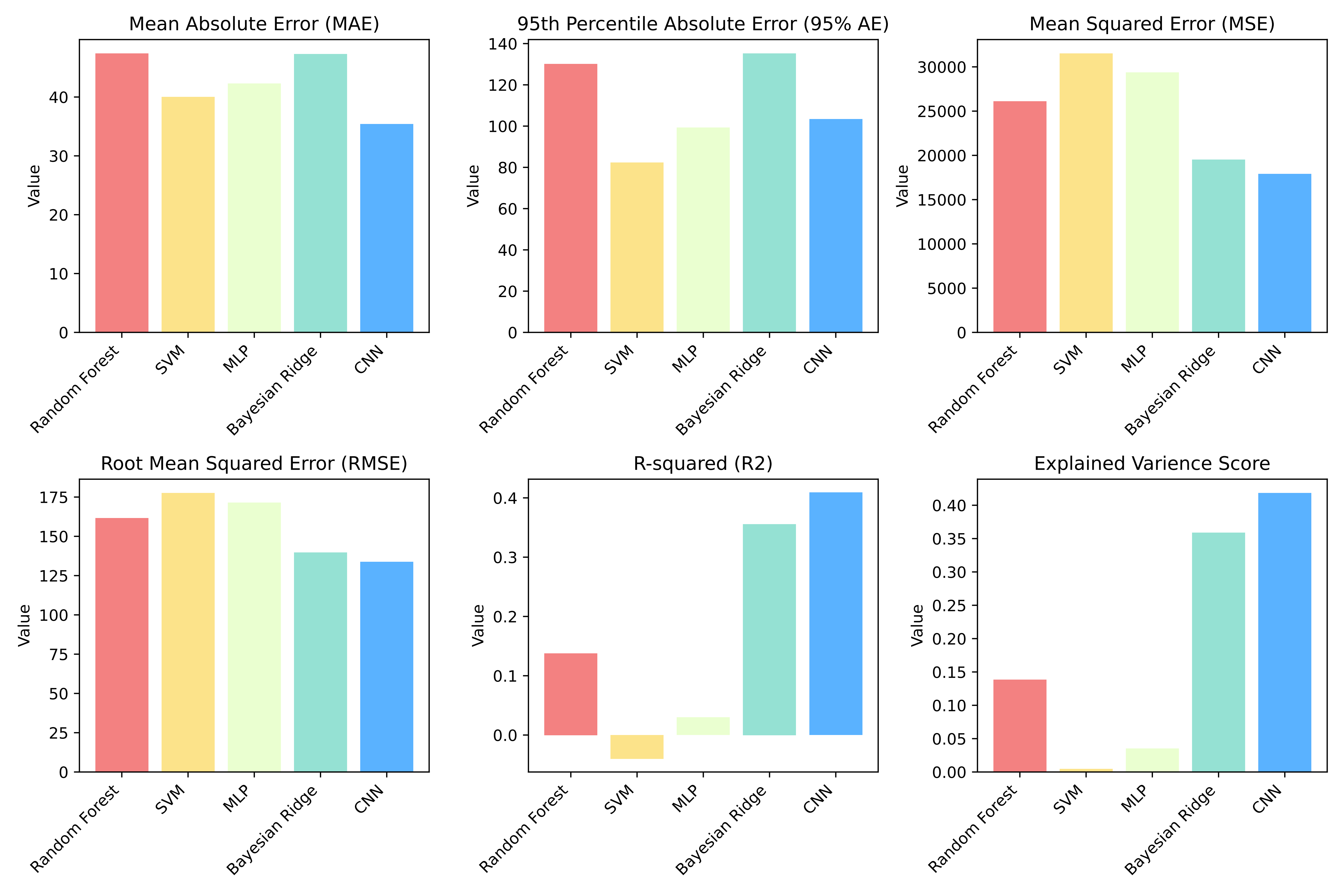
Mean Absolute Error (MAE)
95th Percentile Absolute Error (95% AE)
Mean Squared Error (MSE)
Root Mean Squared Error (RMSE)
R-squared (R2)
Explained Varience Score
Value
Random Forest
SVM
MLP
Bayesian Ridge
CNN
