# Supplementary material for: NeoDesign: a computational tool for optimal selection of polyvalent neoantigen combinations
Source: Bioinformatics. 2024 Sep 27;40(10):btae585. doi: 10.1093/bioinformatics/btae585 (PMC11471261; doi:10.1093/bioinformatics/btae585)
Supplement: btae585_Supplementary_Data [file btae585_supplementary_data.zip › Supplementary Figure 3.pdf]

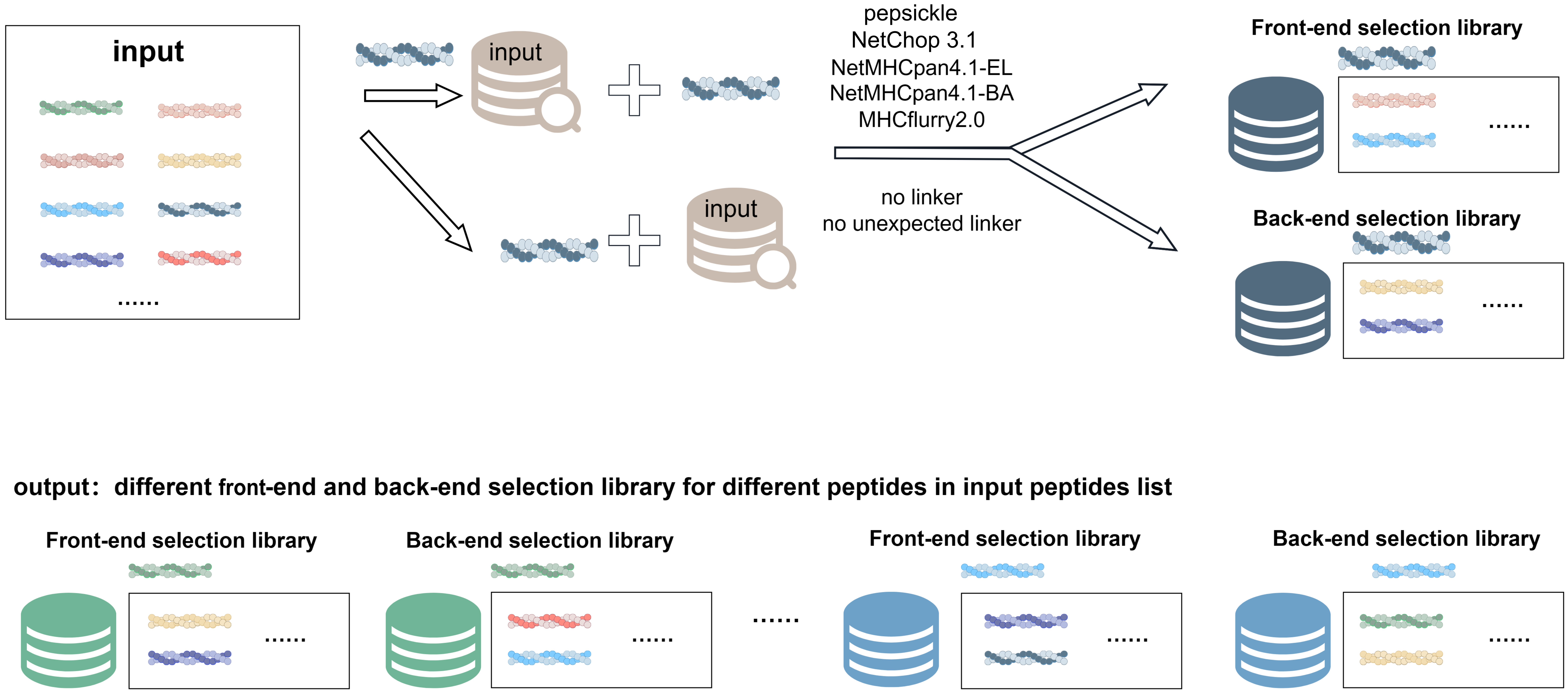
input
......
input
pepsickle
NetChop 3.1
NetMHCpan4.1-EL
NetMHCpan4.1-BA
MHCflurry2.0
no linker
no unexpected linker
input
Front-end selection library
......
Back-end selection library
......
output： different front-end and back-end selection library for different peptides in input peptides list
Front-end selection library
......
Back-end selection library
......
......
Front-end selection library
......
Back-end selection library
......
