# Supplementary material for: NeoDesign: a computational tool for optimal selection of polyvalent neoantigen combinations
Source: Bioinformatics. 2024 Sep 27;40(10):btae585. doi: 10.1093/bioinformatics/btae585 (PMC11471261; doi:10.1093/bioinformatics/btae585)
Supplement: btae585_Supplementary_Data [file btae585_supplementary_data.zip › Supplementary Figure 11.pdf]

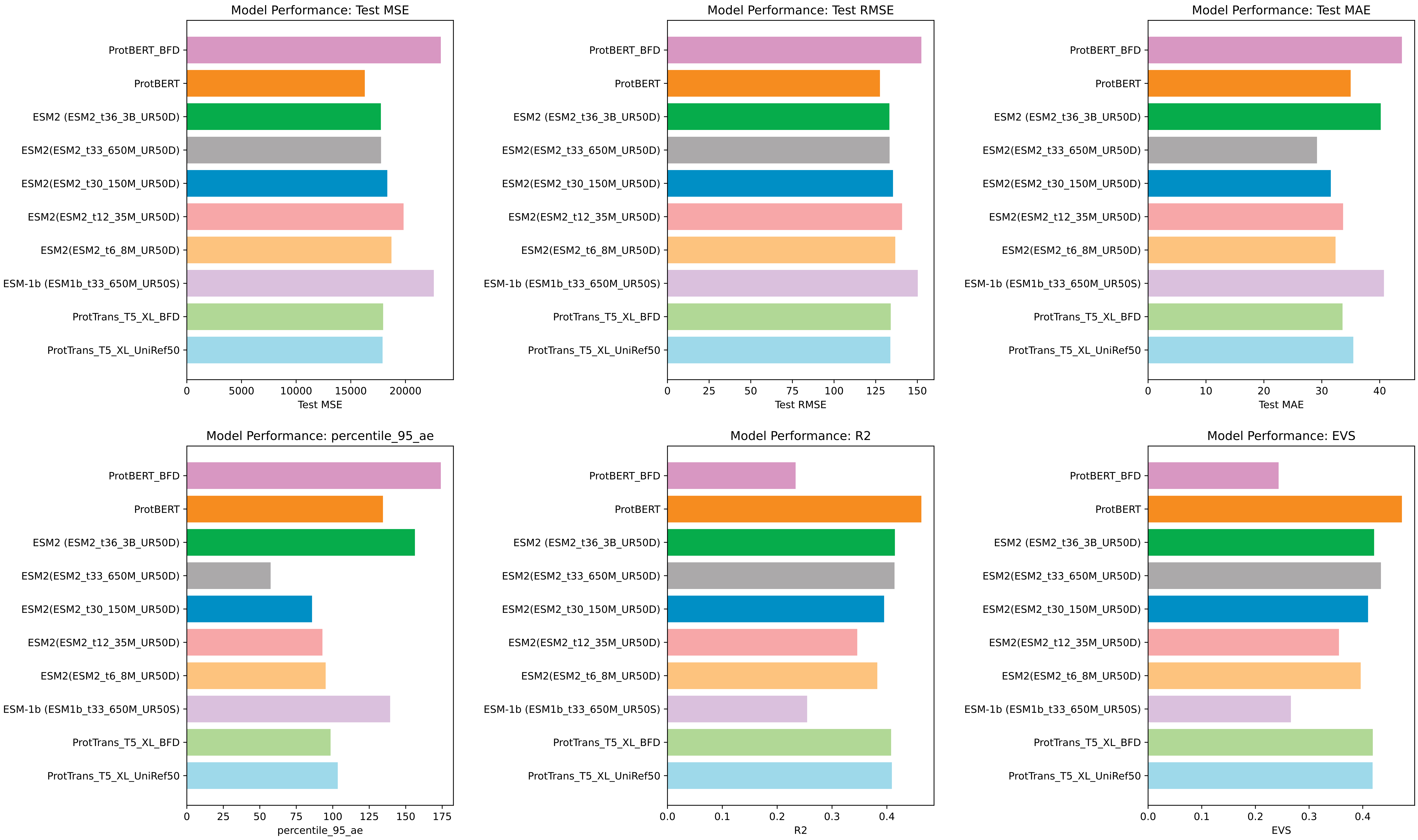
Model Performance: Test MSE
ProtBERT_BFD
ProtBERT
ESM2 (ESM2_t36_3B_UR50D)
ESM2(ESM2_t33_650M_UR50D)
ESM2(ESM2_t30_150M_UR50D)
ESM2(ESM2_t12_35M_UR50D)
ESM2(ESM2_t6_8M_UR50D)
ESM-1b (ESM1b_t33_650M_UR50S)
ProtTrans_T5_XL_BFD
ProtTrans_T5_XL_UniRef50
0 5000 10000 15000 20000
Test MSE
Model Performance: Test RMSE
0 25 50 75 100 125 150
Test RMSE
Model Performance: Test MAE
0 10 20 30 40
Test MAE
Model Performance: percentile_95_ae
0 25 50 75 100 125 150 175
percentile_95_ae
Model Performance: R2
0.0 0.1 0.2 0.3 0.4
R2
Model Performance: EVS
0.0 0.1 0.2 0.3 0.4
EVS
