# Supplementary material for: NeoDesign: a computational tool for optimal selection of polyvalent neoantigen combinations
Source: Bioinformatics. 2024 Sep 27;40(10):btae585. doi: 10.1093/bioinformatics/btae585 (PMC11471261; doi:10.1093/bioinformatics/btae585)
Supplement: btae585_Supplementary_Data [file btae585_supplementary_data.zip › bioinformatics_supplementary_data_clean_version.docx]

# Supplementary Information to:

NeoDesign: A Computational Tool for Optimal Selection of Polyvalent Neoantigen Combinations

# Supplementary Results

## Results evaluation for NeoDesign implementation

### 1.1 Evaluation of NeoDesign's optimal protein sequences

We evaluated various parameters for the 100 target proteins, including the number of linkers, the number of peptides, the distribution of functional domain numbers, and the looseness function values (Supplementary Figure 3). Additionally, we compared these 100 target proteins to an equal number of reference proteins in terms of domain numbers and looseness function values. Our results indicate that our final designed sequences aim to minimize linker usage: 62 sequences contain no linkers, 31 have one linker, 6 have two linkers, and only one sequence contains three linkers. Despite the expectation that more peptides would correlate with more linkers, we observed only a weak correlation (0.245) between these parameters (Supplementary Figure 3A).

When assessing the presence of functional domains, our target proteins uniformly had a functional domain value of 0, contrasting sharply with the varied domain values in the reference proteins. The Wilcoxon test confirms this difference as extremely significant, with a p-value of 3.28e-38 (Supplementary Figure 3B).

Furthermore, the looseness function values of our target proteins were comparatively low, aligning with the idea that lower values are preferable. In contrast, the reference proteins exhibited higher looseness values. The Wilcoxon test again showed a highly significant difference, with a p-value of 2.23e-08 (Supplementary Figure 3C).

These findings underscore that our target protein sequences effectively minimize the generation of functional domains and linkers while achieving the desired structural tightness. This marks significant advancements in protein design.

### 1.2 Evaluation of NeoDesign's recommended λ

We utilized the NeoDesign tool to generate recommended λ parameters for 100 target protein sequences. To assess the efficacy of these parameters compared to the default λ parameters in LinearDesign, we computed the MFE relative difference ratios, CAI relative difference ratios, and gain values for these sequences (Methods). The results show that 93% of the sequences achieved a gain value greater than 0, indicating that the recommended λ values from NeoDesign likely provide superior outcomes compared to the default settings in LinearDesign (Supplementary Figures 3D-E).

### 1.3 Evaluation of NeoDesign’s performance

We selected 20 neoantigen peptides as input data. The resource consumption of processing these peptides by NeoDesign on Server 1 is low, utilizing only 0.1% of CPU and memory resources. The program has a virtual memory size of 1,466,652 KB and a resident set size of 10,412 KB, both of which are small. The runtime on Server 1 is approximately 90 minutes. Overall, the program demonstrates low resource consumption and efficient performance on Server 1.

On Server 2, the program consumes 0.2% of CPU resources and 0.0% of memory resources. It has a virtual memory size of 2,465,096KB and a resident set size of 54,260 KB. Similar to Server 1, the program exhibits low resource consumption on Server 2. The runtime on Server 2 is approximately 60 minutes, which is faster than Server 1. The runtime on both Server 1 and Server 2 is relatively fast, and with fewer peptides in the input data, the runtime could be even shorter.

These findings demonstrate the versatility of NeoDesign, making it suitable for both average and high-performance scenarios. Furthermore, NeoDesign exhibits fast processing speeds and minimal resource consumption on both servers. It is important to note that all performance evaluations were conducted with the 'cpu' parameter set to 2. When the 'CPU' parameter is set higher, CPU resource consumption will increase, resulting in faster execution speeds.

A comparison was conducted between the 100 comparison sequences generated by pVACvector and the 100 target sequences generated by NeoDesign. The results demonstrated that NeoDesign outperforms pVACvector in reducing linker generation (Supplementary Figure 3F). The number of linkers generated by NeoDesign ranges from 0 to 3, with the majority being 0. In contrast, the number of linkers generated by pVACvector ranges from 10 to 20. In the pVACvector method, designed sequences typically incorporate numerous linkers. The inserted linkers do not contain structural domains, thereby helping to reduce the overall number of structural domains and resulting in a looser structure compared to NeoDesign (Supplementary Tables online). We also recorded the runtime of the same task on NeoDesign and pVACvector and found that NeoDesign is three times faster than pVACvector.

All specific statistical data and information can be found in Supplementary Tables online (<https://github.com/HuangLab-Fudan/neoDesign/tree/main/supplementary_data>).

Overall, these findings are consistent with our initial expectations, supporting the effectiveness of NeoDesign in achieving its intended purpose.

## Supplementary notes on λ-Evaluation

The module provides guidance on mRNA stability and protein expression preferences during the design process for mRNAs encoding the optimal protein sequences generated by the three modules described above.

### 2.1 Significance of the λ Value

Executing λ-Calculation for an mRNA sequence indicates that this mRNA achieves a better balance between mRNA free energy and the Codon Adaptation Index (CAI) compared to other sequences composed of synonymous codons (Methods). The calculated λ value represents the optimal balance between free energy and CAI for the corresponding protein sequence when it is designed as an mRNA sequence. The datasets we collected come from vaccine data, which likely include highly efficient mRNA sequences. By identifying the λ values of these efficient mRNAs, we can determine the optimal free energy and CAI balance scheme for the corresponding protein sequences during mRNA design. Based on these data, we can further develop predictive models to forecast the λ values for new proteins under the optimal free energy and CAI balance scheme.

However, before proceeding, we need to verify whether the λ value is closely related to the mRNA stability and protein expression. To this end, we calculated λ values for the collected datasets (Methods) and performed differential analysis of these λ values based on mRNA degradation scores and protein expression levels using datasets representing mRNA stability and protein expression (Methods). This analysis aims to determine the correlation between λ values and both mRNA stability and protein expression levels, thereby validating whether λ can serve as an effective parameter for balancing mRNA stability and protein expression. In our analysis, we observed significant differences in the minimum and maximum λ values between groups with high and low mRNA degradation scores, as well as between groups with high and low protein expression levels (Supplementary Figures 4A-B). These differences support the hypothesis that λ is significantly correlated with both mRNA stability and protein expression levels, validating λ as a reliable parameter for optimizing mRNA sequences to balance stability and efficient protein production.

### 2.2 Prediction model for λ values

The λ-Evaluation module includes a prediction model based on a trained convolutional neural network designed to predict the λ range (Supplementary Figure 4C). We use the same convolutional neural network configuration to predict the minimum and maximum values of λ separately. The specific model training and validation process is shown in Supplementary Figure 5. We use the Mean Absolute Error (MAE) as the loss function and Coefficient of Determination (R^2^) to determine the optimal epoch for early stopping. After completing the training and validation process, we successfully built a prediction model to execute our λ range prediction task.

### 2.3 Evaluation of the prediction model for λ values

#### 2.3.1 Performance on the independent test set

The evaluation results on the independent test set indicate that the λ-min and λ-max prediction models demonstrate solid performance, as detailed in Supplementary Table 1. Specifically, the λ-min prediction model achieved a Mean Absolute Error (MAE) of 27.21, while the λ-max prediction model had an MAE of 35.44. The corresponding Mean Squared Errors (MSE) were 11,468.81 and 17,905.80, with Root Mean Squared Errors (RMSE) of 107.09 and 133.81, respectively. Additionally, the Coefficient of Determination(R²) values for the λ-min and λ-max models were 0.63 and 0.41, and the Explained Variance Scores (EVS) were 0.64 and 0.42. The 95th Percentile Absolute Errors (95% AE) were 118.55 and 103.36, respectively. Supplementary Figure 6 further illustrates the comparison between actual and predicted values, showcasing the predictive accuracy of the convolutional neural network models for λ-min and λ-max on the independent test set.

#### 2.3.2 Comparative analysis with baseline models

We evaluated the performance of our convolutional neural network (CNN) model for predicting the maximum and minimum values of λ against several baseline models, using a range of metrics such as Mean Squared Error (MSE), Root Mean Squared Error (RMSE), Mean Absolute Error (MAE), 95th Percentile Absolute Error (95% AE), Coefficient of Determination (R^2^), and Explained Variance Score (EVS) (Methods). As shown in Supplementary Figures 7-8, our CNN model outperformed the baseline models on most metrics, demonstrating lower MSE, RMSE, and MAE, as well as higher R² and EVS values, indicating superior overall predictive accuracy. However, the CNN model exhibited a higher 95% AE compared to SVM for the minimum value prediction and compared to both SVM and MLP for the maximum value prediction, suggesting that our model may have limitations in handling extreme errors. Despite this, the model's strong performance on the other metrics suggests that it effectively captures the main trends in the data. The higher 95% AE may reflect a prediction bias in extreme cases.

#### 2.3.3 Comparison of feature extraction outcomes using different protein representation models

The evaluation of different protein embedding features in the λ-min and λ-max prediction tasks demonstrated that the features extracted by ProtTrans-T5-XL-UniRef50, the model used in this study, consistently delivered strong performance. Specifically, ProtTrans-T5-XL-UniRef50 achieved the best results across all metrics in the λ-min prediction task and exhibited superior performance in most metrics for the λ-max task (Supplementary Tables 2-3; Supplementary Figures 9-10). This consistent performance makes ProtTrans-T5-XL-UniRef50 the preferred choice for protein feature extraction in this study.

### 2.4 λ value’s application

The results above indicate that the model can achieve satisfactory results in predicting the λ range. We select the absolute value of the median of the λ range as the recommended λ value for LinearDesign. However, upon examining the actual application of λ in LinearDesign, it is observed that when λ exceeds 20, the CAI value remains almost constant while the MFE value continues to increase. These results suggest that increasing λ beyond 20 does not lead to further optimization of the mRNA sequence. Therefore, a scaling operation is performed on the recommended λ value to ensure it remains below 20.

The module provides an optimal solution for balancing stability and protein expression and recommends a λ parameter that can be used in LinearDesign for new protein sequences. This capability aids in balancing stability and protein expression during the mRNA sequence design of the optimal protein sequence, significantly contributing to the development of tumor neoantigen mRNA vaccines.

# Supplementary Methods

## Input data

NeoDesign utilizes high-quality neoantigen peptides as its input data. Typically, neoantigen vaccines comprise 20 to 30 unique peptides (Ott *et al.*, 2020; Awad *et al.*, 2022), derived from mutations identified either through whole-exome sequencing (WES) and predictive algorithms (Garcia-Garijo *et al.*, 2019) or via mass spectrometry-based methods (Abelin *et al.*, 2017; Yadav *et al.*, 2014). These peptides form an input library, represented as a brown-colored input library in Figure 1, which serves as the basis for subsequent analyses.

## Neoantigen prediction pipeline

A neoantigen prediction pipeline has been developed, integrating various prediction methods to identify potential neoantigens at peptide junctions. Neoantigens occurring at these junctions are referred to as unexpected neoantigens (Supplementary Figure 11). The pipeline incorporates Pepsickle (Weeder *et al.*), NetChop (version 3.1) (Nielsen *et al.*, 2005), NetMHCpan (version 4.1) (Reynisson *et al.*, 2020), and MHCflurry (version 2.0) (O’Donnell *et al.*, 2020) taking into account factors such as proteasome cleavage and MHC-peptide binding affinities. It utilizes three key parameters: proteasome cleavage probability, eluted ligand rank (EL rank), and binding affinity rank (BA rank). The default thresholds are set as proteasome cleavage >0.5, EL rank <0.05, and BA rank <0.05. Users can adjust these thresholds to suit their specific needs. The selection of thresholds significantly influences prediction outcomes. Conservative settings (proteasome cleavage >0.5, EL rank <0.05, BA rank <0.05) enhance confidence in eliminating unexpected neoantigens but may increase linker usage. Conversely, using higher thresholds for EL and BA ranks (> 0.05) and lower thresholds for proteasome cleavage (< 0.5) optimizes linker design, thereby reducing linker usage.

## Greedy algorithm and decision function for Optimal Path Filtering

A greedy algorithm, a well-known method for local optimum search, is employed to develop a robust solution for the optimal path filtering challenge. The algorithm utilizes a decision function to guide the sequential addition of neoantigen peptides, selecting the most favorable peptide at each step to ultimately determine the optimal protein sequence. As neoantigen peptides are added sequentially, the pool of available peptides for subsequent steps diminishes. The application of the greedy algorithm results in the optimal path, which represents the generation of the optimal protein sequence. The decision function integrates several essential criteria for an optimal sequence, including minimal linker usage, reduced occurrence of unexpected neoantigens and functional domains, and a flexible structural configuration.

*Defined decision function = num*l+domain+looseness (1)*

The parameter 'num' plays a crucial role in minimizing linker occurrence. Optional libraries for each peptide are established, containing peptides that can be directly connected in pairs with the specific peptide without the need for linkers. The parameter num represents the count of peptides within the optional library for a specific peptide. The value of 'num' serves as a critical indicator of the peptide's flexibility during the optimal path filtering process. Specifically, a low ‘num’ value suggests that the peptide has limited flexibility, as it has fewer connectable options available. Such a peptide should be prioritized for connection to prevent a scenario where subsequent additions of peptides further reduce the number of available options, thereby constraining the flexibility of choice. Conversely, a high ‘num’ value indicates that the peptide is relatively flexible, with a greater number of connectable peptides available in its library. These peptides do not require immediate connection, allowing for a more adaptable approach in the assembly process. This flexibility ensures that the addition of linkers and the overall structure of the peptide chain can be optimized based on the available ‘num’ values, which act as a key factor in avoiding linker incorporation. The parameter 'l' balances structural considerations (domain and looseness) and minimizes linker-related factors (num). The default value for 'l' is set to 1, representing equal weighting of structural and linker conditions. A larger value prioritizes minimizing linkers, while a smaller value emphasizes structural factors. The parameter 'domain' is derived from functional domain prediction using the hmmscan submodule of the HMMER method to scan the Pfam database (Finn *et al.*, 2011). Predicting functional domains using hmmscan requires a threshold, with a recommended value of 1e-5. A lower threshold increases the reliability of the predicted functional domains but may reduce the number of domains identified. The parameter 'looseness' is a custom function utilized to evaluate the protein's structural looseness. It is based on the secondary structure prediction software GOR4, which calculates the content of α-helix, β-sheet, and coil (Garnier *et al.*, 1978). The 'looseness function' is defined as (helix + sheet - coil) %, where a lower value indicates a looser protein structure.

*Looseness = (helix + sheet - coil) % (2)*

At each step of the optimal path filtering, a smaller value of the decision function indicates a better peptide choice.

## 4. Linker library for Linker Addition

A linker library is built to add linkers where necessary. Based on established research, the linkers in the library meet the following criteria: 1) Linkers are generally 10 to 15 amino acids in length. 2) Linkers are typically composed of non-polar hydrophobic amino acids such as glycine (Gly), serine (Ser), and proline (Pro). 3) Linkers often consist of repeating units such as GGGGS, GS or GPG, and S (Xue *et al.*, 2022; Chen *et al.*, 2013). The linkers in the default linker library include"GGGGSGGGGS", "SGGGGSGGGGG", "GSGSGSGSGS", "GGSGGSGGSGGS", "GGGGSGGGGGSAAA", "GGGGSGAAAGGSGGGGG", "GSGSSGSGSS". Users can define the composition of the linker library to suit their specific needs.

## 5. λ-calculation for λ-Evaluation

LinearDesign first introduced the λ parameter to balance the free energy and the Codon Adaptation Index(CAI) (Zhang *et al.*, 2023) The specific formula is as follows (MFE: Minimum Free Energy. CAI: Codon Adaptation Index):

*MFE-*λ*CAI (3)*

When the formula reaches its minimum value, the current codon is selected as the most effective. As the algorithm iterates through each codon in the RNA sequence, the current codon is considered superior to other synonymous codons. Therefore, the MFE - λCAI value for the fixed current codon must be less than that of any synonymous codons. For each codon in the current sequence, the following inequality needs to be computed:

*MFE_1_-*λ*CAI_1_< MFE_n_-*λ*CAI_n_ (4)*

MFE_1_ and CAI_1_ represent the Minimum Free Energy and Codon Adaptation Index of the current sequence, calculated with the fixed codon present in the sequence. In contrast, MFE_n_ and CAI_n_ of the current sequence are those calculated with the presence of synonymous codons. Solving the aforementioned inequality estimates the range of values for λ. As the inequality is calculated each time when each codon in the sequence is iterated one by one, the range of possible values for λ progressively narrows (Supplementary Figure 12). After iterating through all the codons, the range of λ values is established for the current sequence.

## Data collection and λ difference analysis for λ-Evaluation

Datasets from Kaggle's COVID-19 mRNA Vaccine Degradation Datasets and a dataset from Sanofi encoding the hemagglutinin antigen for flu vaccines were collected (Rhiju Das, H Wayment-Steele, Do Soon Kim, Christian Choe, Bojan Tunguz, Walter Reade, Maggie Demkin, 2020; Li *et al.*, 2023). The mRNA sequences within these datasets are considered high-performing sequences. These datasets represent mRNA degradation and expression data, respectively, serving as the foundation for the λ-Evaluation module. λ-calculations were performed for each mRNA sequence within the datasets. The datasets were then classified based on their degradation scores into high-degscore and low-degscore mRNA groups, or based on their protein expression levels into high-expression and low-expression mRNA groups. Differential analysis of the minimum and maximum λ values was conducted across these groups. Due to the non-normal distribution of the data, the Mann-Whitney U test was employed for differential analysis.

## Model building and protein features extraction for λ-Evaluation

A prediction model employing a convolutional neural network (CNN) is built to predict the optimal ranges of λ for new protein sequences. The mRNA sequences in the collected datasets are converted to their corresponding protein sequences. The model begins with an initial 1D convolutional layer equipped with 128 filters, followed by four additional convolutional layers, each maintaining the same filter configuration and employing ReLU activation functions. After flattening the output from these convolutional layers, the data passes through four fully connected layers, each with a dropout rate of 50%. The final output layer, sized to match the dimension of λ, produces continuous values without an activation function (Supplementary Table 4). Training involves using Mean Absolute Error (MAE) as the loss function, with the dataset split into training, validation, and test sets in an 8:1:1 ratio, and employing ten-fold cross-validation. The test set is completely independent, not involved in the training and validation process, and is used solely for model performance evaluation. The model uses AdamW as the optimizer, with a learning rate of 1e-5 over 1000 epochs (Supplementary Table 5). The protein sequences are converted into embeddings representing protein features for use in the prediction model. The protein representation model ProtTransT5-XL-UniRef50 is used to extract embeddings from the protein sequences. After feature extraction, each protein sequence is transformed into a vector of dimensions (1024,1), serving as the input to our prediction model (Elnaggar *et al.*, 2022).

## Model evaluation for λ-Evaluation

- 1. **Evaluation metrics on the independent test set**

The model's performance on the independent test set is comprehensively assessed using a variety of evaluation metrics. These metrics include Mean Squared Error (MSE), Root Mean Squared Error (RMSE), Mean Absolute Error (MAE), the 95th Percentile Absolute Error (percentile_95_AE), Explained Variance Score (EVS), and the coefficient of determination, commonly referred to as R². Lower values of MSE, RMSE, MAE, and percentile_95_AE, combined with higher values of EVS and R² close to 1, indicate more optimal modeling performance. The mathematical formulations of these metrics are presented below, along with a detailed explanation of their components.

Mean Squared Error (MSE):

$MSE = \frac{1}{n}\sum_{i=1}^{n} {(y_{i}-\hat{y}_{i})}^{2}$ (5)

Root Mean Squared Error (RMSE):

$RMSE = \sqrt{\frac{1}{n}\sum_{i=1}^{n} {(y_{i}-\hat{y}_{i})}^{2}}$ (6)

MAE (Mean Absolute Error)：

$MAE=\frac{1}{n}\sum_{i=1}^{n} |y_{i}-\hat{y}_{i}|$ (7)

95th Percentile Absolute Error (percentile_95_AE)：

$Percentile\_95\_AE=\mathrm{Percentile}_{95}(|y_{1}-\hat{y}_{1}|,|y_{2}-\hat{y}_{2}|,...,|y_{n}-\hat{y}_{n}|)$ (8)

Coefficient of Determination (R²):

$R^{2}=1-\frac{\sum_{\boldsymbol{i=1}}^{\boldsymbol{n}} {\boldsymbol{(}\boldsymbol{y}_{\boldsymbol{i}}\boldsymbol{-}{\hat{\boldsymbol{y}}}_{\boldsymbol{i}}\boldsymbol{)}}^{\boldsymbol{2}}}{\sum_{\boldsymbol{i=1}}^{\boldsymbol{n}} {\boldsymbol{(}\boldsymbol{y}_{\boldsymbol{i}}\boldsymbol{-}\bar{\boldsymbol{y}}\boldsymbol{)}}^{\boldsymbol{2}}}$ **(9)**

Explained Variance Score (EVS):

$EVS=1-\frac{Var(y_{i}-\hat{y}_{i})}{Var(y_{i})}$ (10)

($y_{i}$: The actual value of the ith observation in the dataset. $\hat{y}_{i}$: The predicted value for the ith observation generated by the model. n: The total number of observations in the test set. $\left| y_{i}-\hat{y}_{i} \right|:$the absolute error for the ith observation, which is the absolute difference between the actual and predicted values. $Var(y_{i})$: the variance of the actual values in the dataset, representing the variability inherent in the data. $Var(y_{i}-\hat{y}_{i})$:The variance of the differences between the actual values and the predicted values.)

- 1. **Comparison with the baseline model**

Several baseline models, including Random Forest (RF), Support Vector Machine (SVM), Multilayer Perceptron (MLP), and Bayesian Ridge Regression, are used for comparison with the convolutional neural network (CNN) in the same λ prediction task. The dataset is divided into training, validation, and test sets with an 8:1:1 ratio, and a random seed of 13 is applied to ensure reproducibility. All models, including the proposed deep learning model and the baseline models, are trained and validated using these splits, with performance evaluated on a shared independent test set to maintain consistency in comparison. Extensive hyperparameter tuning is performed for each baseline model using grid search, systematically exploring various combinations of hyperparameters to identify the optimal configuration based on performance on the validation set. The optimal configurations for each baseline model are summarized in Supplementary Table 6. The performance of each model is assessed using several key metrics, including Mean Squared Error (MSE), Root Mean Squared Error (RMSE), Mean Absolute Error (MAE), 95th Percentile Absolute Error (percentile_95_AE), Explained Variance Score (EVS), and the Coefficient of Determination (R²). Each model is evaluated using this diverse set of metrics on the independent test set.

**8.3 Comparison of feature extraction methods using different protein representation models**

The performance of extracting protein features using various protein representation models is compared with that of the ProtT5-XL-UniRef50 model, which is adopted in this study. ProtTrans_T5_XL_BFD, ESM-1b (ESM1b_t33_650M_UR50S), the ESM2 series, and ProtBERT/ProtBERT_BFD are included in the comparison. The embeddings representing protein features extracted by each model are used in the same downstream λ prediction task. The parameters of all the protein representation models used in this comparison, including model architecture, parameter counts, and training datasets, are presented in Supplementary Table 7. The effectiveness of each model's feature extraction is assessed using multiple evaluation metrics, including Mean Squared Error (MSE), Root Mean Squared Error (RMSE), Mean Absolute Error (MAE), 95th Percentile Absolute Error (percentile_95_AE), Explained Variance Score (EVS), and the Coefficient of Determination (R²).

## Data preparation for NeoDesign implementation

### 9.1 Target protein preparation

100 patient samples are retrieved from The Cancer Genome Atlas (TCGA) public database, specifically selecting neoantigen peptides derived from single nucleotide variants (SNVs) in pancreatic and lung cancers. To maintain practicality, the number of peptides per patient is limited to between 10 and 30. In the neoantigens prediction pipeline, the proteasome cleavage probability threshold is set at 0.5, and thresholds of 0.05 are set for both EL rank and BA rank. For the decision function, the balancing parameter 'l' is set to 1 and a recommended threshold of 1e-5 is used for predicting the number of functional domains with HMMER. After configuring these threshold settings, the 100 samples are processed by NeoDesign, resulting in the generation of 100 target proteins. These proteins represent the optimal neoantigen vaccine sequences designed by NeoDesign for each patient. Detailed information on the optimal protein sequences generated can be found in the supplementary data available on the website.

### 9.2 Reference proteins preparation

100 well-known functional proteins are selected randomly from the Protein Data Bank (PDB), including Ubiquitin-protein ligase E3A and DNA damage-binding protein. These reference proteins serve as a benchmark to assess the functional domains and looseness of our predicted optimal vaccine sequences. Detailed information on the reference proteins can be found in the supplementary data available on the website.

## Results evaluation for NeoDesign implementation

### 10.1 Evaluation of NeoDesign's optimal protein sequences

The linker numbers, peptide numbers, domain numbers, and looseness values are calculated for the 100 target proteins. Additionally, domain numbers and looseness values are calculated for the 100 reference proteins to enable a comparative analysis with the target proteins

### 10.2 Evaluation of NeoDesign's recommended λ

The mRNA sequences are optimized using LinearDesign's default λ parameter for 100 target sequences. The values of Minimum Free Energy (MFE) and Codon Adaptation Index (CAI) under the default parameter are obtained and termed “default MFE” and “default CAI”. The mRNA sequences are also optimized using the recommended λ parameters provided by NeoDesign for 100 target sequences. The values of MFE and CAI under the recommended parameter are obtained and termed “recommended MFE” and “recommended CAI”. MFE and CAI relative difference ratios are defined to measure the relative change in MFE and CAI using the recommended λ parameter values compared to the default parameter values.

$MFE Relative Difference Ratio=\frac{Recommended MFE - Default MFE}{Default MFE}$ (11)

$CAI Relative Difference Ratio=\frac{Recommended CAI - Default CAI}{Default CAI}$(12)

The gain function is an evaluation metric that assesses the relative improvement of the recommended λ parameter compared to the default λ parameter. A gain function value greater than zero indicates that the recommended parameter is more effective, while a value less than zero that the default parameter is superior.

$Gain=MFE Relative Difference Ratio+CAI Relative Ratio$ （13）

### 10.3 Evaluation of NeoDesign’s performance

NeoDesign is evaluated on two servers with different configurations to assess its universality. Server 1 is equipped with a 4-core Intel Xeon Gold 6133 CPU running at 2.50GHz, featuring a moderate-sized L3 cache of 28,160 KB. This server is suitable for a range of tasks and efficiently handles moderate workloads. Server 2 features a more powerful 40-core Intel Xeon CPU E5-2630 v4 with a clock speed of 2.20 GHz and an L3 cache of 25,600 KB. Due to its high core count and multi-threading capability, Server 2 excels at handling demanding workloads that require extensive parallel processing. Server 2 represents the performance of NeoDesign under optimal conditions, while Server 1 represents average conditions. As a test, 20 neoantigen peptides as input data are processed by NeoDesign, and the time and resources consumed on the different servers are recorded. NeoDesign is compared to the existing tool pVACvector in terms of results and running time. 100 patient samples retrieved from The Cancer Genome Atlas (TCGA) public database are processed by pVACvector, generating 100 comparison sequences. The parameters used in pVACvector, such as the selection of neoantigen prediction methods and the internal methods of the NeoDesign neoantigen prediction pipeline are consistent, including MHCflurry, NetMHCpan, and NetMHCpanEL. In addition, the prediction thresholds, such as the binding threshold and percentile threshold, are aligned with those of NeoDesign to ensure the validity of the comparison. A comparison of linker numbers is conducted between the 100 comparison sequences generated by pVACvector and the 100 target sequences generated by NeoDesign, and the difference in the number of linkers generated by the two tools is analyzed.

# Supplementary Discussion

## Discussion on the selection of tools in the NeoDesign pipeline

The selection of computational tools in the NeoDesign pipeline was guided by empirical performance evaluations, comprehensive literature reviews, and considerations of computational efficiency to ensure robust and reproducible results in neoantigen prediction. For pMHC binding prediction, the latest versions of NetMHCpan 4.1 and MHCflurry 2.0 were chosen based on a thorough evaluation by Maria Bonsack et al., which assessed the performance of 13 widely used neoantigen prediction algorithms using an independent MHC affinity dataset (Bonsack *et al.*, 2019). For protein cleavage prediction, Pepsickle and NetChop 3.1 were selected for their demonstrated accuracy in predicting proteasomal cleavage sites, as supported by a detailed comparative study of multiple cleavage prediction tools (Weeder *et al.*). For protein secondary structure prediction, although SSPro6 has been identified in several studies as a top-performing method, we currently employ the GOR4 method (Urban *et al.*, 2022; Torrisi *et al.*, 2019). To validate this choice, we conducted a comparative analysis of GOR4 and SSPro6, focusing on coil content and looseness across both reference and target protein datasets. The results indicated a high degree of similarity in coil content predictions between the two methods, with only minor discrepancies in looseness, particularly within the target protein dataset. Despite these differences, the overall distribution trends for looseness were consistent between GOR4 and SSPro6 (Supplementary Figures 13A-D). Given that SSPro6 is more resource-intensive and considering that secondary structure prediction is only one part of our broader workflow, these minor differences were deemed not to significantly impact the overall outcomes. Therefore, we decided to continue using GOR4 for secondary structure prediction. For functional domain prediction, we primarily employed the Pfam database after comparing it with other databases such as CDD, SMART, PROSITE, and Superfamily on reference proteins (Mistry *et al.*, 2021; Lu *et al.*, 2020; Lei *et al.*, 2022; Hulo, 2006; Gough, 2002). Our analysis demonstrated that Pfam predicted a higher number of domains with a more balanced overall distribution compared to other databases (Supplementary Figure 13E), leading to its selection as the primary tool for functional domain prediction in our pipeline. Methods such as dPUC and CODD (Terrapon *et al.*, 2009; Ochoa *et al.*, 2011), which refine Pfam predictions, were not considered, as our focus was on identifying critical domains rather than refining existing predictions. Although integrating multiple databases could theoretically increase the number of predicted domains and reduce the risk of omission, it could also introduce redundancies and errors. Therefore, we opted not to use a union-based approach that aggregates predictions from multiple tools. Based on our comparative analysis, Pfam offered the best combination of performance and reliability, making it the primary tool in the NeoDesign pipeline for functional domain prediction. All specific detailed methodological comparison data can be found in the Supplementary Tables online (GitHub repository of Huanglab-Fudan).

## Discussion on the differential performance of protein language models in the λ-value prediction task

In this study, we systematically evaluated the performance of protein features extracted by various protein representation models in predicting λ-min and λ-max. The models we examined include ProtTransT5-XL-UniRef50, ProtTransT5-XL-BFD, ESM-1b, multiple variants of the ESM2 series, and ProtBERT-UniRef100/ProtBERT-BFD. These models differ significantly in terms of architecture, parameter size, and training data—factors that profoundly impact their performance in downstream tasks. Firstly, our analysis revealed that models with larger parameter sizes generally perform better, but excessively increasing parameter sizes can lead to diminishing returns. This finding highlights the importance of selecting a model's parameter size based on the specific needs of the task to balance complexity and performance effectiveness. Secondly, models trained on the UniRef50 or UniRef100 datasets, such as ProtTransT5-XL-UniRef50 and ProtBERT-UniRef100, consistently outperformed those trained on the broader BFD dataset, such as ProtTransT5-XL-BFD and ProtBERT-BFD, underscoring the significance of high-quality, specific training data. Finally, the ESM models demonstrated robust and adaptable performance across tasks, while the ProtTrans models excelled in specific areas but showed uneven overall results. In contrast, the ProtBERT models underperformed, suggesting the superiority of Transformer architectures over BERT for processing one-dimensional protein sequences. These insights underscore the critical role of both architecture and training data in optimizing model performance for specific tasks and provide guidance for selecting appropriate protein representation models to extract protein features from sequences.

# Supplementary Figures


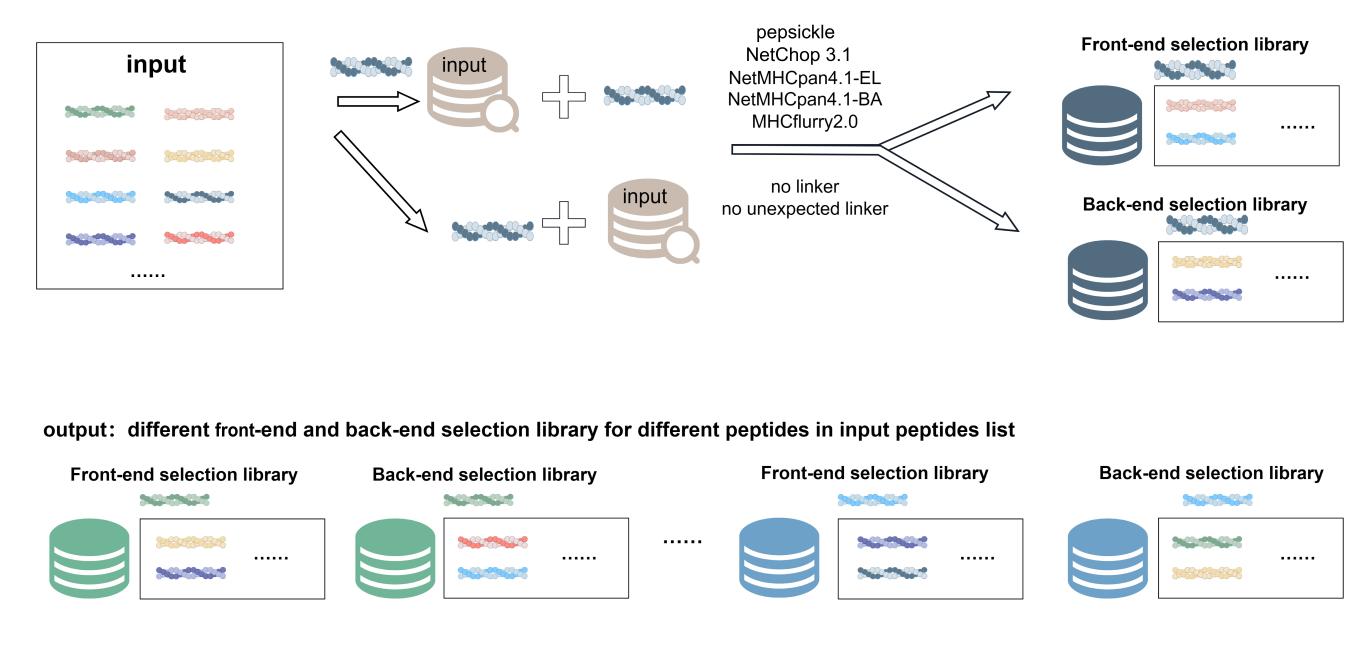


**Supplementary Figure 1. Pipeline of Library Construction.** At the top of the figure, one of the peptides (blue) is used as an example. The colors of the libraries and peptides are matched, indicating that each peptide generates a corresponding optional library during the library construction process. The outcome includes front-end optional libraries and back-end optional libraries for all peptides. (By Figdraw)


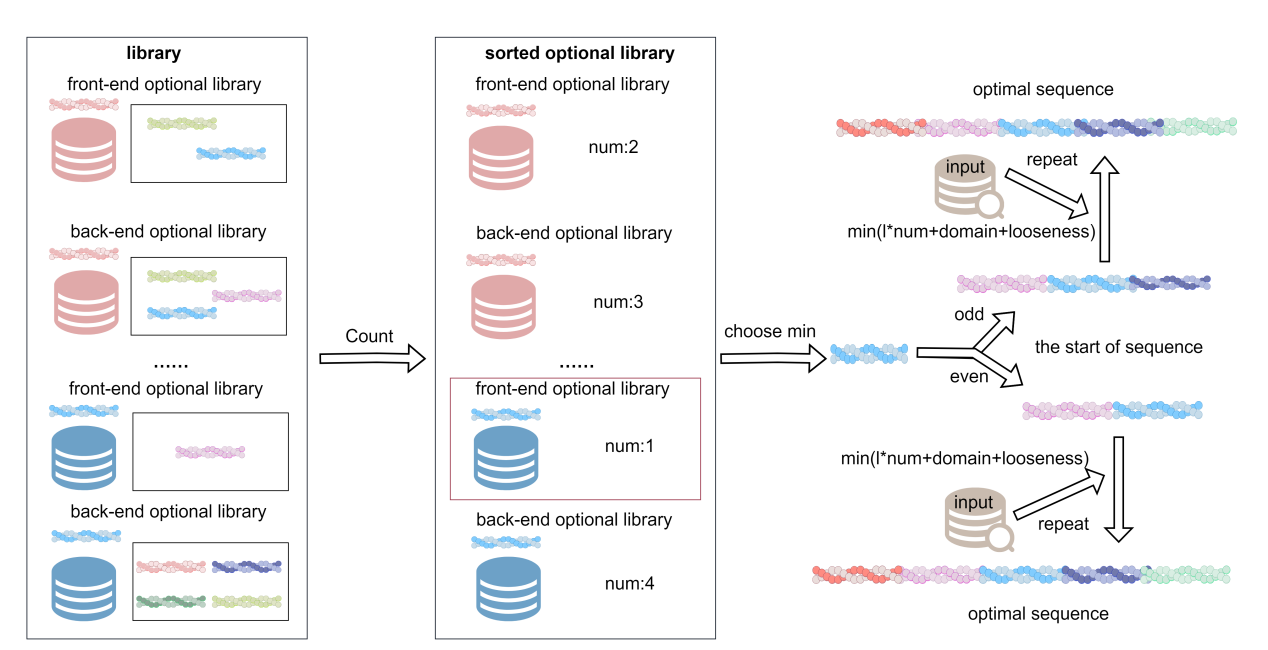


**Supplementary Figure 2. Pipeline of Optimal Path Filtering.** This module can be primarily divided into three parts. The first part involves the statistical sorting of the optional libraries. The second part is the establishment of the sequence starting point. The peptide sequence with the least number of optional peptides in its corresponding library is selected as the starting point. The third part involves a greedy approach that prioritizes peptides with minimal decision function(*l*num+domain+looseness*) values in the library. These peptides are added and placed in subsequent steps according to the constructed objective function. This process leads to the attainment of an optimal sequence. (By Figdraw)


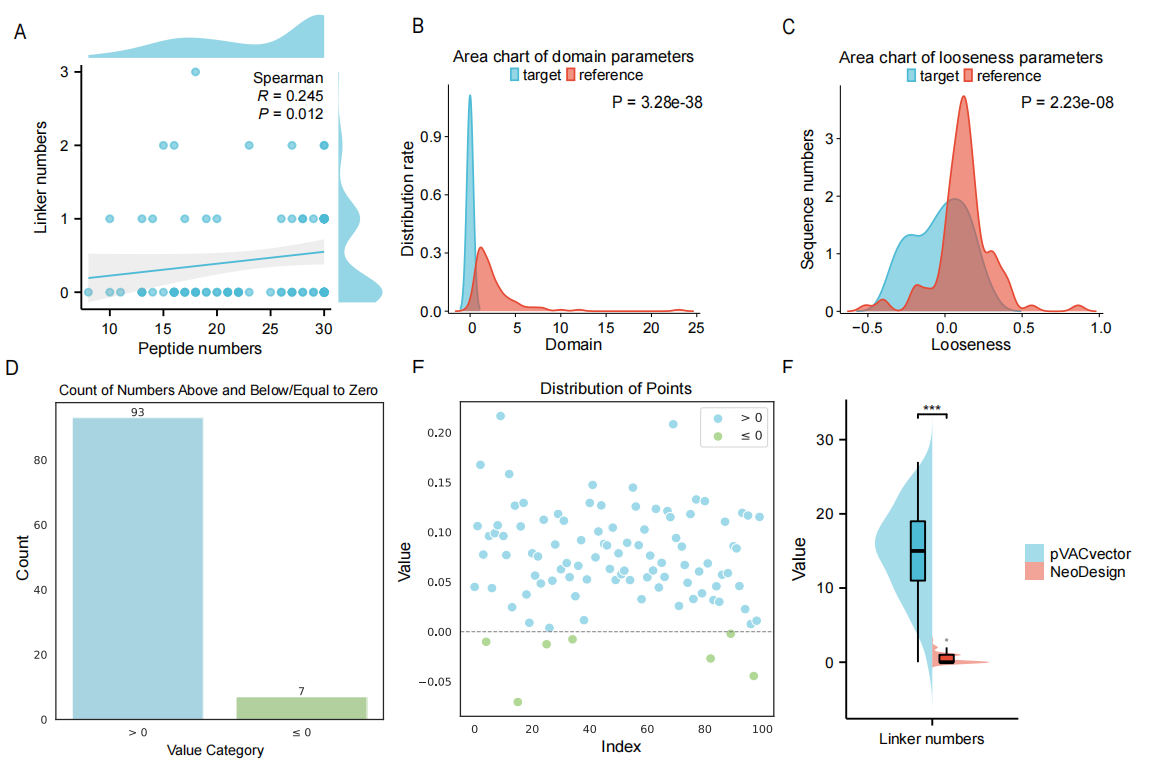


**Supplementary Figure 3. The implementation and performance of NeoDesign.** (A) Distribution plot of linker numbers and peptide numbers. The upper part of the figure represents the distribution of peptide numbers, while the right part represents the distribution of linker numbers. The blue line depicts the correlation between linker numbers and peptide segment numbers. (B) Area chart of domain parameters. The distribution rate represents the percentage of sequences within each domain number relative to the total number of sequences. The blue markers represent the protein sequences designed by us, while the red markers represent known functional reference proteins. (C) Area chart of looseness parameters. The sequence number is the count of sequences corresponding to each looseness value. (D) Count of values above and below/equal to zero for a specific value category. The value refers to the gain function of 100 target proteins. (E) The data distribution of gain function. Index refers to the ordinal number of 100 target proteins. (F) Comparison plot of linker numbers generated by different tools.


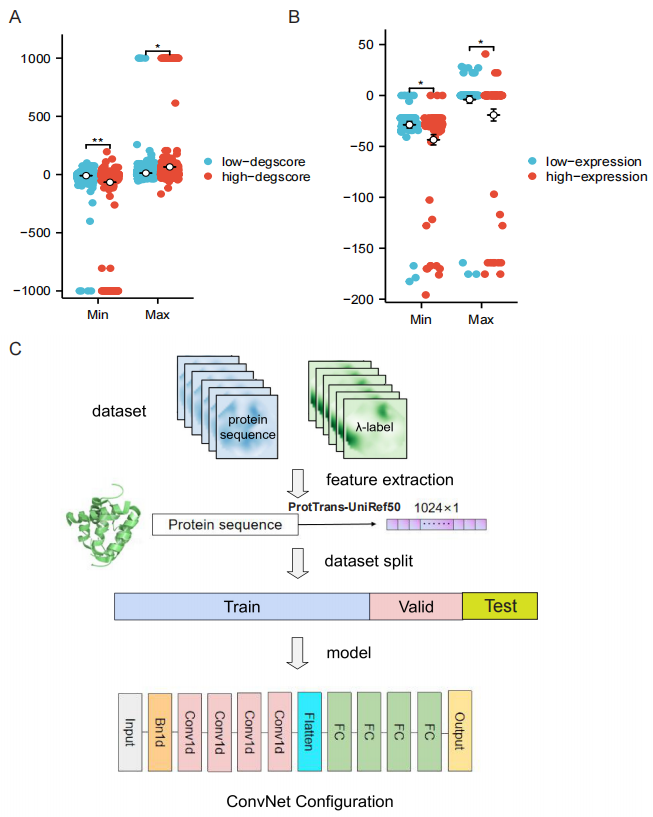


**Supplementary Figure 4. Difference analysis of λ and the framework of the prediction model in λ-Evaluation.** (A) A comparison plot of the maximum and minimum λ values in the mRNA degradation dataset. (B) A comparison plot of the maximum and minimum λ values in the mRNA expression dataset. (C) The framework of the prediction model in λ-Evaluation. The model inputs are protein sequences and the λ labels calculated through λ-calculation. These inputs undergo feature extraction using the protein representation models for proteins, ProtTrans-T5-XL-UniRef50, which is followed by data division into training, validation, and test sets for model training. The model is composed of several layers: a convolutional layer (Conv1d), normalization layers (Bn1d, BatchNorm1d), a flattening layer (Flatten), and fully connected layers (Full-Connect, FC). ConvNet: Convolutional Network.


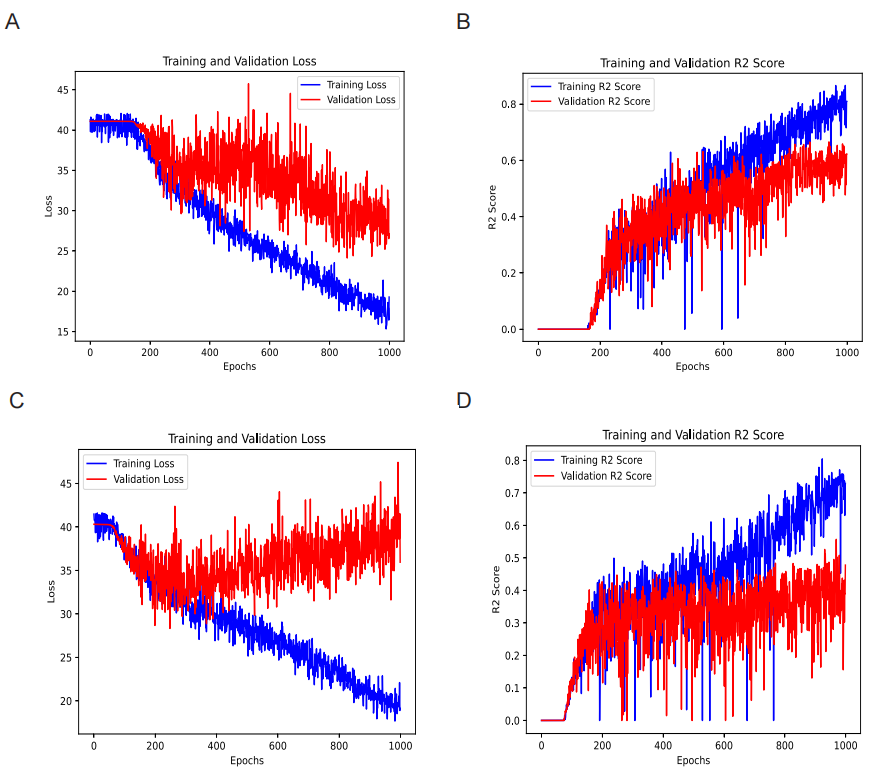


**Supplementary Figure 5. Evaluation of model performance.** (A) Training and validation loss over 1000 epochs. for predicting the minimum values of λ. (B) Training and validation R^2^ score over 1000 epochs. for predicting the minimum values of λ. (C) Training and validation loss over for predicting the maximum values of λ. (D) Training and validation R^2^ score over 1000 epochs for predicting the maximum values of λ. (An epoch refers to a single iteration of training where the model processes the entire dataset. Loss refers to Mean Absolute Error (MAE)).


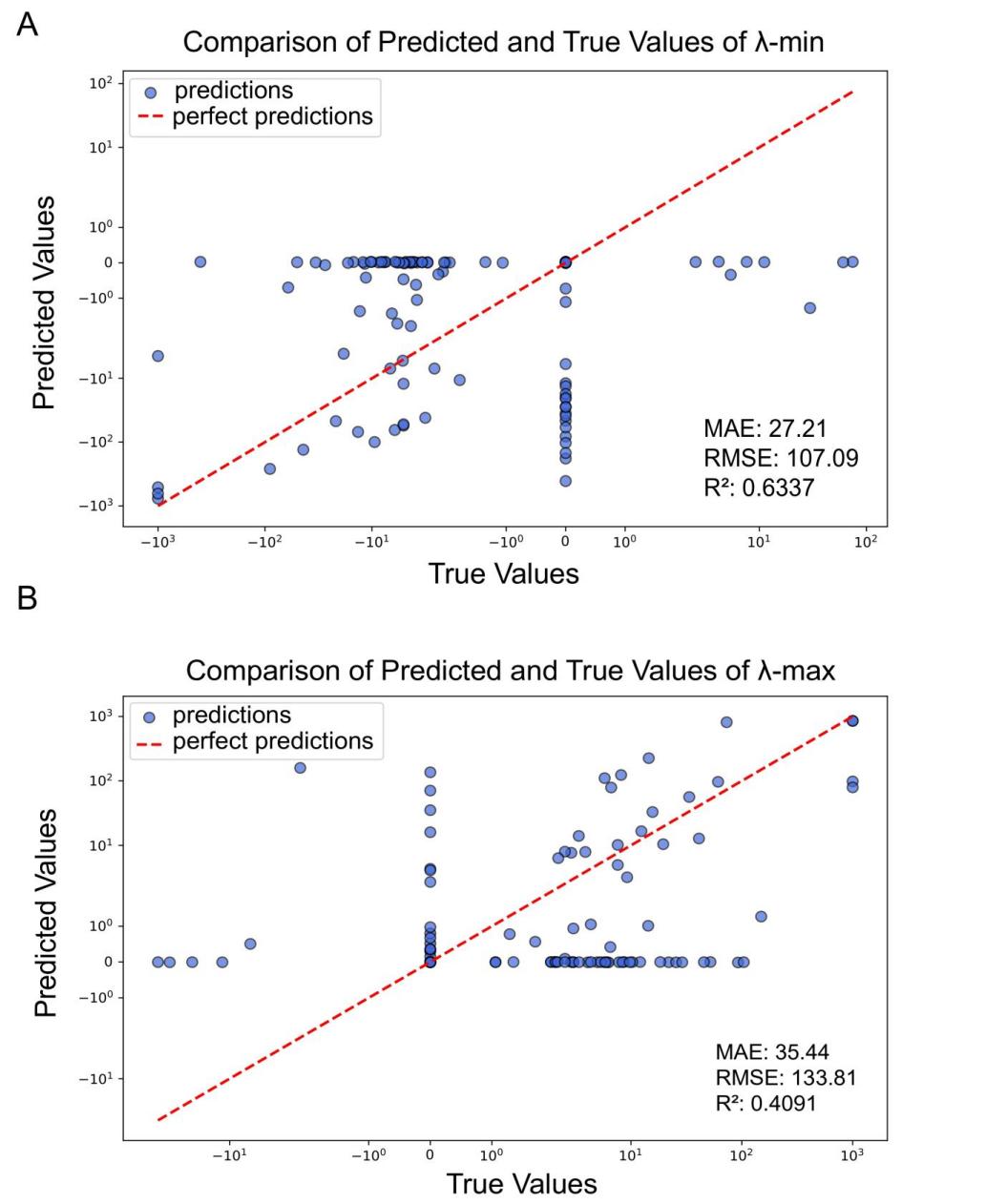


**Supplementary Figure 6. Performance of the convolutional neural network model on the independent test set.** (A) Performance of predicted and true values of λ-min. (B) Performance of predicted and true values of λ-max (RMSE: Root Mean Squared Error; MAE: Mean Absolute Error).


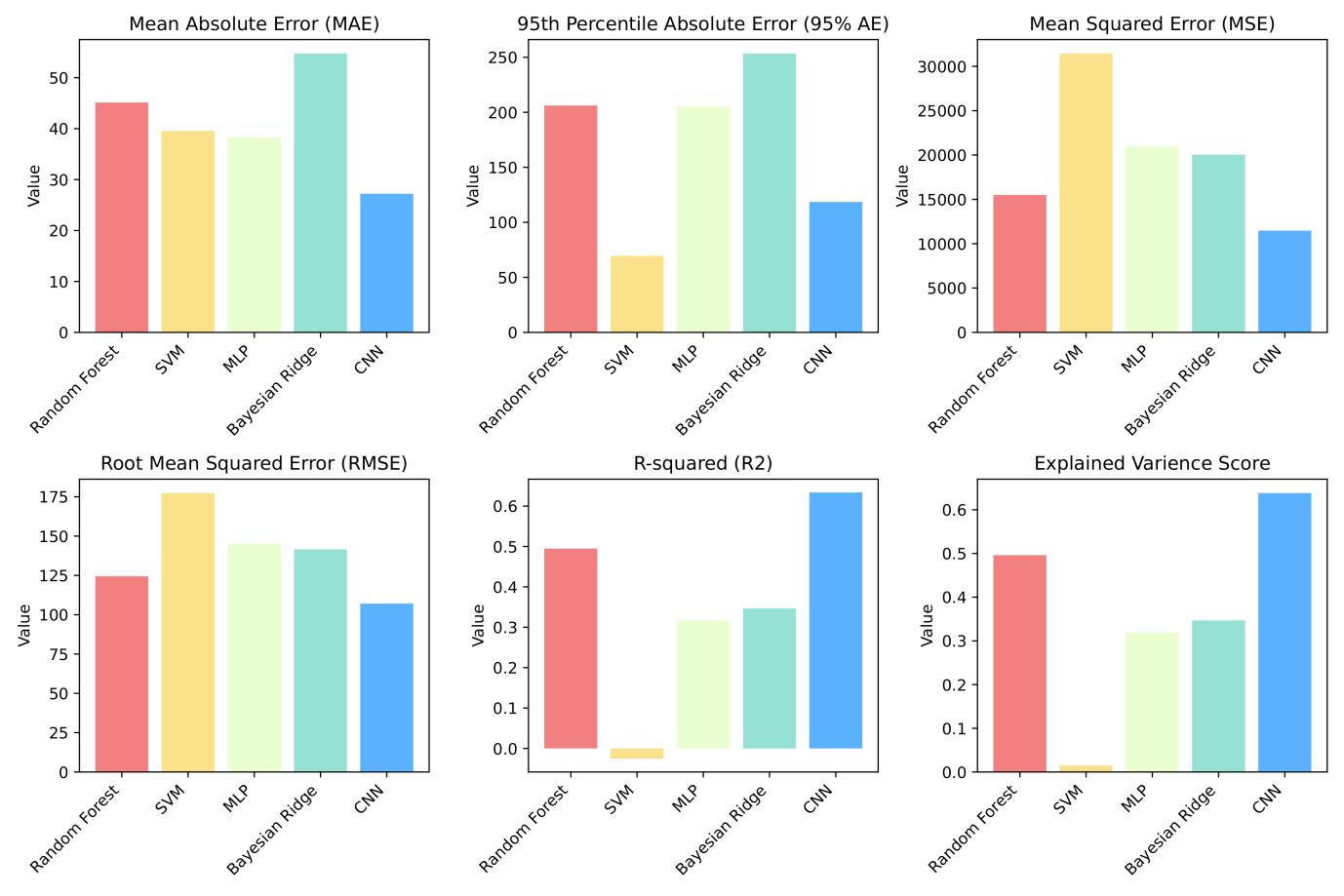


**Supplementary Figure 7. Evaluation of λ-min convolutional neural network model and baseline models.**


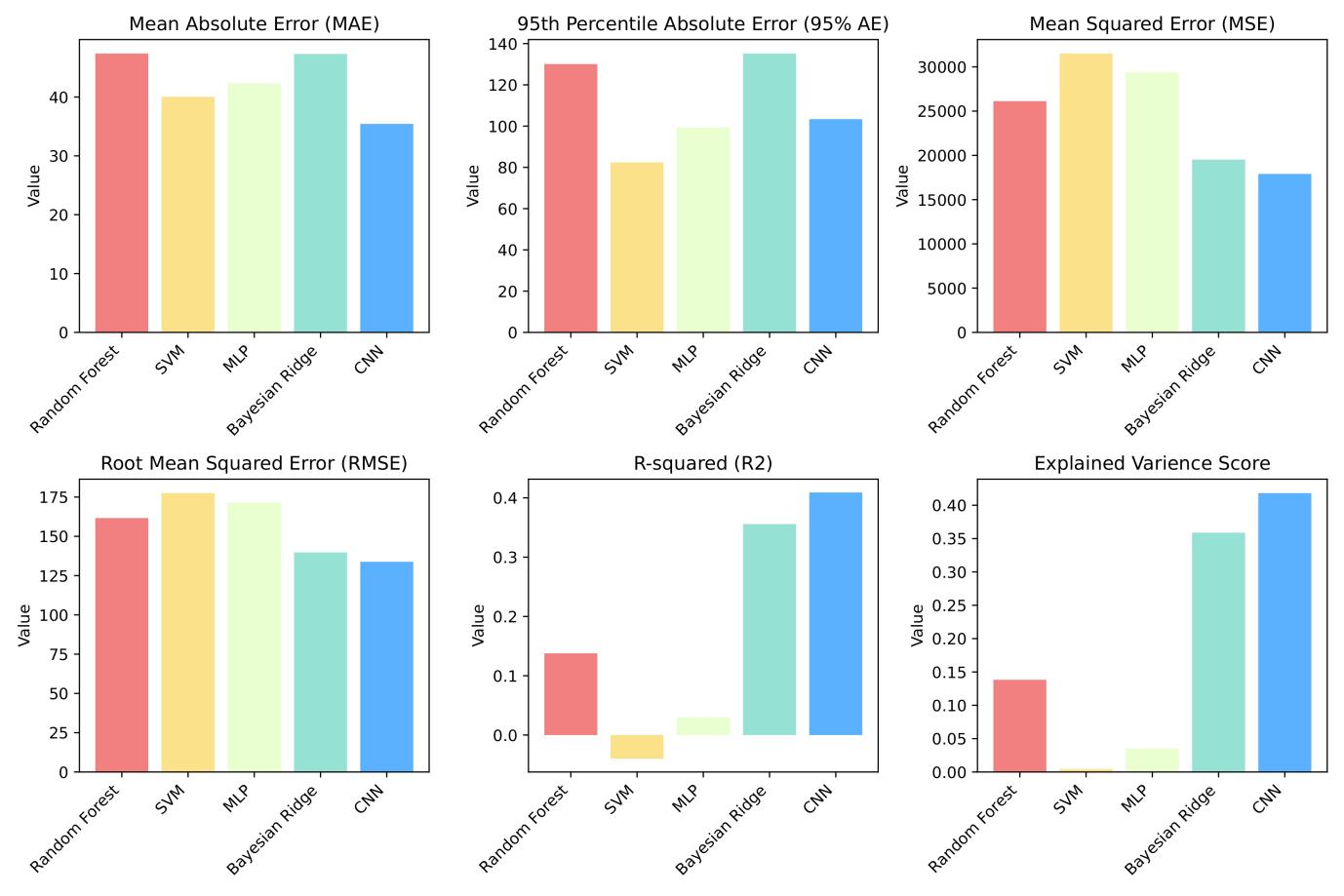
**Supplementary Figure 8. Evaluation of λ-max convolutional neural network model and baseline models.**


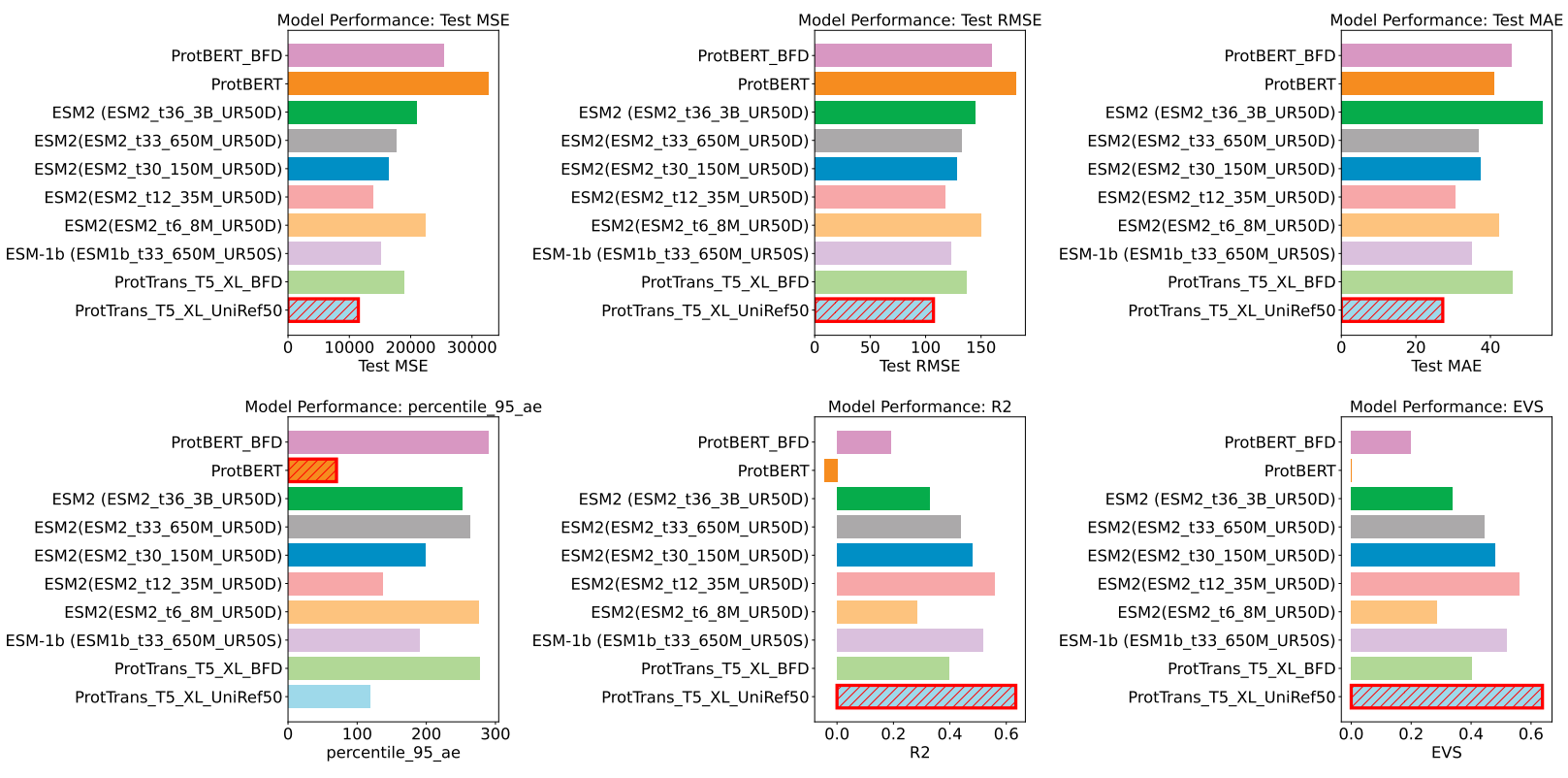


**Supplementary Figure 9. Performance of different protein representation models on the λ-min convolutional neural network model.** The horizontal bars represent the performance of various protein representation models across different evaluation metrics. The bars highlighted with a red edge indicate the best-performing model for each respective metric.

**
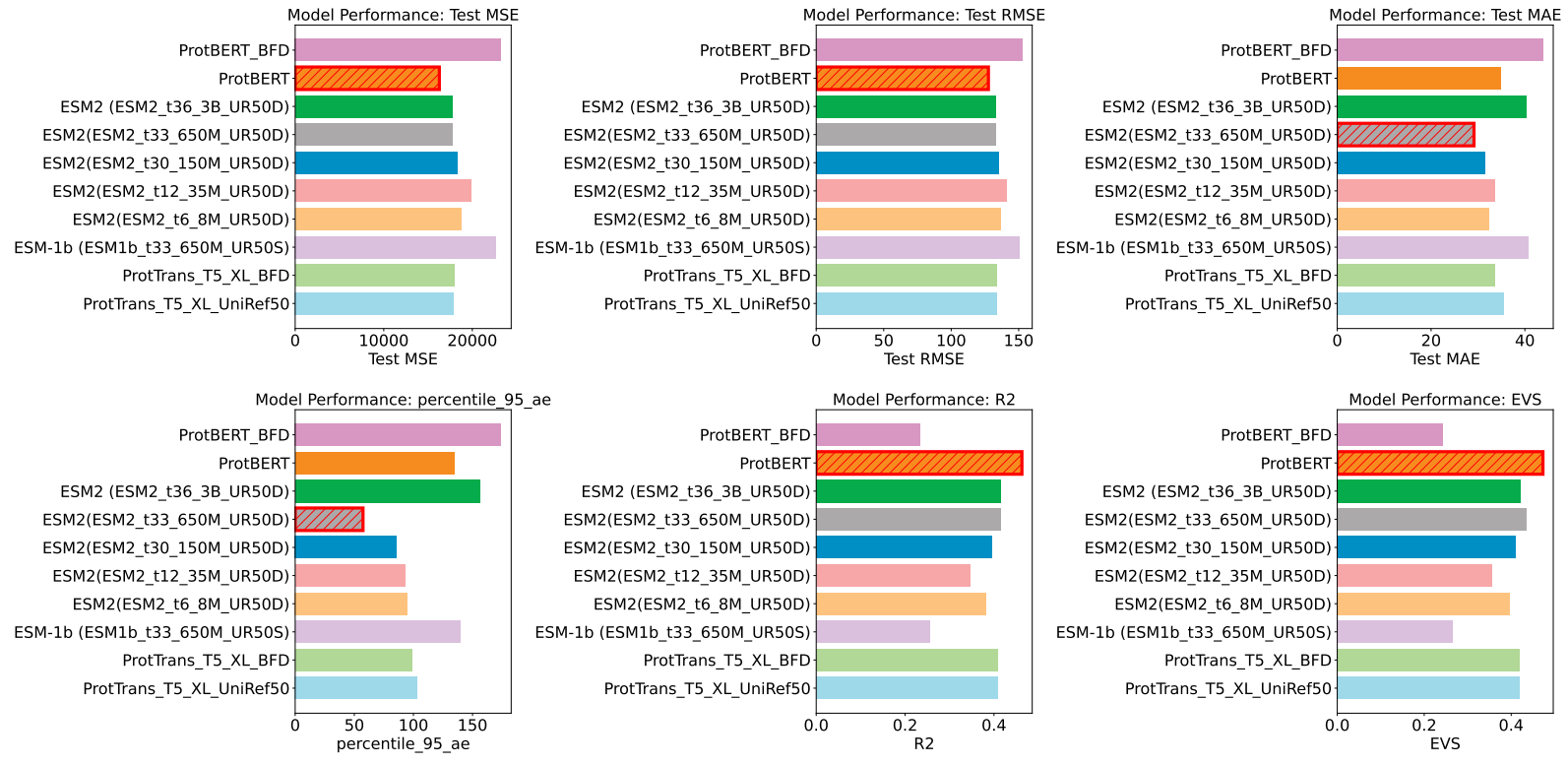
**

**Supplementary Figure 10. Performance of different protein representation models on the λ-max convolutional neural network model.** The horizontal bars represent the performance of various protein representation models across different evaluation metrics. The bars highlighted with a red edge indicate the best-performing model for each respective metric.


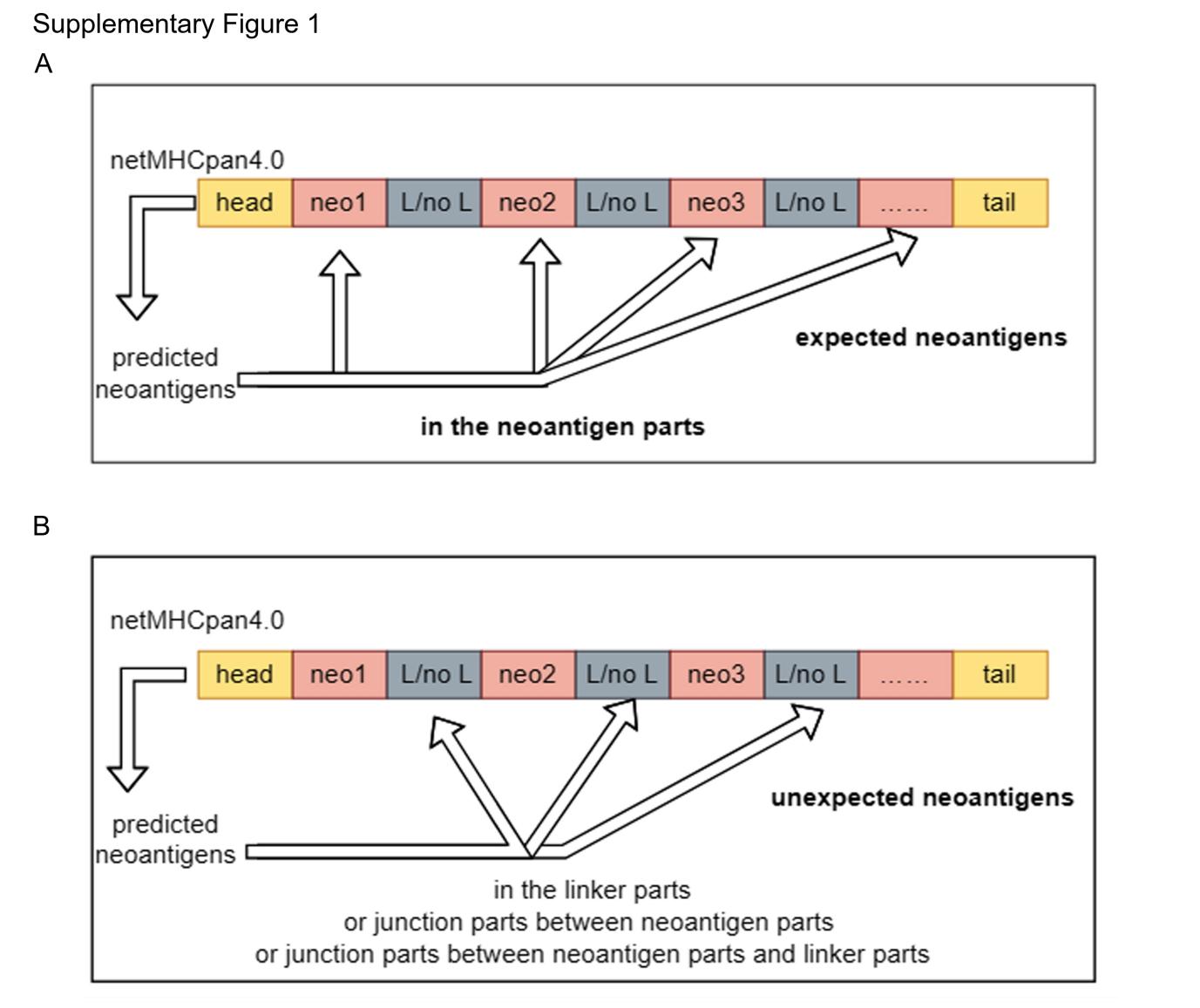


**Supplementary Figure 11. Definition of expected and unexpected neoantigens** (A) Expected neoantigens: Predicted neoantigens that are located within the neoantigen regions. (B) Unexpected neoantigens: Predicted neoantigens that are located in the linker regions or at the junctions between neoantigen regions and linker regions.


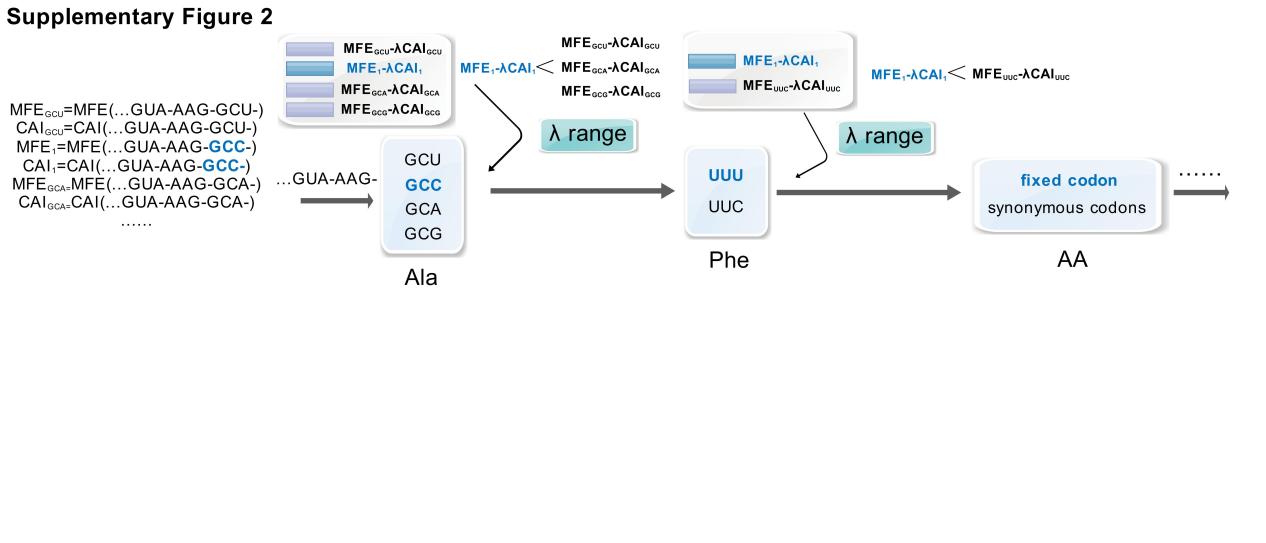


**Supplementary Figure 12.** λ**-calculation.** A λ range is calculated by solving the inequality MFE_1_-λCAI_1_< MFE_n_-λCAI_n_ during each iteration of the codons in the RNA sequence. The Minimum Free Energy (MFE_1_) and Codon Adaptation Index (CAI_1_) of the current sequence are calculated with the fixed codon (blue). Conversely, the MFE_n_ and CAI_n_ of the current sequence are calculated with synonymous codons (e.g., MFE_GCU_, CAI_GCU_). "AA" refers to amino acids.


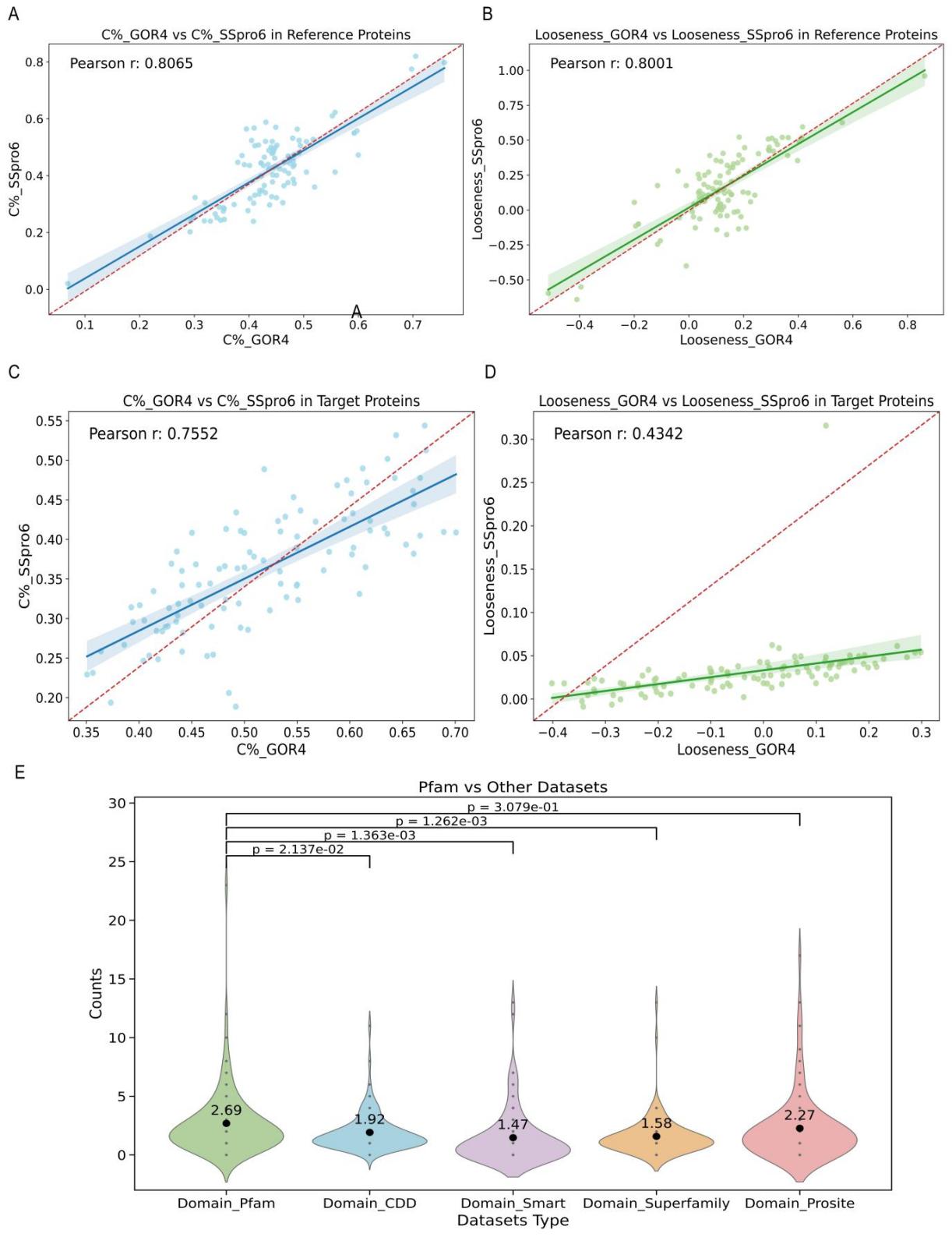


**Supplementary Figure 13. Comparison of protein secondary structure prediction software and domain prediction datasets.** (A) Correlation between C% values predicted by GOR4 and SSpro6 for reference proteins. (B) Correlation between looseness values predicted by GOR4 and SSpro6 for reference proteins. (C) Correlation between C% values predicted by GOR4 and SSpro6 for target proteins. (D) Correlation between looseness values predicted by GOR4 and SSpro6 for target proteins. (E)Violin plots comparing the distribution of domain counts across different datasets, with statistical significance indicated by p-values. C% refers to coil content in the sequence. Looseness = (helix + sheet - coil) %.

**Supplementary Tables**

| **Supplementary Table 1**: Performance of different metrics on the independent test set | | |
| --- | --- | --- |
| Metrics | λ-min | λ-max |
| Mean Absolute Error (MAE) | 27.21 | 35.44 |
| 95th percentile Absolute Error (95% AE) | 118.55 | 103.36 |
| Mean Squared Error (MSE) | 11468.81 | 17905.80 |
| Root Mean Squared Error (RMSE) | 107.09 | 133.81 |
| R-squared | 0.63 | 0.41 |
| Explained Variance Score | 0.64 | 0.42 |

| **Supplementary Table 2:** Performance of different protein representation models on the λ-min neural network model | | | | | | |
| --- | --- | --- | --- | --- | --- | --- |
| Model Name | Test MAE | Test MSE | Test RMSE | R² | EVS | 95th Percentile AE |
| **ProtTrans-T5-XL-UniRef50(The model adopted in our study)** | **27.21** | **11468.81** | **107.09** | **0.63** | **0.64** | 118.55 |
| ProtTrans-T5-XL-BFD | 45.97 | 18827.88 | 137.21 | 0.40 | 0.40 | 277.56 |
| ESM-1b (ESM1b-t33-650M-UR50S) | 34.93 | 15155.17 | 123.11 | 0.52 | 0.52 | 189.47 |
| ESM2(ESM2-t6-8M-UR50D) | 42.27 | 22452.12 | 149.84 | 0.28 | 0.29 | 276.15 |
| ESM2(ESM2-t12-35M-UR50D) | 30.49 | 13857.31 | 117.72 | 0.56 | 0.56 | 136.10 |
| ESM2(ESM2-t30-150M-UR50D | 37.39 | 16305.65 | 127.69 | 0.48 | 0.48 | 198.56 |
| ESM-2(ESM2-t33-650M-UR50D) | 36.74 | 17615.07 | 132.72 | 0.44 | 0.44 | 262.94 |
| ESM-2(ESM2-t36-3B-UR50D) | 53.83 | 21013.72 | 144.96 | 0.33 | 0.34 | 251.57 |
| ProtBERT | 41.02 | 32725.92 | 180.90 | -0.04 | -0.00 | **70.10** |
| ProtBERT-BFD | 45.70 | 25325.79 | 159.14 | 0.19 | 0.20 | 290.23 |

| **Supplementary Table 3:** Performance of different protein representation models on the λ-min neural network model | | | | | | |
| --- | --- | --- | --- | --- | --- | --- |
| **Model Name** | **Test MAE** | **Test MSE** | **Test RMSE** | **R²** | **EVS** | **95th Percentile AE** |
| **ProtTrans-T5-XL-UniRef50(The model adopted in our study)** | 35.44 | 17905.80 | 133.81 | 0.41 | 0.42 | 103.36 |
| ProtTran-T5-XL-BFD | 33.58 | 17948.03 | 133.97 | 0.41 | 0.42 | 98.46 |
| ESM-1b(ESM1b-t33-650M-UR50S) | 40.73 | 22583.16 | 150.28 | 0.25 | 0.27 | 139.31 |
| ESM2(ESM2-t6-8M-UR50D) | 32.36 | 18706.10 | 136.77 | 0.38 | 0.40 | 95.07 |
| ESM2(ESM2-t12-35M-UR50D) | 33.68 | 19819.67 | 140.78 | 0.35 | 0.36 | 92.82 |
| ESM2(ESM2-t30-150M-UR50D | 31.53 | 18333.37 | 135.4 | 0.39 | 0.41 | 85.80 |
| **ESM-2(ESM2-t33-650M-UR50D)** | **29.15** | 17763.57 | 133.28 | 0.41 | 0.43 | **57.37** |
| ESM-2 (ESM2-t36-3B-UR50D) | 40.19 | 17740.11 | 133.19 | 0.41 | 0.42 | 156.3 |
| **ProtBERT** | 34.96 | **16276.33** | **127.58** | **0.46** | **0.47** | 134.29 |
| ProtBERT-BFD | 43.85 | 23226.40 | 152.40 | 0.23 | 0.24 | 173.99 |

| **Supplementary Table 4:** λ-Evaluation model configuration | |
| --- | --- |
| Model Configuration | Description |
| Input layer | Accepts input features, 128 filters, kernel size 1, stride 1, no padding; Batch Normalization; ReLU activation |
| Convolutional Layers 1-4 | Each has 128 filters, kernel size 1, stride 1, no padding; Batch Normalization; ReLU activation |
| Flatten Layer | Flattens the output of the convolutional layers |
| Fully Connected Layers 1-4 | Each layer has an input/output size of 128; ReLU activation; 50% Dropout |
| Output Layer | Input size 128, output size 1, no activation function |

| **Supplementary Table 5:** Model training settings | |
| --- | --- |
| Training Settings | Description |
| Loss Function | Mean Absolute Error (MAE) |
| Dataset Splitting | Training set 8：validation set 1：test set 1 |
| Validation | Ten-fold Cross-validation |
| Optimizer | AdamW |
| Learning Rate | 1e-5 |
| Epochs | 1000 |
| Batch Size | 128 |
| Random seed | 13 |

| **Supplementary Table 6**: Baseline model settings | |
| --- | --- |
| Model Name | Parameters |
| Random Forest Model | max_depth = 10, min_samples_split = 10, n_estimators = 300 |
| Support Vector Machine (SVM) Model | C = 1, gamma = 'scale', kernel = 'rbf |
| Multilayer Perceptron (MLP) Model | hidden_layer_sizes = (50, 50, 50), max_iter = 500, alpha = 0.0001 |
| Bayesian Ridge Regression Model | alpha_1 = 1e-06, lambda_1 = 0.0001 |

| **Supplementary Table 7:** Detailed parameters of different protein representation models | | | |
| --- | --- | --- | --- |
| Model Name | Main Model Architecture | Model Parameters | Training Datasets |
| ProtTrans-T5-XL-UniRef50(The model adopted in our study) | T5 Transformer | 3B | UniRef50 |
| ProtTran-T5-XL-BFD | T5 Transformer | 3B | BFD |
| ESM-1b (ESM1b-t33-650M-UR50S) | Transformer | 650M | UniRef50 + MSA |
| ESM2(ESM2-t6-8M-UR50D) | Transformer | 8M | UniRef50 |
| ESM2(ESM2-t12-35M-UR50D) | Transformer | 35M | UniRef50 |
| ESM2(ESM2-t30-150M-UR50D | Transformer | 150M | UniRef50 |
| ESM-2 (ESM2-t33-650M-UR50D) | Transformer | 650M | UniRef50 |
| ESM-2 (ESM2-t36-3B-UR50D) | T  Transformer | 3B | UniRef50 |
| ProtBERT | Bert | 110M | UniRef100 |
| ProtBERT-BFD | Bert | 110M | BFD |

**References**

Abelin, J.G. *et al.* (2017) Mass Spectrometry Profiling of HLA-Associated Peptidomes in Mono-allelic Cells Enables More Accurate Epitope Prediction. *Immunity*, **46**, 315–326.

Awad, M.M. *et al.* (2022) Personalized neoantigen vaccine NEO-PV-01 with chemotherapy and anti-PD-1 as first-line treatment for non-squamous non-small cell lung cancer. *Cancer Cell*, **40**, 1010-1026.e11.

Bonsack, M. *et al.* (2019) Performance Evaluation of MHC Class-I Binding Prediction Tools Based on an Experimentally Validated MHC-Peptide Binding Data Set. *Cancer Immunol Res*, **7**, 719–736.

Chen, X. *et al.* (2013) Fusion protein linkers: property, design and functionality. *Adv Drug Deliv Rev*, **65**, 1357–1369.

Elnaggar, A. *et al.* (2022) ProtTrans: Toward Understanding the Language of Life Through Self-Supervised Learning. *IEEE Trans Pattern Anal Mach Intell*, **44**, 7112–7127.

Finn, R.D. *et al.* (2011) HMMER web server: interactive sequence similarity searching. *Nucleic Acids Res*, **39**, W29-37.

Garcia-Garijo, A. *et al.* (2019) Determinants for Neoantigen Identification. *Front. Immunol.*, **10**, 1392.

Garnier, J. *et al.* (1978) Analysis of the accuracy and implications of simple methods for predicting the secondary structure of globular proteins. *J Mol Biol*, **120**, 97–120.

Gough, J. (2002) The SUPERFAMILY database in structural genomics. *Acta Crystallogr D Biol Crystallogr*, **58**, 1897–1900.

Hulo, N. (2006) The PROSITE database. *Nucleic Acids Research*, **34**, D227–D230.

Lei, B. *et al.* (2022) SMART v1.0: A Database for Small Molecules with Functional Implications in Plants. *Interdiscip Sci*, **14**, 279–283.

Li, S. *et al.* (2023) CodonBERT: Large Language Models for mRNA design and optimization.

Lu, S. *et al.* (2020) CDD/SPARCLE: the conserved domain database in 2020. *Nucleic Acids Res*, **48**, D265–D268.

Mistry, J. *et al.* (2021) Pfam: The protein families database in 2021. *Nucleic Acids Res*, **49**, D412–D419.

Nielsen, M. *et al.* (2005) The role of the proteasome in generating cytotoxic T-cell epitopes: insights obtained from improved predictions of proteasomal cleavage. *Immunogenetics*, **57**, 33–41.

Ochoa, A. *et al.* (2011) Using context to improve protein domain identification. *BMC Bioinformatics*, **12**, 90.

O’Donnell, T.J. *et al.* (2020) MHCflurry 2.0: Improved Pan-Allele Prediction of MHC Class I-Presented Peptides by Incorporating Antigen Processing. *Cell Syst*, **11**, 42-48.e7.

Ott, P.A. *et al.* (2020) A Phase Ib Trial of Personalized Neoantigen Therapy Plus Anti-PD-1 in Patients with Advanced Melanoma, Non-small Cell Lung Cancer, or Bladder Cancer. *Cell*, **183**, 347-362.e24.

Reynisson, B. *et al.* (2020) NetMHCpan-4.1 and NetMHCIIpan-4.0: improved predictions of MHC antigen presentation by concurrent motif deconvolution and integration of MS MHC eluted ligand data. *Nucleic Acids Res*, **48**, W449–W454.

Rhiju Das, H Wayment-Steele, Do Soon Kim, Christian Choe, Bojan Tunguz, Walter Reade, Maggie Demkin (2020) OpenVaccine: COVID-19 mRNA Vaccine Degradation Prediction. Kaggle.

Terrapon, N. *et al.* (2009) Detection of new protein domains using co-occurrence: application to *Plasmodium falciparum*. *Bioinformatics*, **25**, 3077–3083.

Torrisi, M. *et al.* (2019) Deeper Profiles and Cascaded Recurrent and Convolutional Neural Networks for state-of-the-art Protein Secondary Structure Prediction. *Sci Rep*, **9**, 12374.

Urban, G. *et al.* (2022) SSpro/ACCpro 6: almost perfect prediction of protein secondary structure and relative solvent accessibility using profiles, deep learning, and structural similarity. *Bioinformatics*, **38**, 2064–2065.

Weeder, B.R. *et al.* pepsickle rapidly and accurately predicts proteasomal cleavage sites for improved neoantigen identification.

Xue, D. *et al.* (2022) A tumor-specific pro-IL-12 activates preexisting cytotoxic T cells to control established tumors. *Sci Immunol*, **7**, eabi6899.

Yadav, M. *et al.* (2014) Predicting immunogenic tumor mutations by combining mass spectrometry and exome sequencing. *Nature*, **515**, 572–576.

Zhang, H. *et al.* (2023) Algorithm for Optimized mRNA Design Improves Stability and Immunogenicity. *Nature*.
