# Supplementary material for: NeoDesign: a computational tool for optimal selection of polyvalent neoantigen combinations
Source: Bioinformatics. 2024 Sep 27;40(10):btae585. doi: 10.1093/bioinformatics/btae585 (PMC11471261; doi:10.1093/bioinformatics/btae585)
Supplement: btae585_Supplementary_Data [file btae585_supplementary_data.zip › Supplementary Figure 10.pdf]

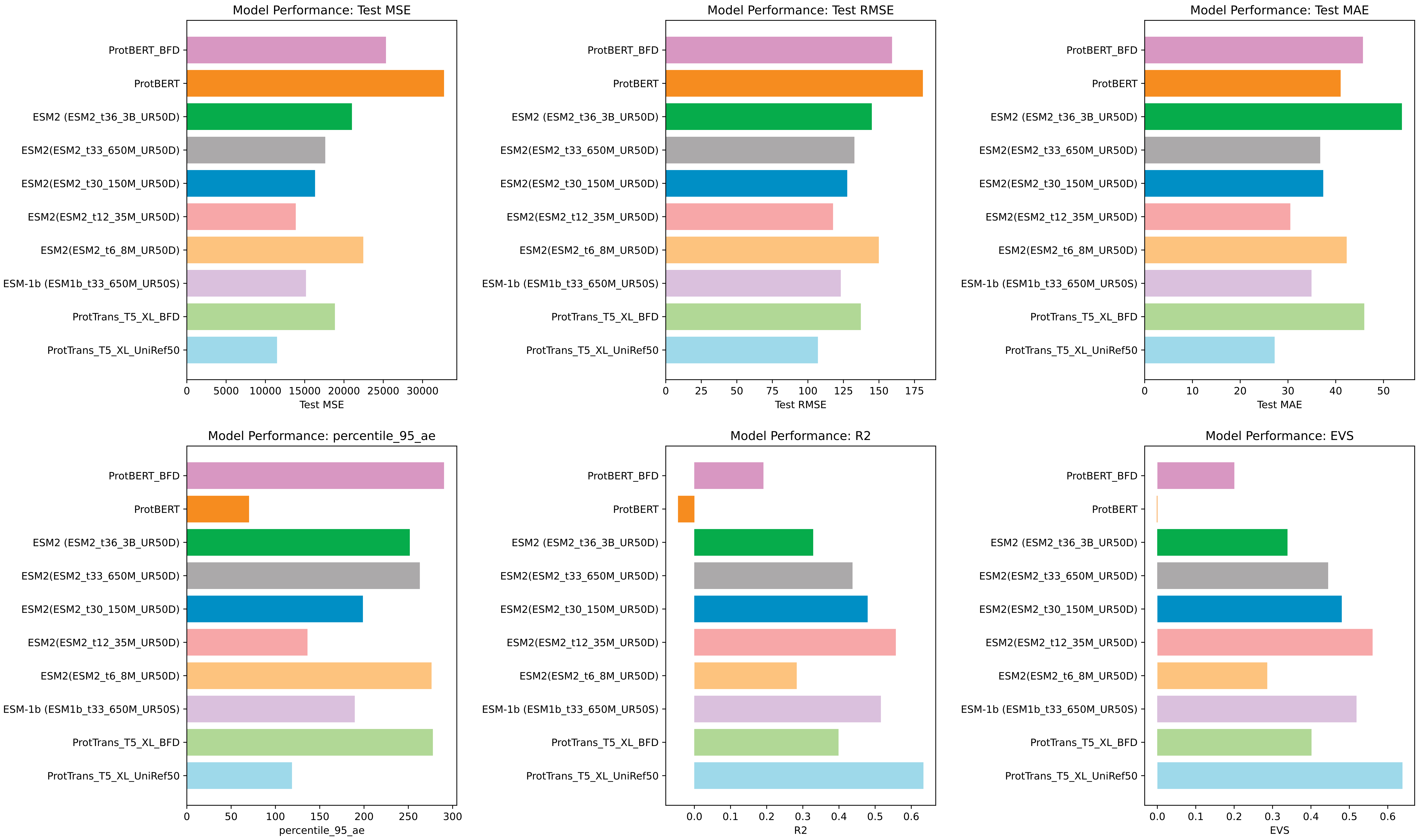

Model Performance: Test MSE
Model Performance: Test RMSE
Model Performance: Test MAE
Model Performance: percentile_95_ae
Model Performance: R2
Model Performance: EVS
ProtBERT_BFD
ProtBERT
ESM2 (ESM2_t36_3B_UR50D)
ESM2(ESM2_t33_650M_UR50D)
ESM2(ESM2_t30_150M_UR50D)
ESM2(ESM2_t12_35M_UR50D)
ESM2(ESM2_t6_8M_UR50D)
ESM-1b (ESM1b_t33_650M_UR50S)
ProtTrans_T5_XL_BFD
ProtTrans_T5_XL_UniRef50
Test MSE
Test RMSE
Test MAE
percentile_95_ae
R2
EVS
