# Supplementary material for: NeoDesign: a computational tool for optimal selection of polyvalent neoantigen combinations
Source: Bioinformatics. 2024 Sep 27;40(10):btae585. doi: 10.1093/bioinformatics/btae585 (PMC11471261; doi:10.1093/bioinformatics/btae585)
Supplement: btae585_Supplementary_Data [file btae585_supplementary_data.zip › Supplementary Figure 8.pdf]

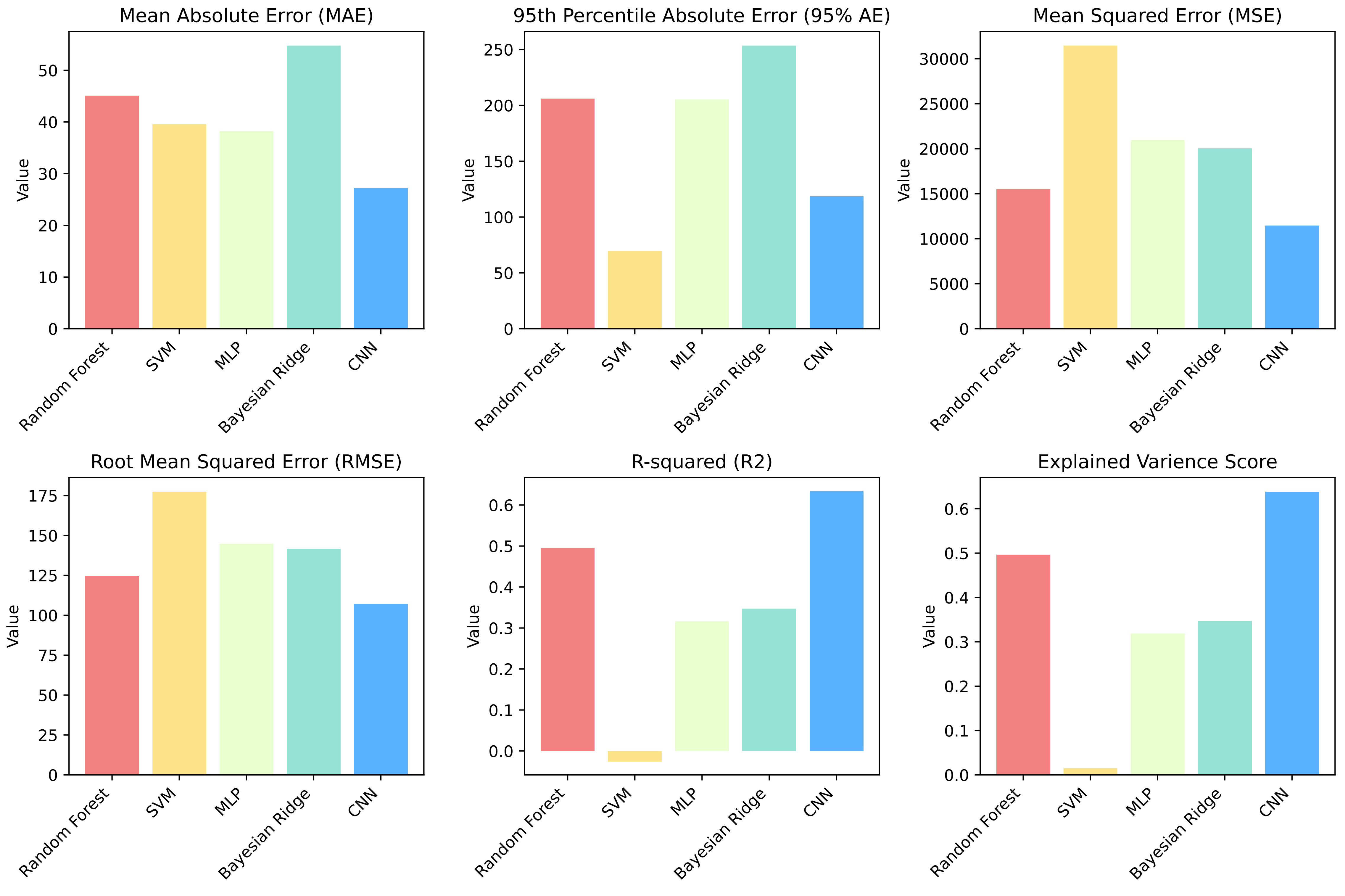
Mean Absolute Error (MAE)
Value
0
10
20
30
40
50
Random Forest
SVM
MLP
Bayesian Ridge
CNN
95th Percentile Absolute Error (95% AE)
Value
0
50
100
150
200
250
Random Forest
SVM
MLP
Bayesian Ridge
CNN
Mean Squared Error (MSE)
Value
0
5000
10000
15000
20000
25000
30000
Random Forest
SVM
MLP
Bayesian Ridge
CNN
Root Mean Squared Error (RMSE)
Value
0
25
50
75
100
125
150
175
Random Forest
SVM
MLP
Bayesian Ridge
CNN
R-squared (R2)
Value
0.0
0.1
0.2
0.3
0.4
0.5
0.6
Random Forest
SVM
MLP
Bayesian Ridge
CNN
Explained Varience Score
Value
0.0
0.1
0.2
0.3
0.4
0.5
0.6
Random Forest
SVM
MLP
Bayesian Ridge
CNN
