# Supplementary figures and images for: NeoDesign: a computational tool for optimal selection of polyvalent neoantigen combinations
Source: Bioinformatics. 2024 Sep 27;40(10):btae585. doi: 10.1093/bioinformatics/btae585 (PMC11471261; doi:10.1093/bioinformatics/btae585)

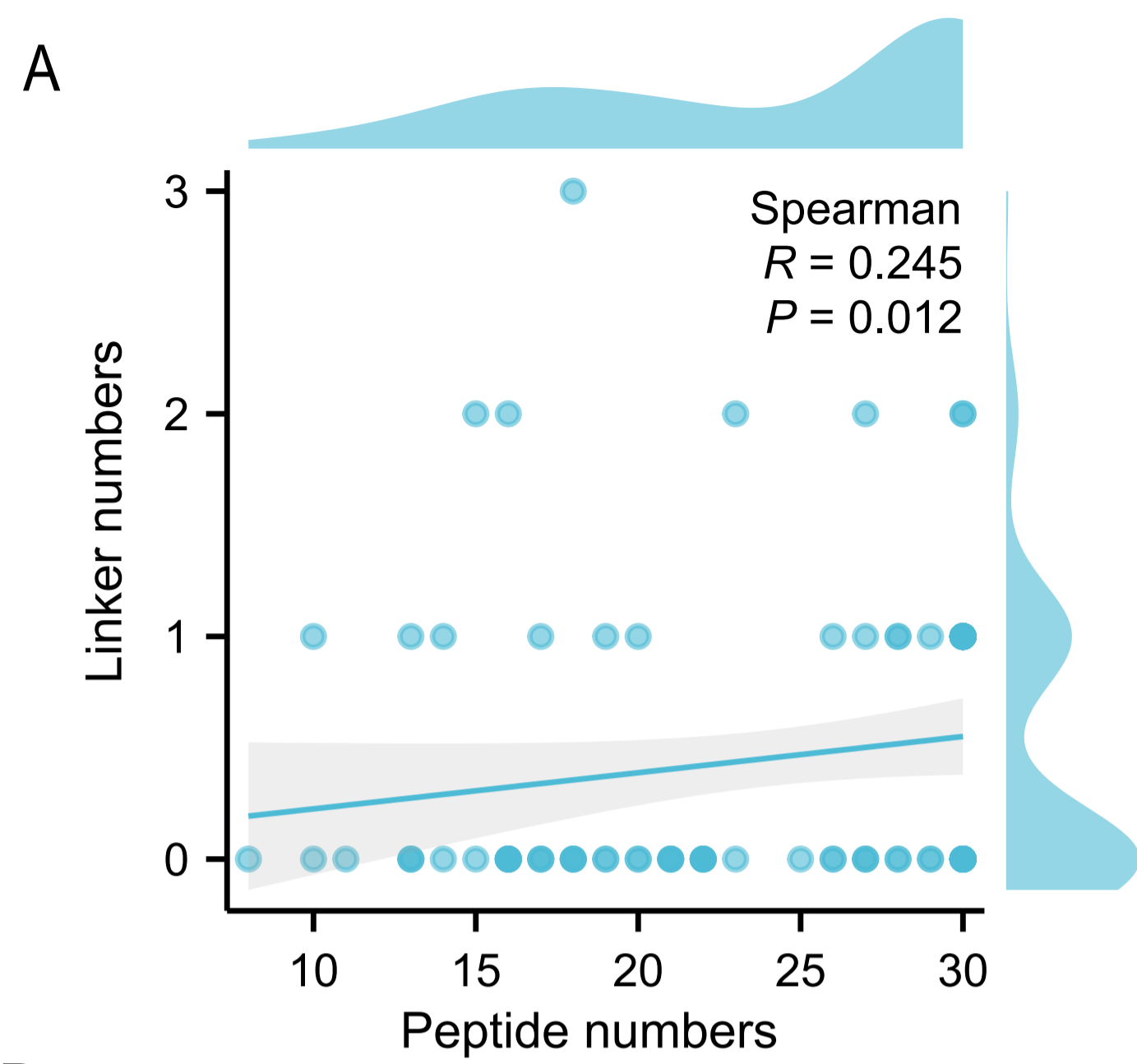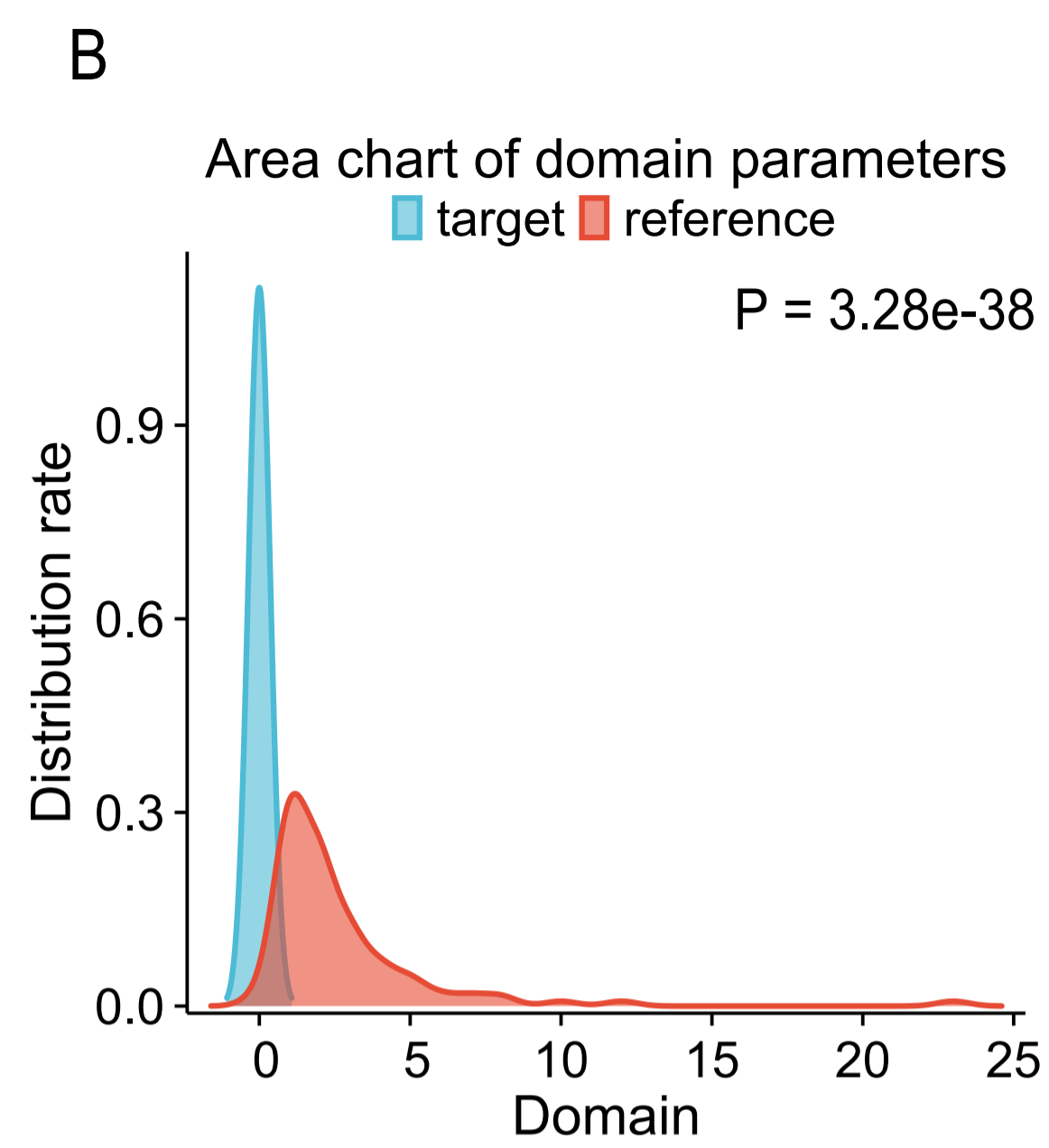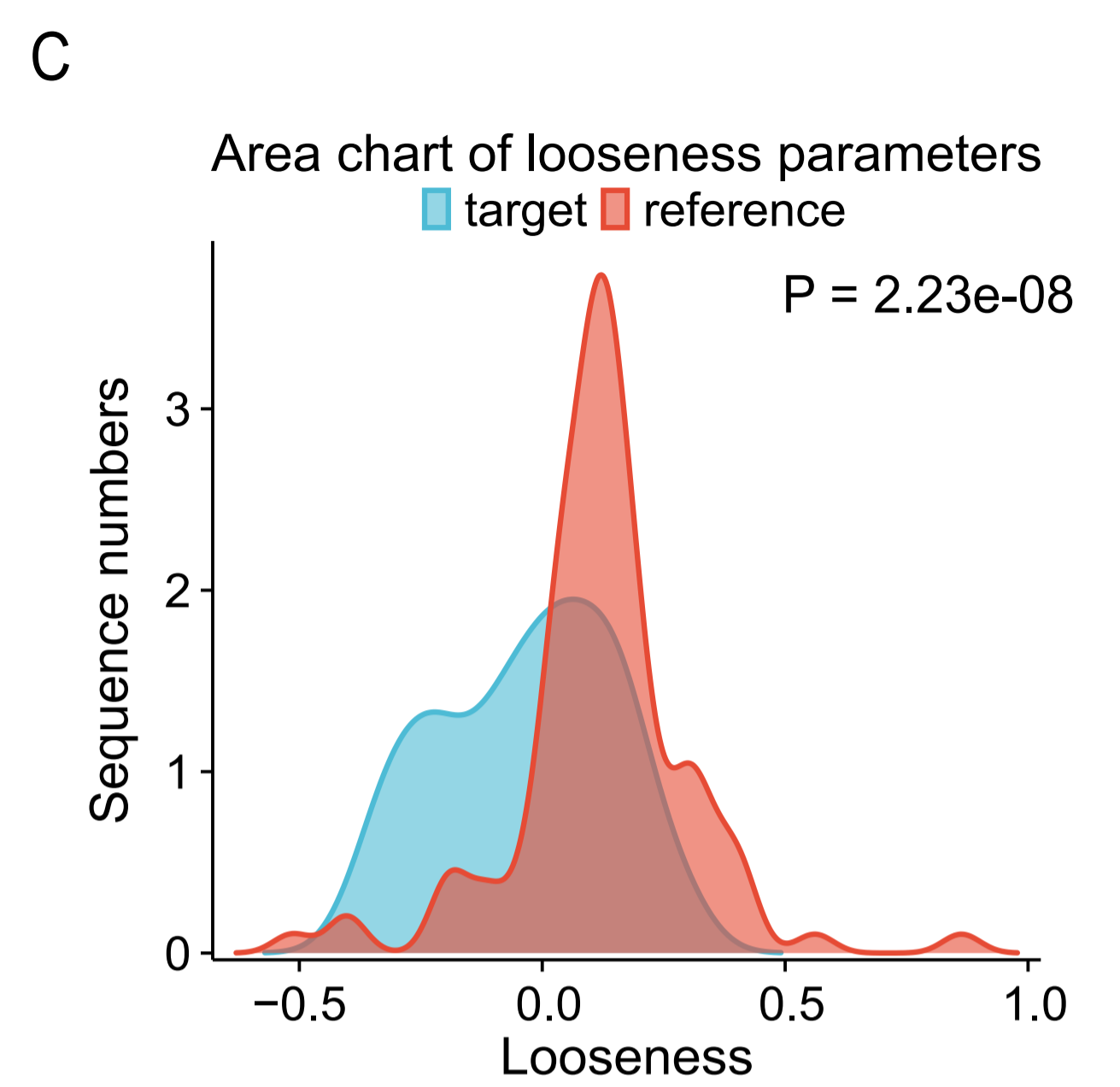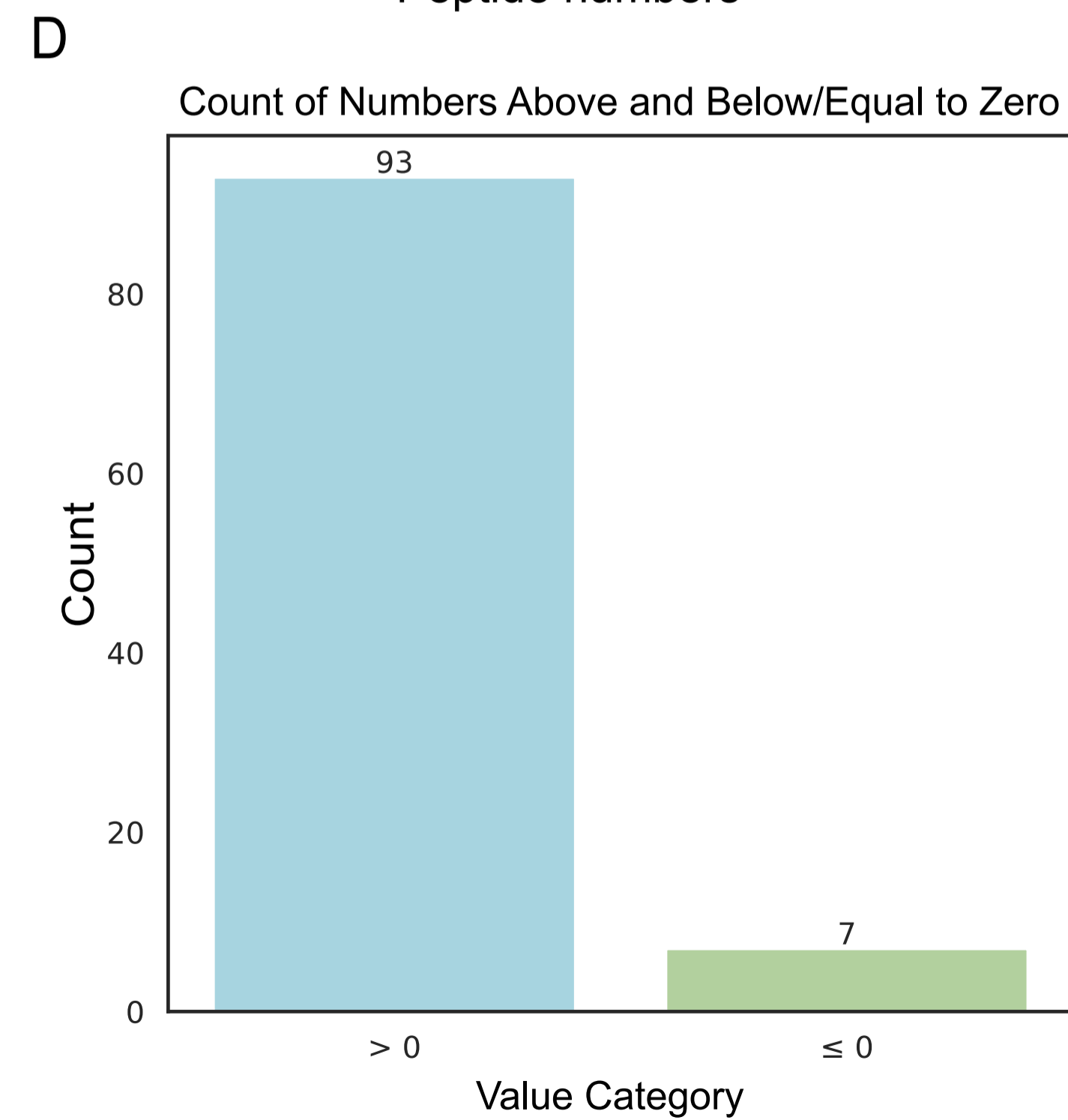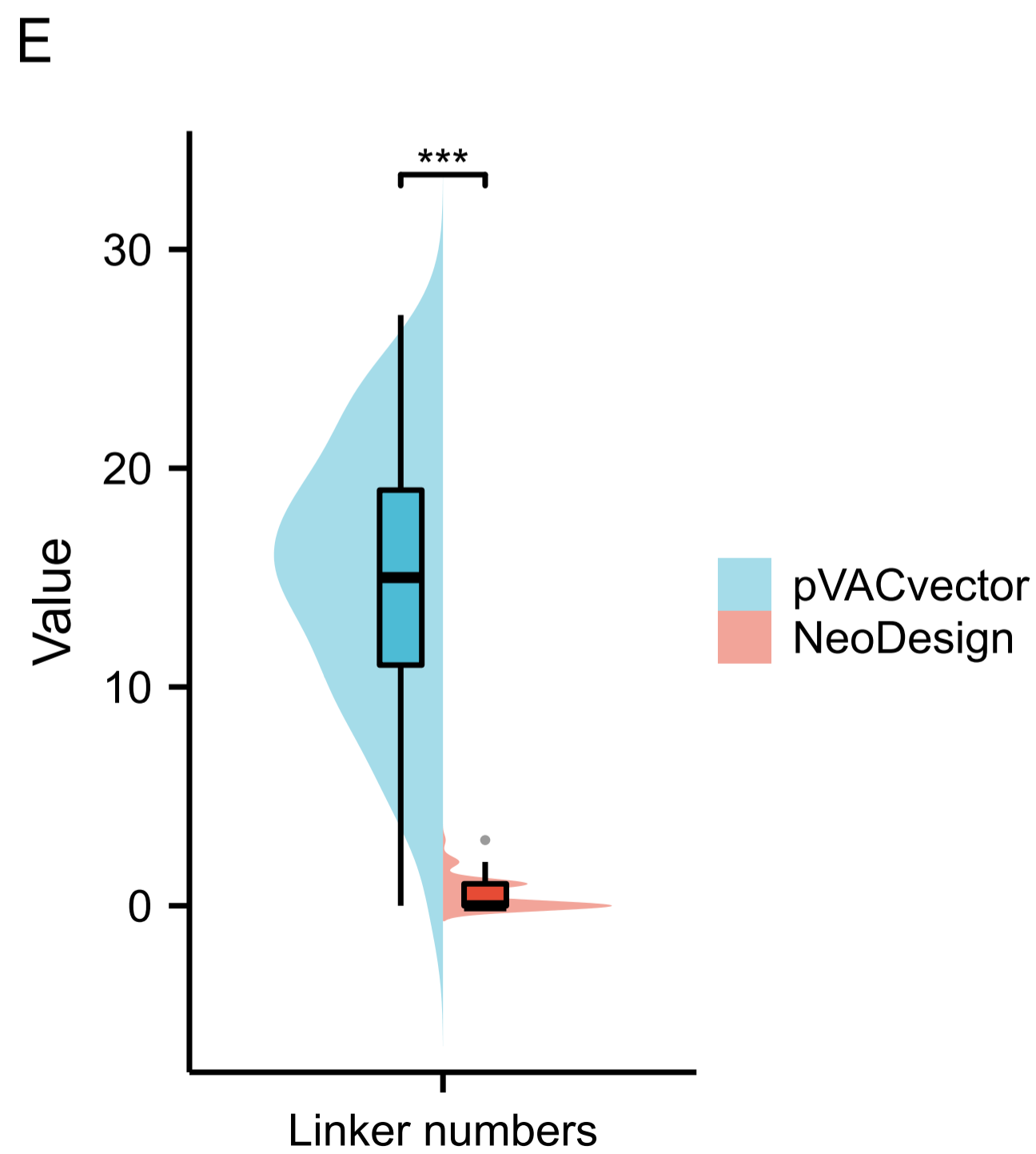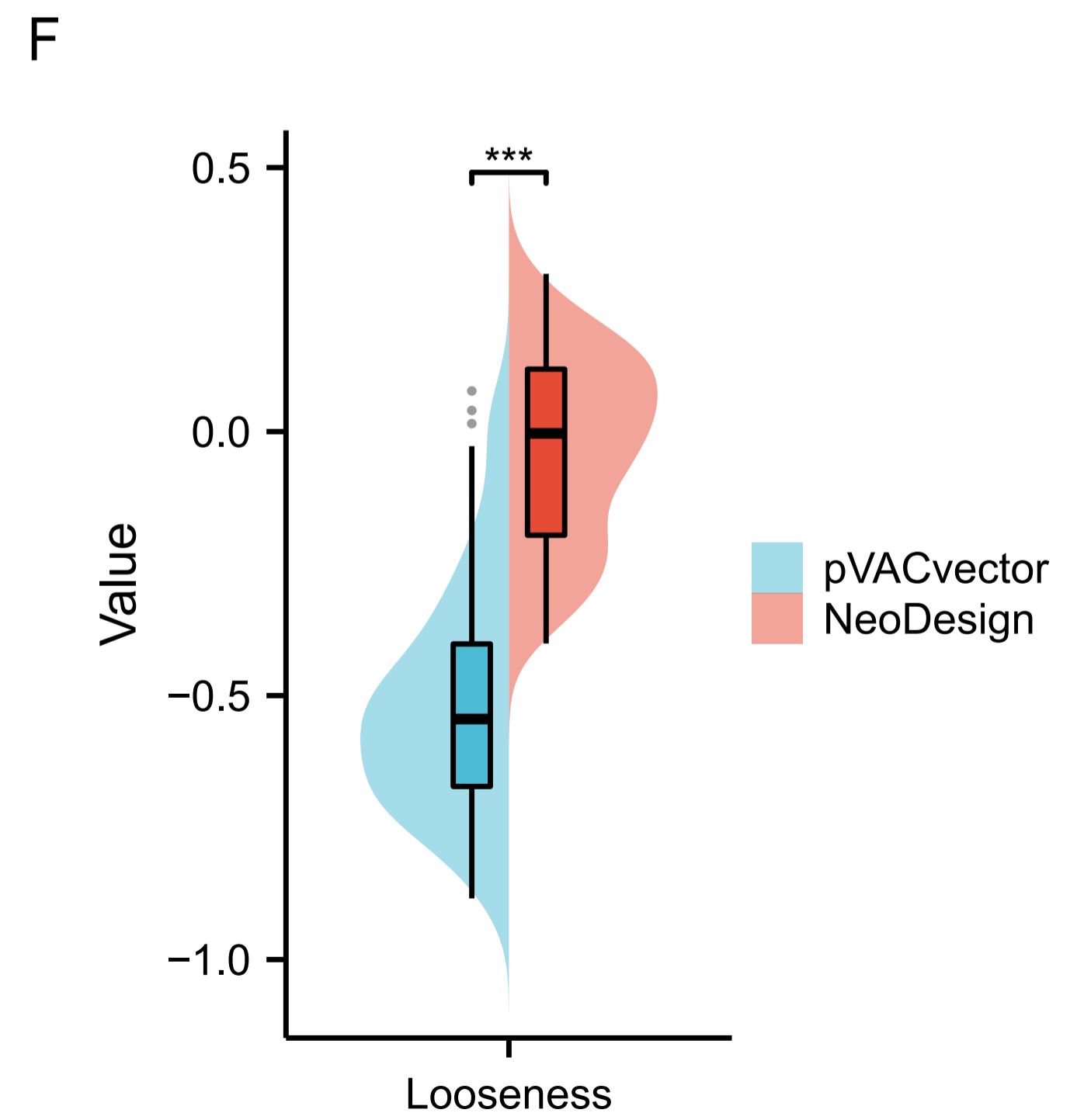

Supplement: btae585_Supplementary_Data [file btae585_supplementary_data.zip › Supplementary Figure 12.pdf]

A

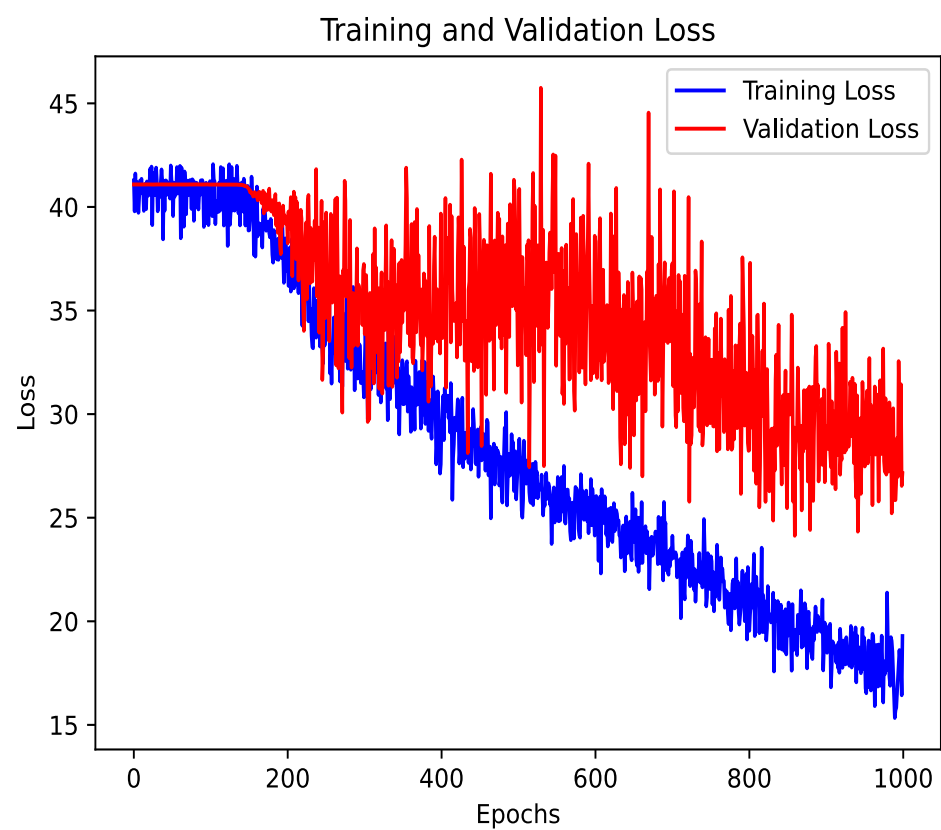

B

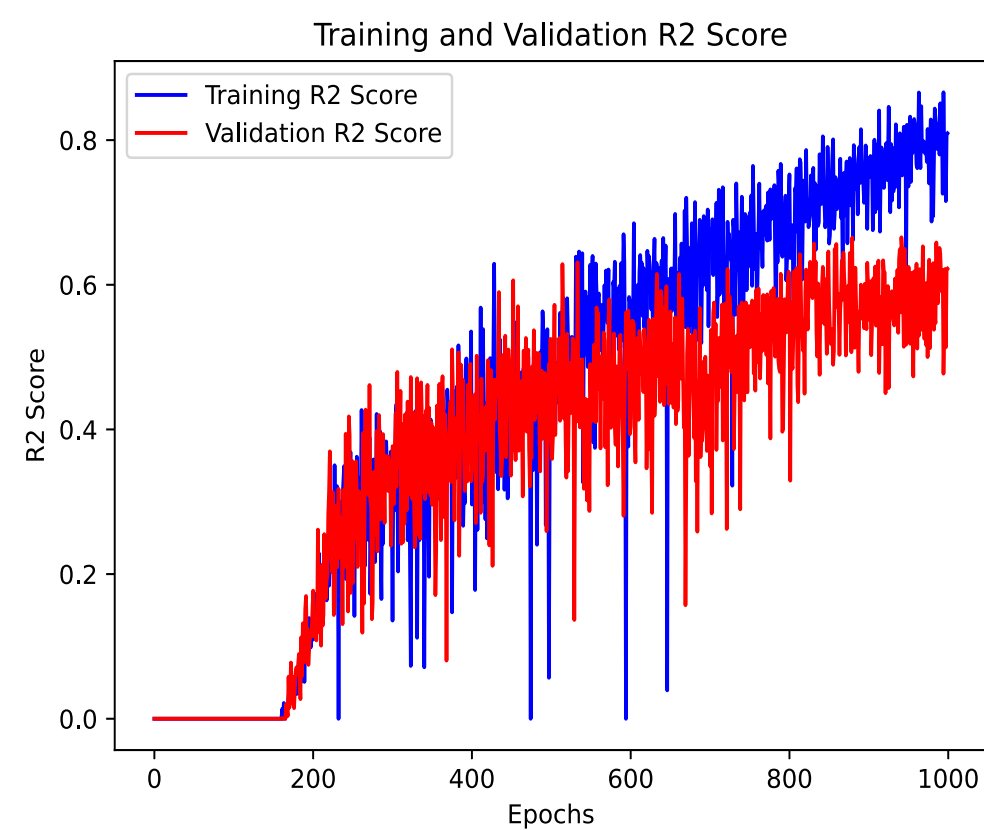

C

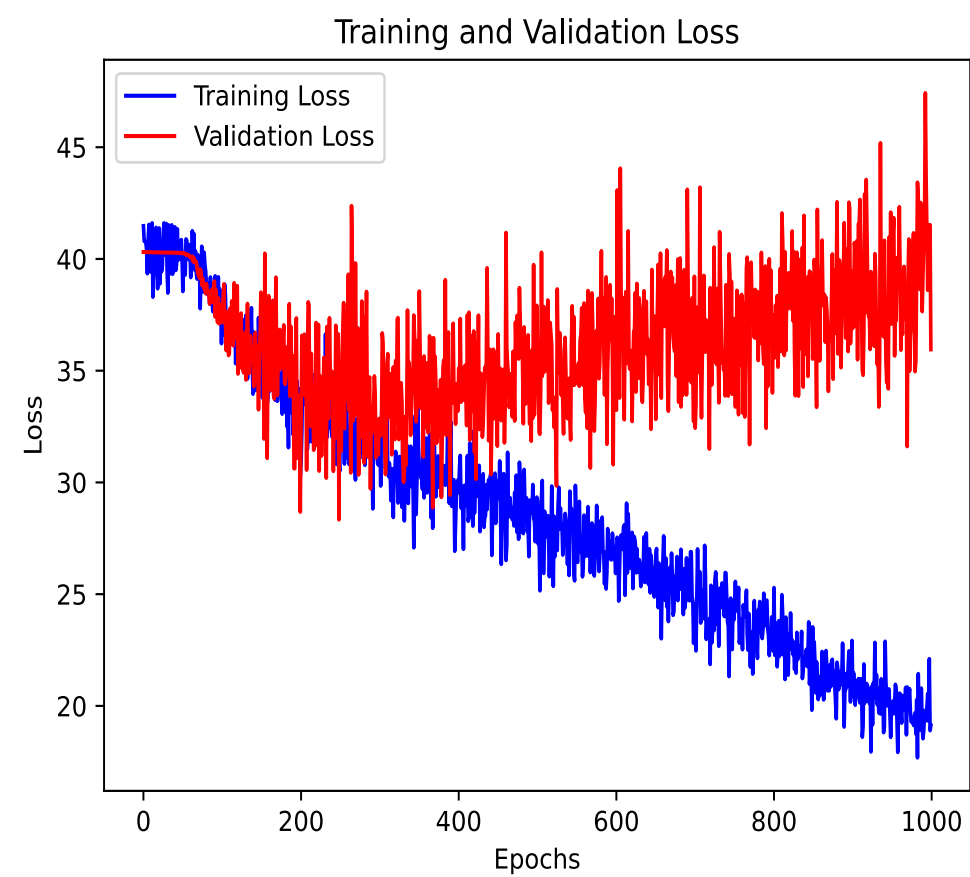

D

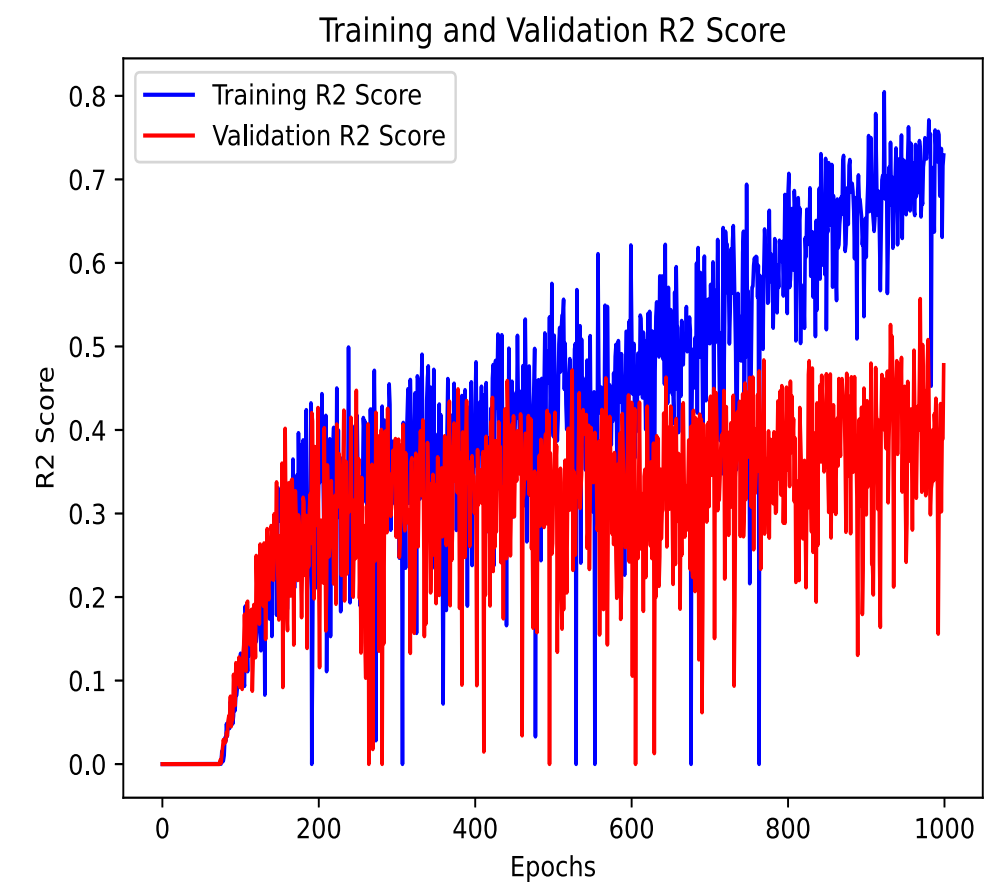

Supplement: btae585_Supplementary_Data [file btae585_supplementary_data.zip › Supplementary Figure 6.pdf]

A

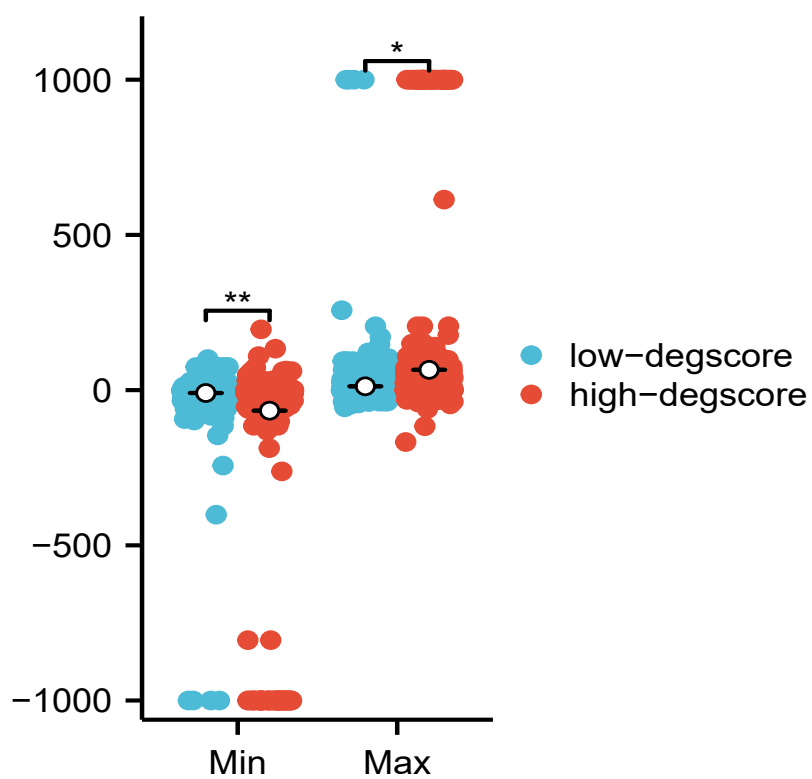

B

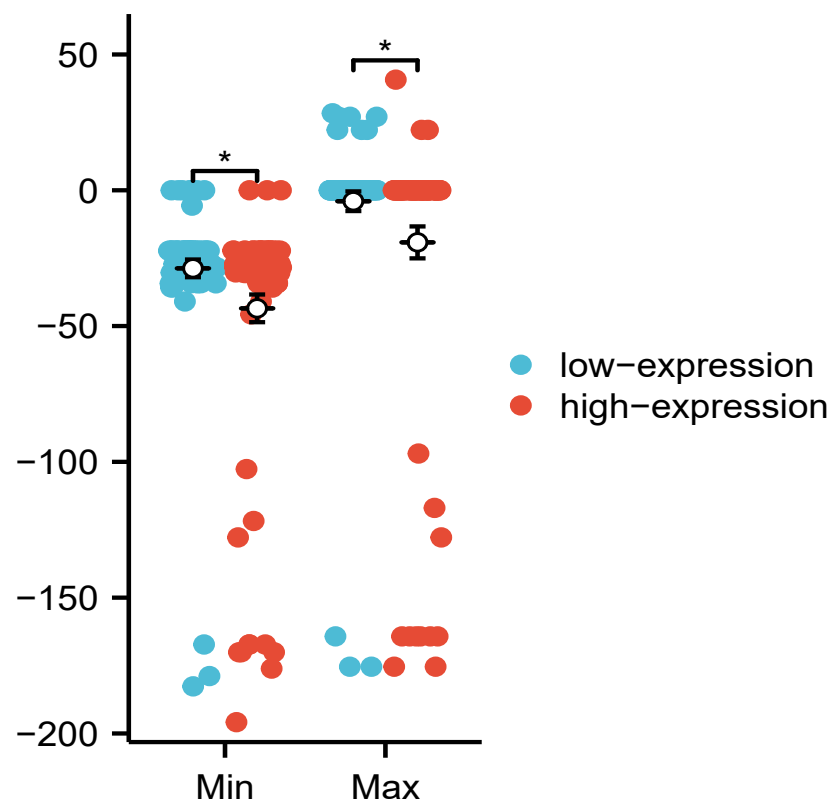

C

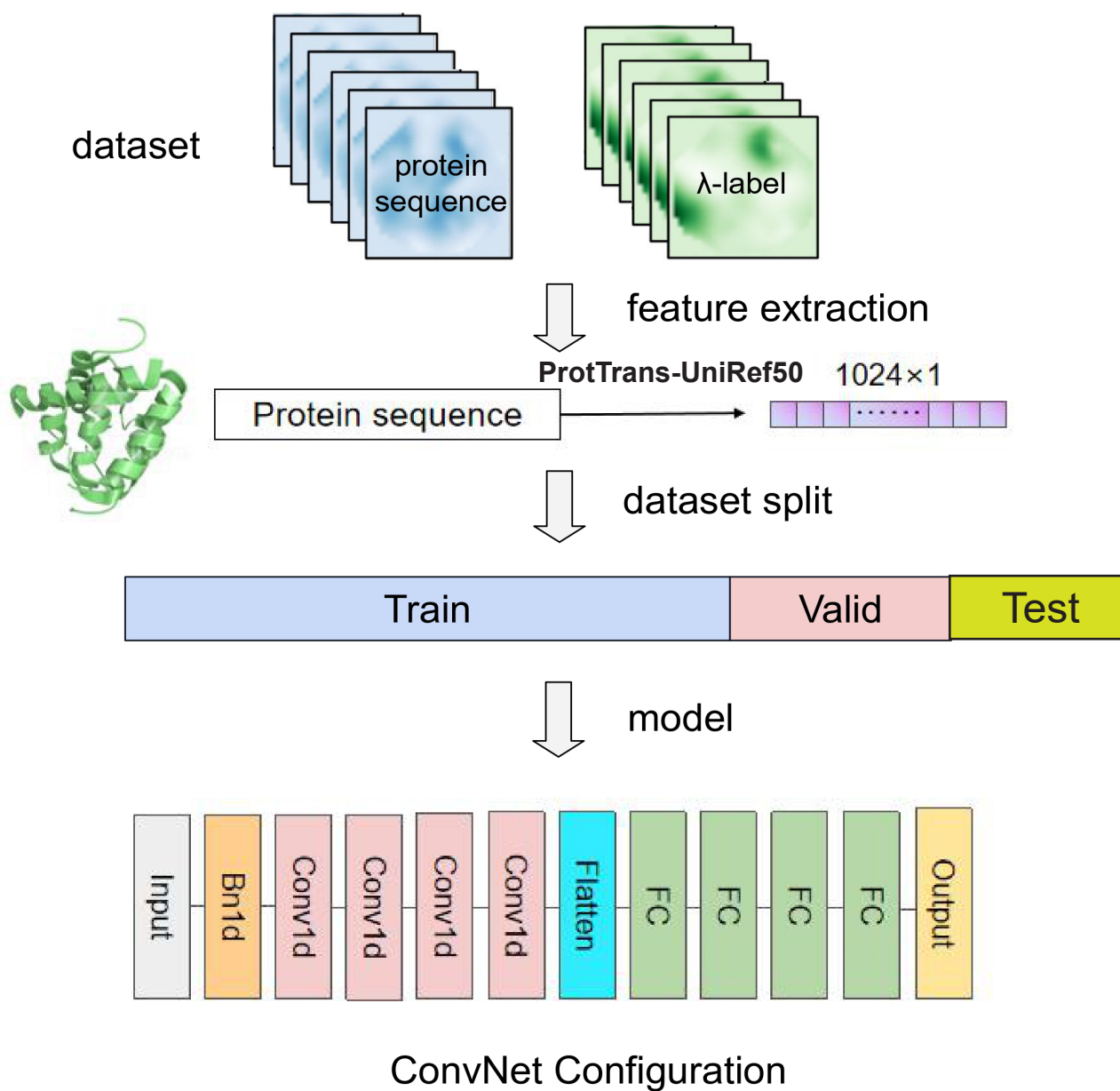

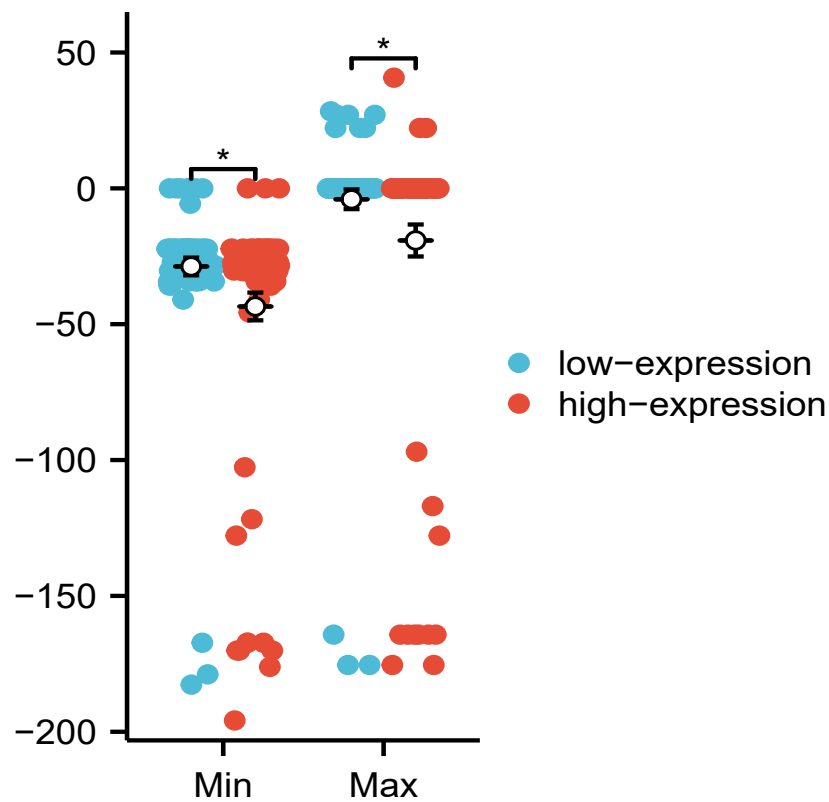

Supplement: btae585_Supplementary_Data [file btae585_supplementary_data.zip › Supplementary Figure 5.pdf]

A

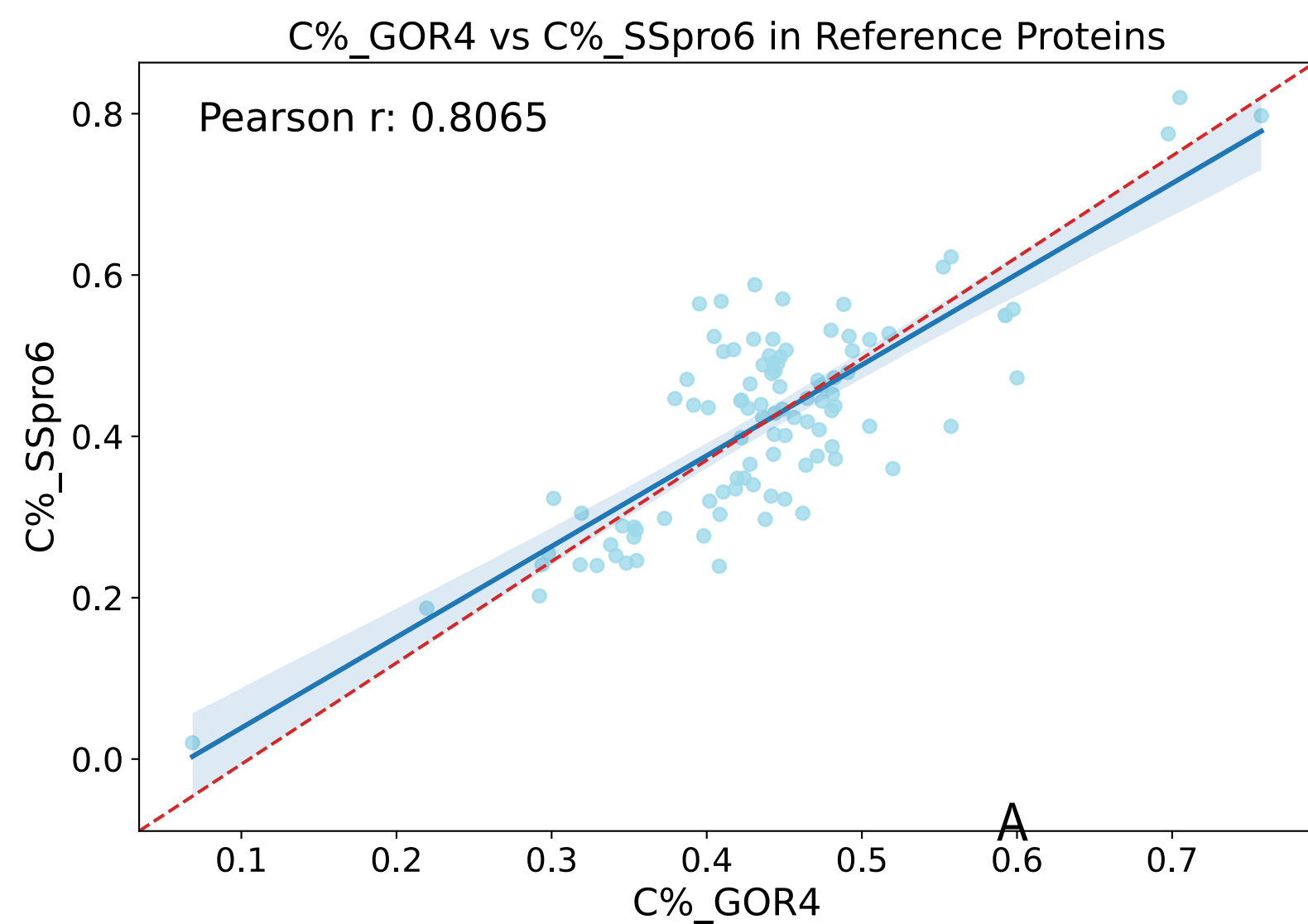

B

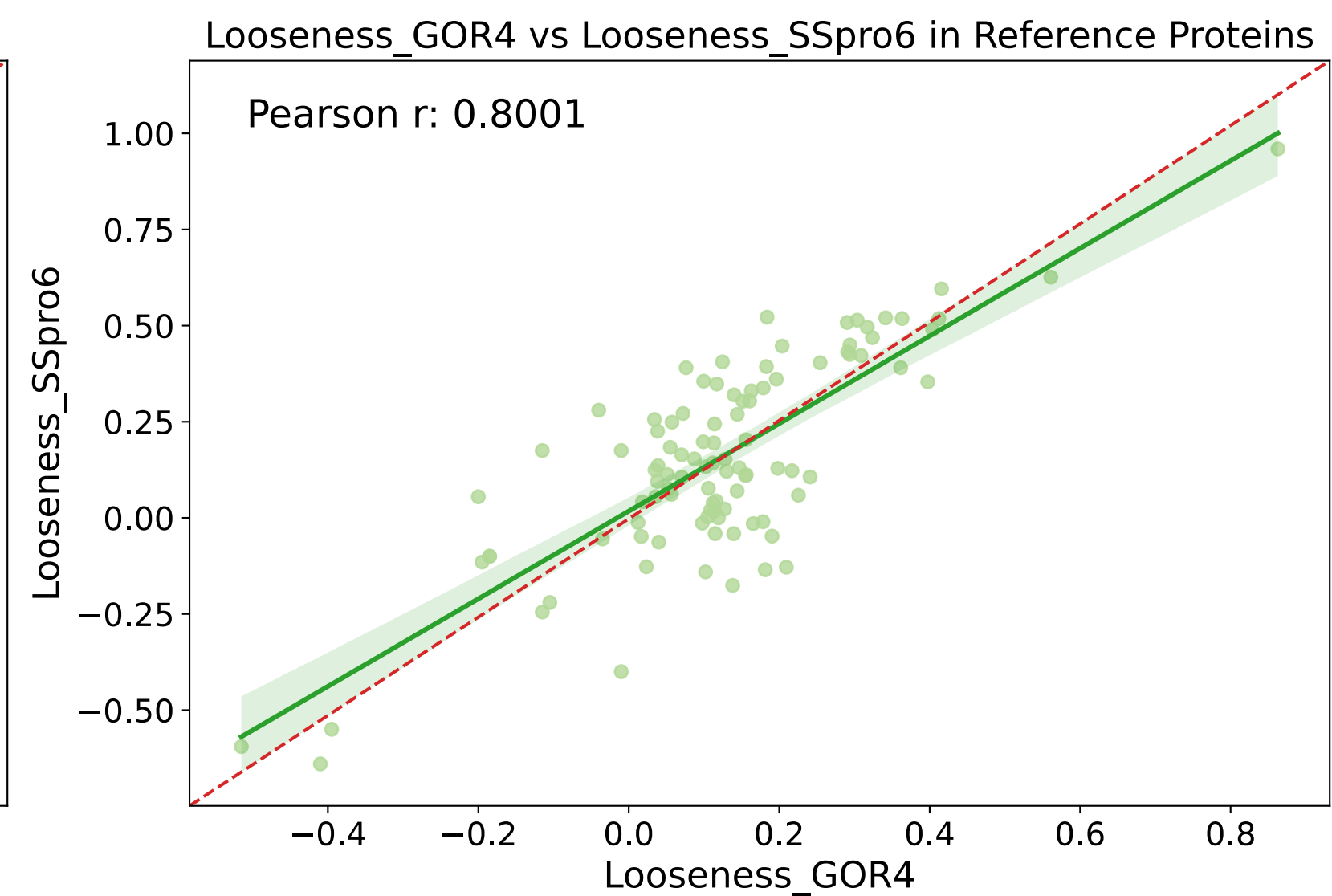

C

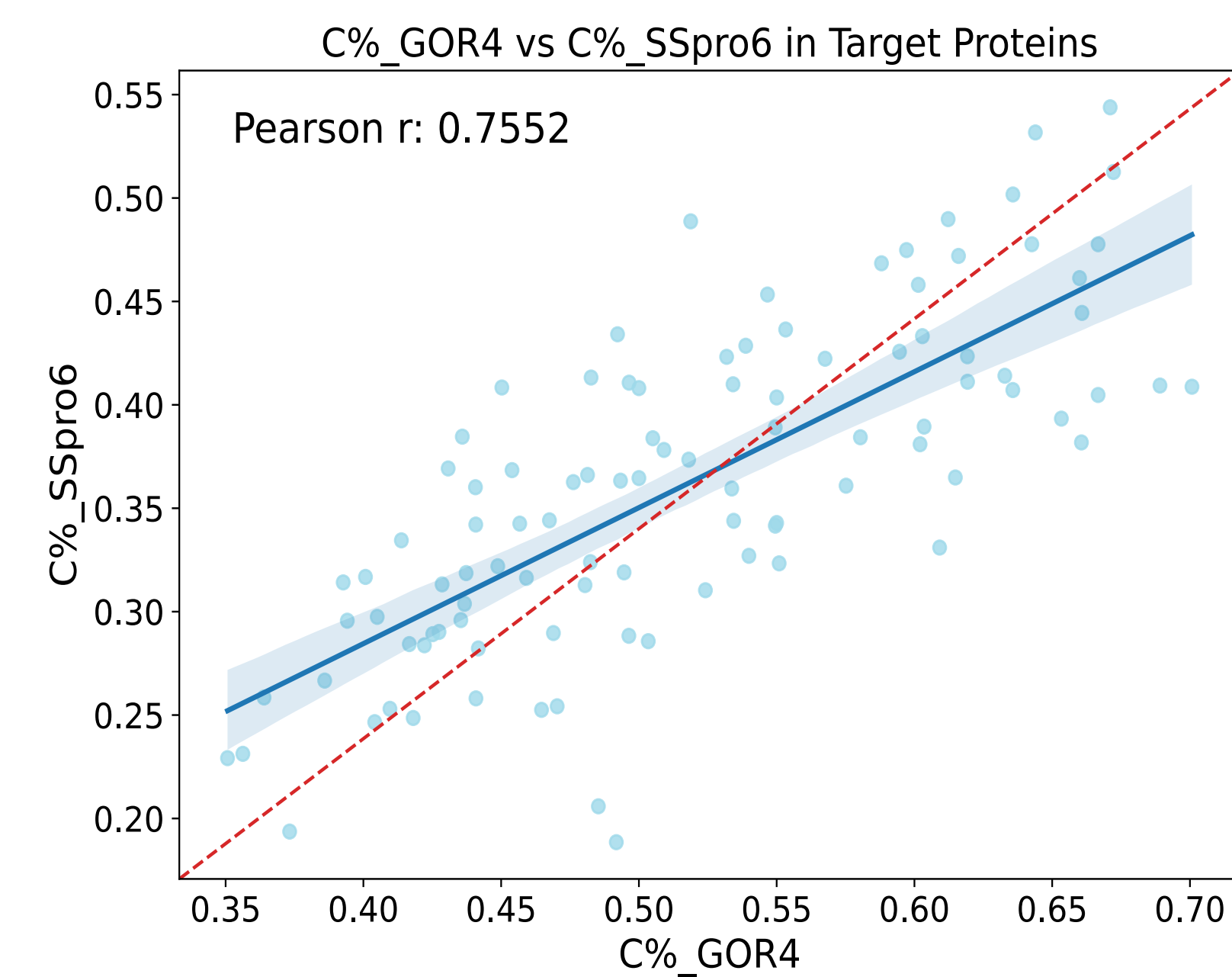

D

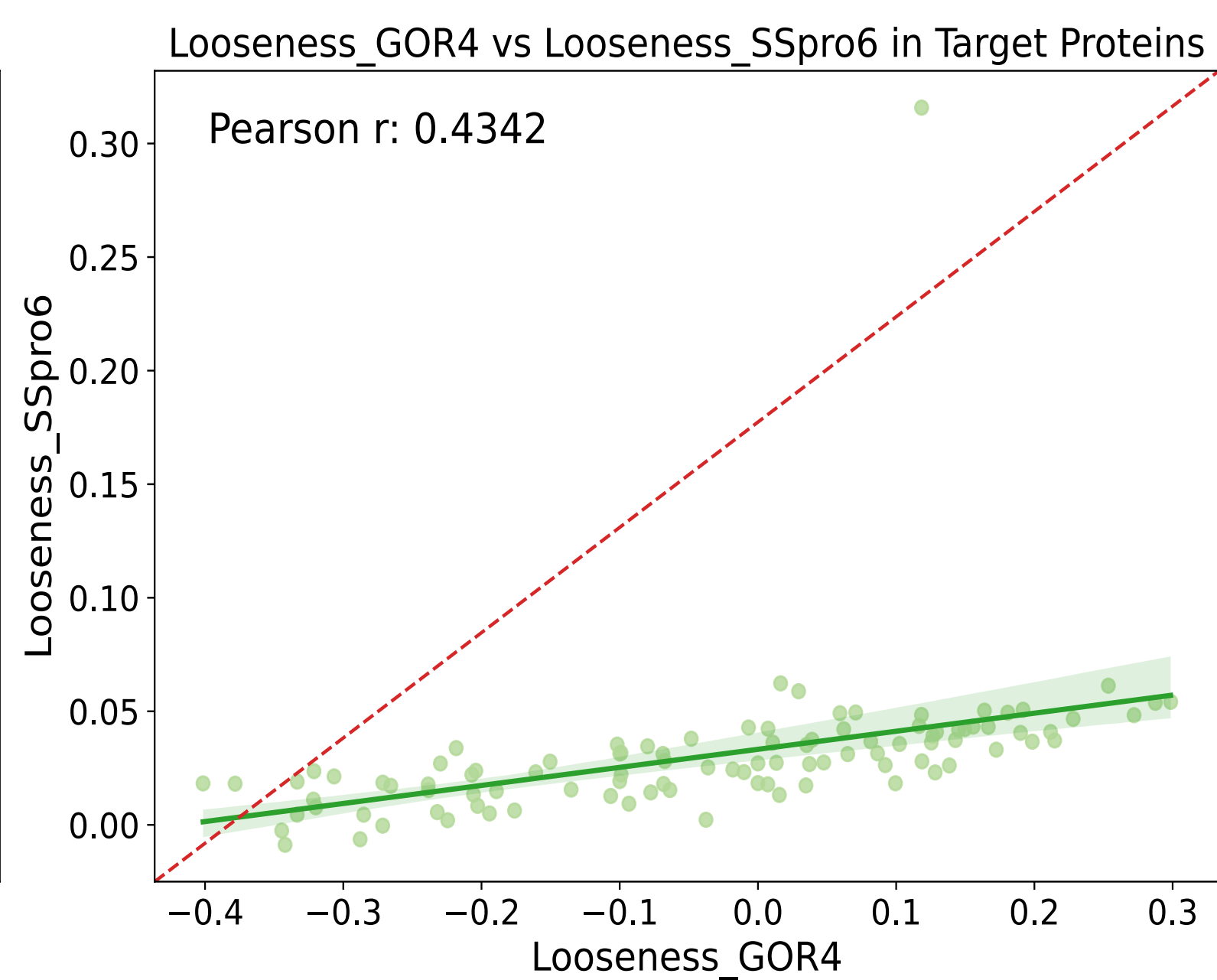

E

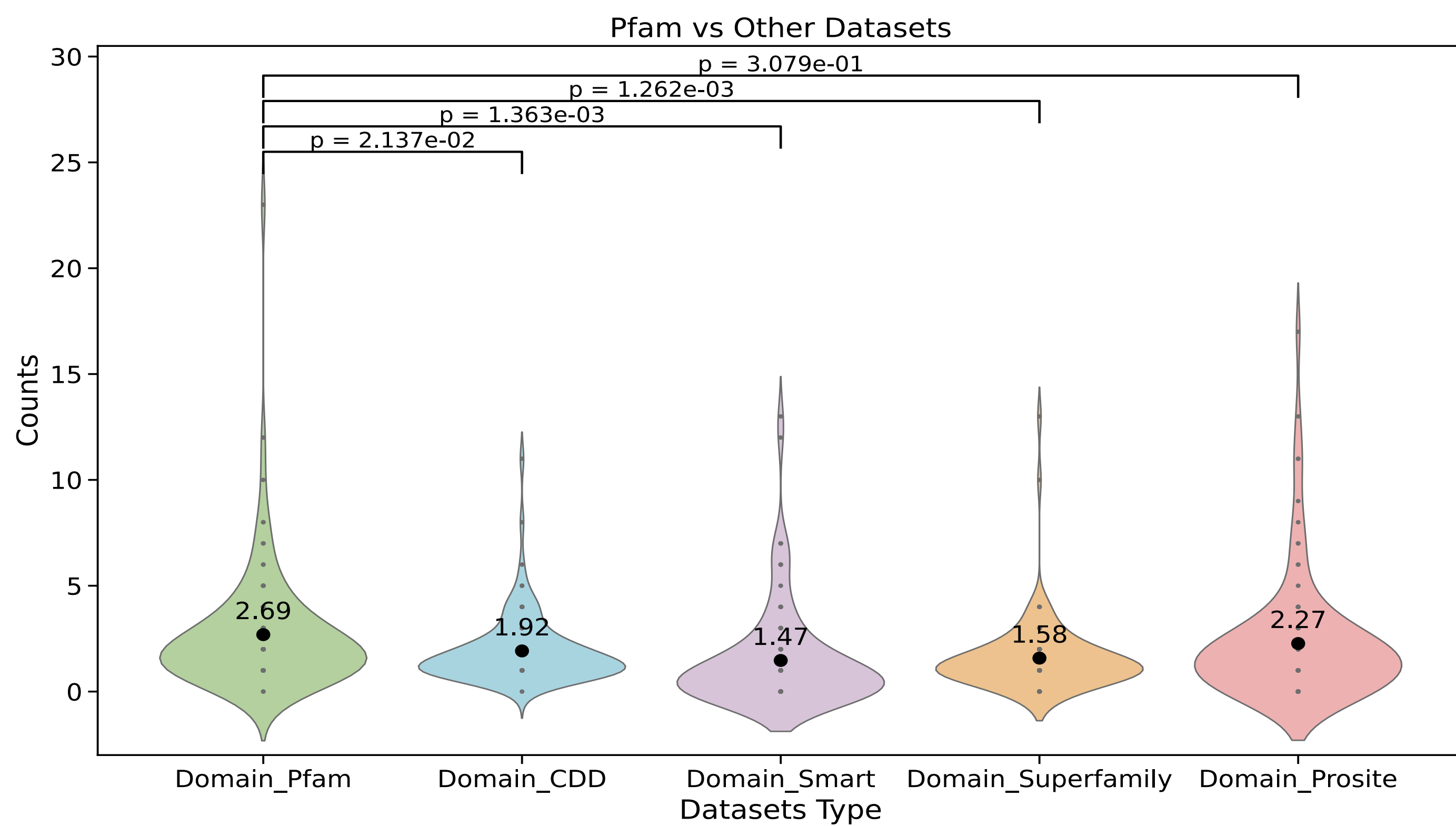

Supplement: btae585_Supplementary_Data [file btae585_supplementary_data.zip › Supplementary Figure 13.pdf]

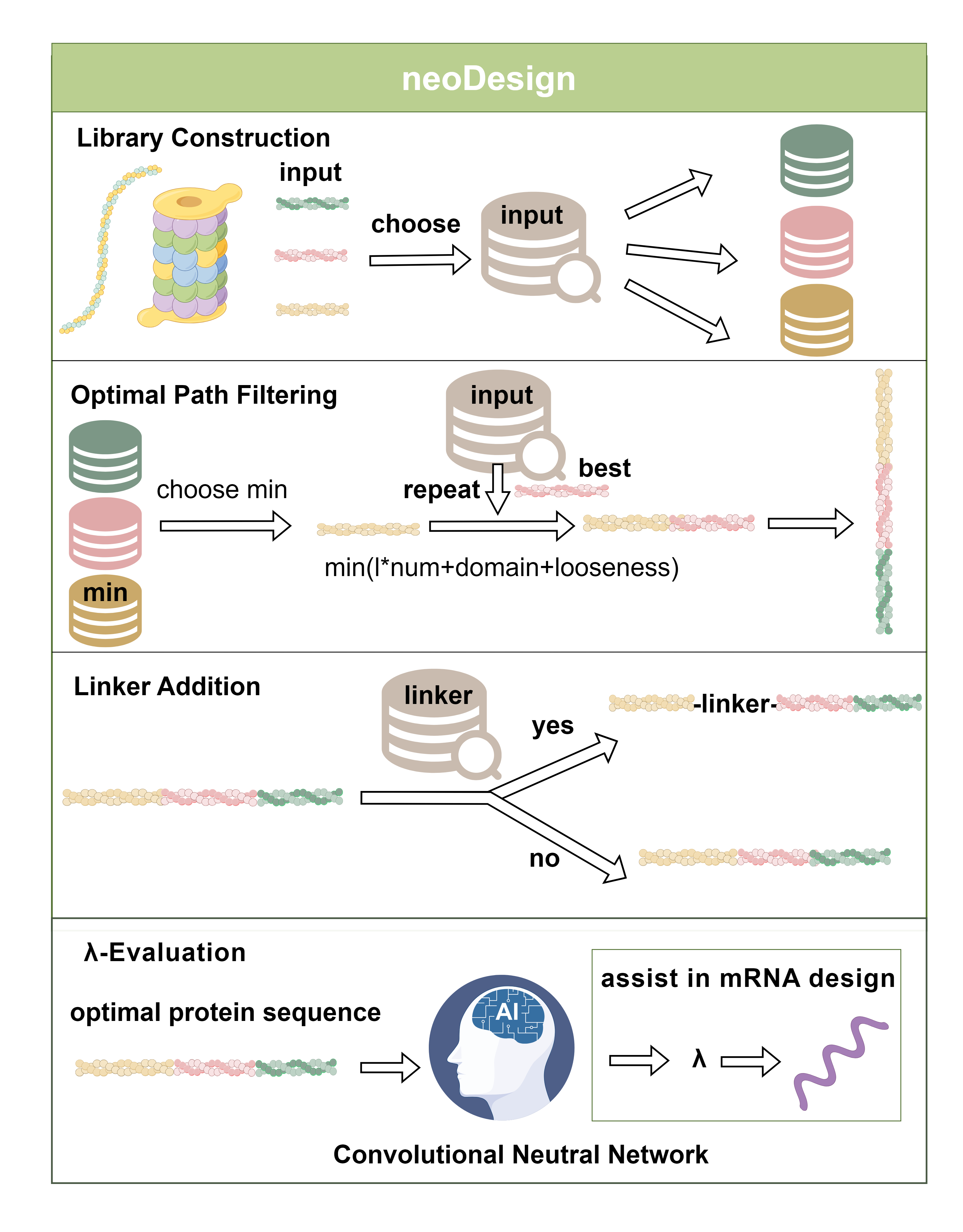

Supplement: btae585_Supplementary_Data [file btae585_supplementary_data.zip › figure1.png]

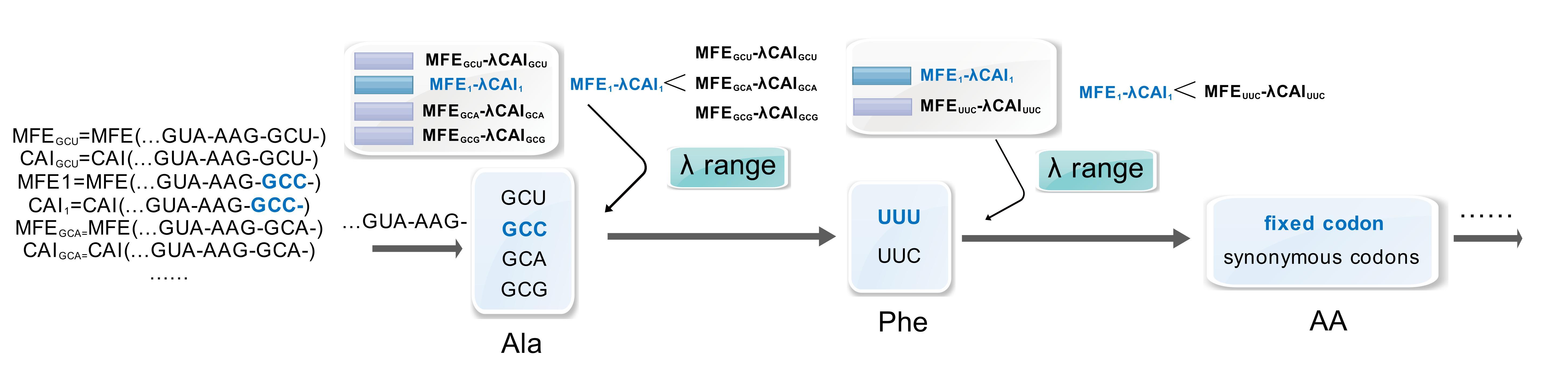

Supplement: btae585_Supplementary_Data [file btae585_supplementary_data.zip › Supplementary Figure 2.jpeg]

Supplementary Figure 1

A

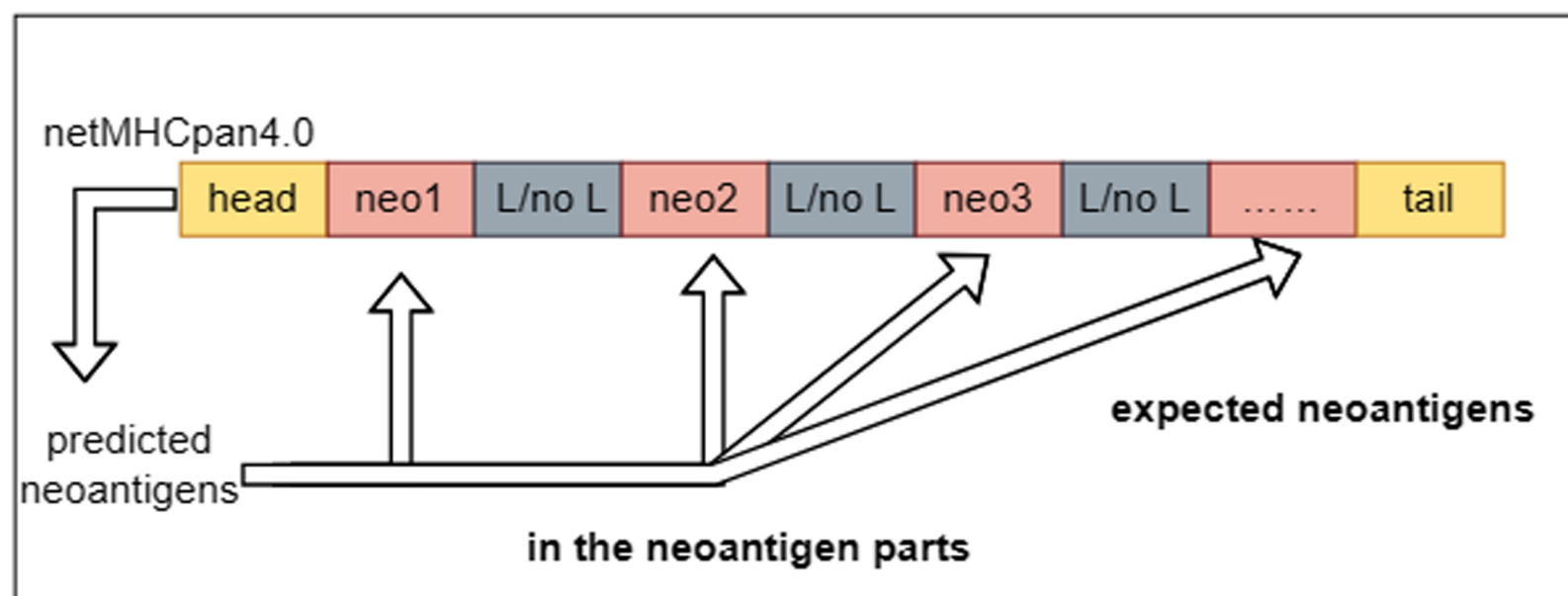

B

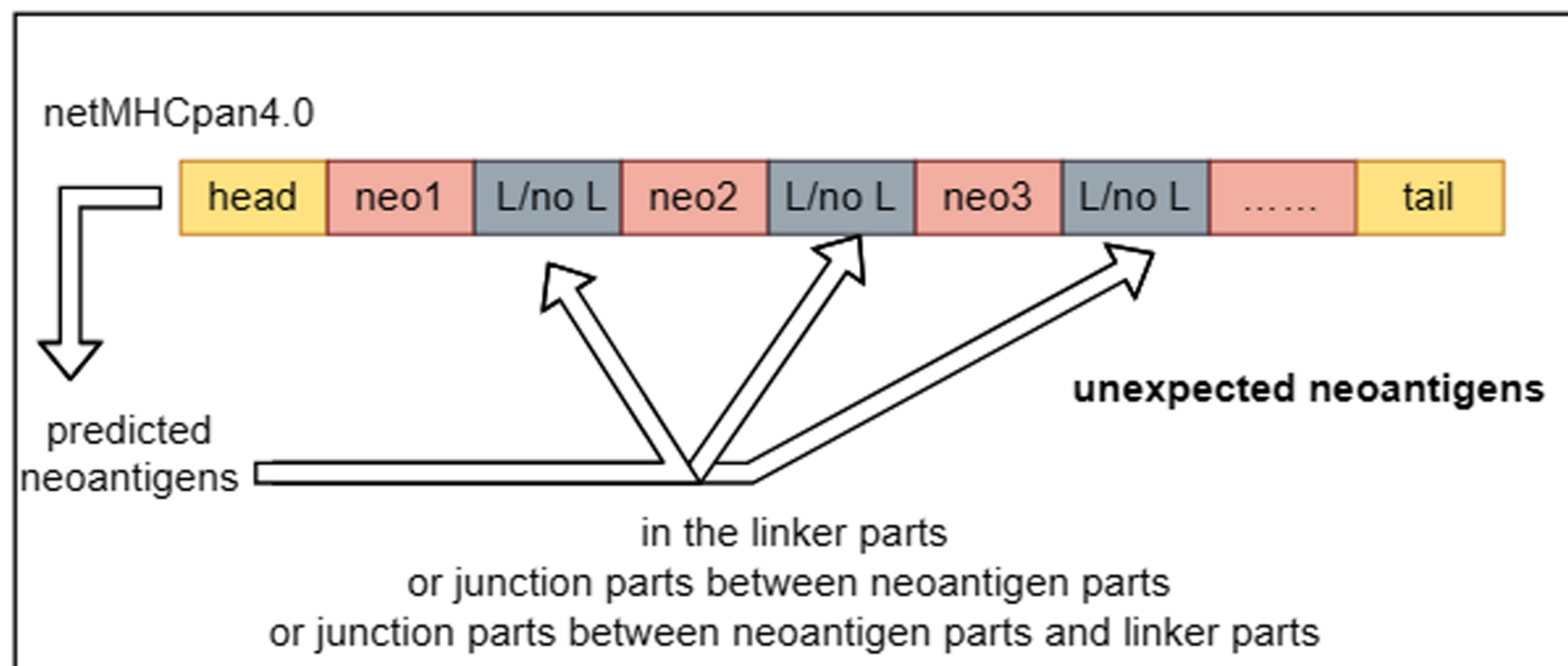

Supplement: btae585_Supplementary_Data [file btae585_supplementary_data.zip › Supplementary Figure 1.pdf]
