# Supplementary material for: NeoDesign: a computational tool for optimal selection of polyvalent neoantigen combinations
Source: Bioinformatics. 2024 Sep 27;40(10):btae585. doi: 10.1093/bioinformatics/btae585 (PMC11471261; doi:10.1093/bioinformatics/btae585)
Supplement: btae585_Supplementary_Data [file btae585_supplementary_data.zip › all_figures.zip!/all_figures/Supplementary Figure 4.pdf]

Figure 3

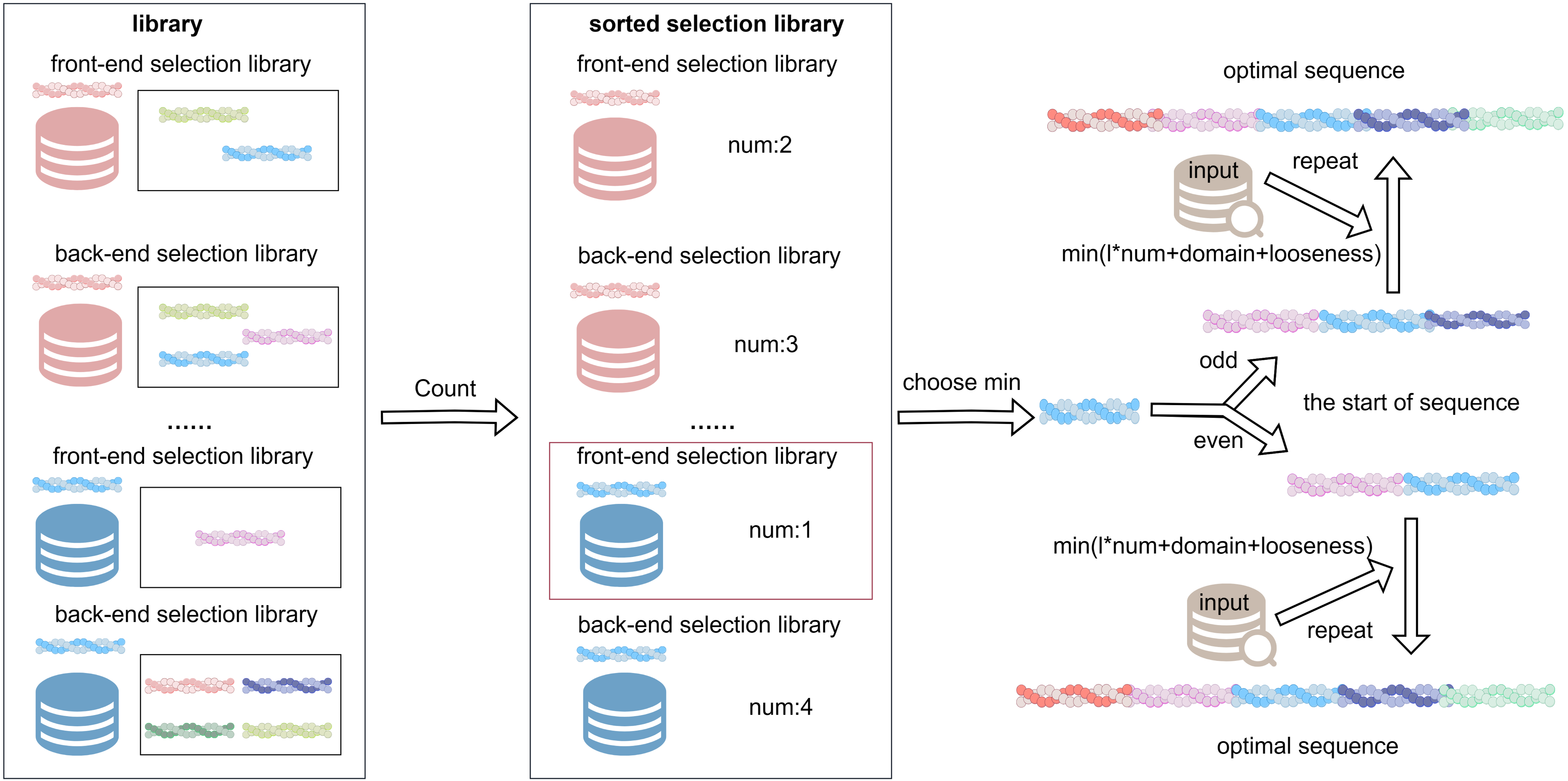

library
front-end selection library
back-end selection library
......
front-end selection library
back-end selection library
Count
sorted selection library
front-end selection library
num:2
back-end selection library
num:3
......
front-end selection library
num:1
back-end selection library
num:4
choose min
optimal sequence
input
repeat
min(l*num+domain+looseness)
odd
the start of sequence
even
min(l*num+domain+looseness)
input
repeat
optimal sequence
