# Supplementary material for: NeoDesign: a computational tool for optimal selection of polyvalent neoantigen combinations
Source: Bioinformatics. 2024 Sep 27;40(10):btae585. doi: 10.1093/bioinformatics/btae585 (PMC11471261; doi:10.1093/bioinformatics/btae585)
Supplement: btae585_Supplementary_Data [file btae585_supplementary_data.zip › all_figures.zip!/all_figures/Supplementary Figure 7.pdf]

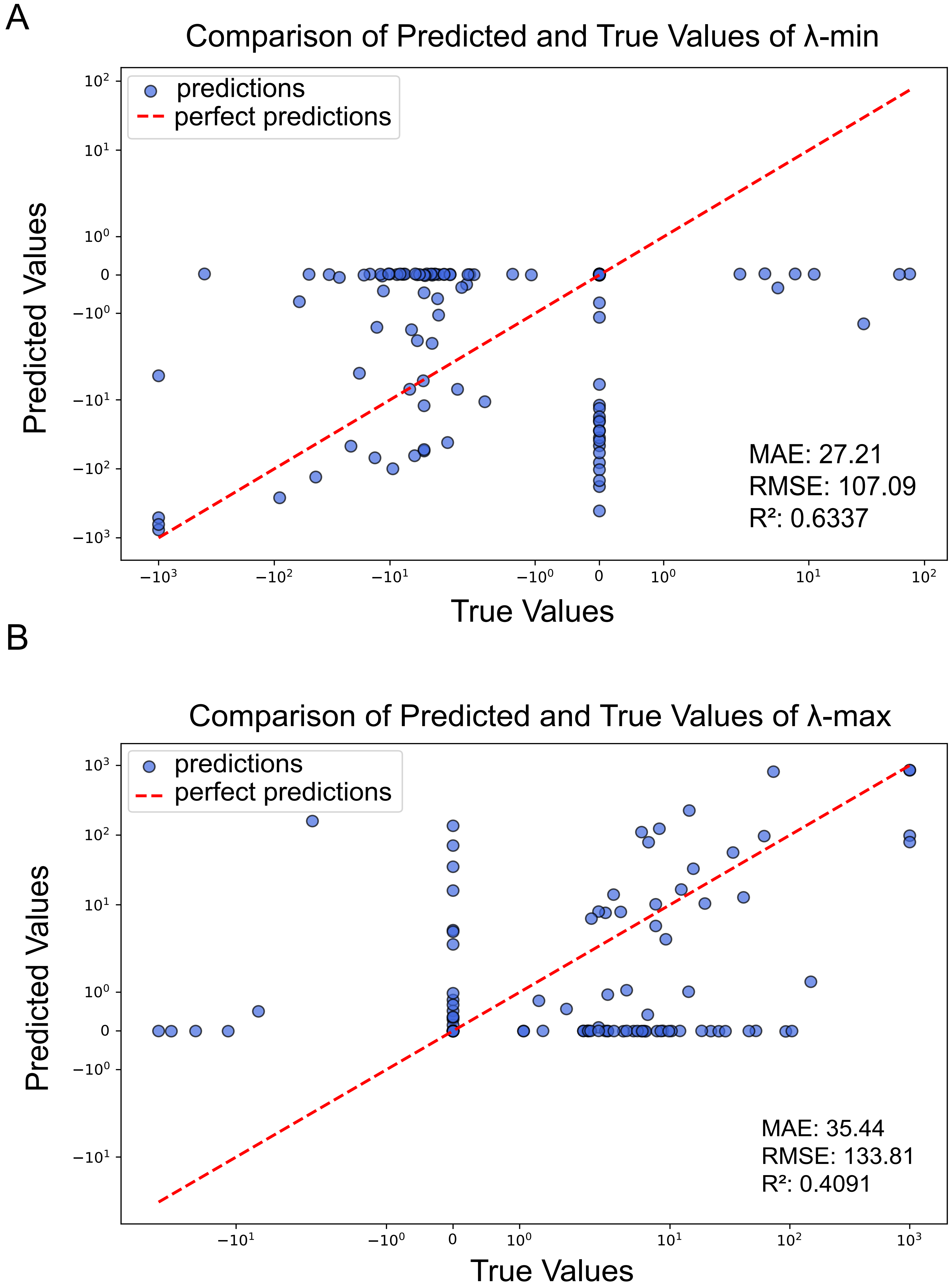
A
Comparison of Predicted and True Values of λ-min
predictions
perfect predictions
Predicted Values
True Values
MAE: 27.21
RMSE: 107.09
R²: 0.6337
B
Comparison of Predicted and True Values of λ-max
predictions
perfect predictions
Predicted Values
True Values
MAE: 35.44
RMSE: 133.81
R²: 0.4091
